# Development of Phenalenone-Triazolium Salt Derivatives for aPDT: Synthesis and Antibacterial Screening

**DOI:** 10.3390/antibiotics10060626

**Published:** 2021-05-24

**Authors:** Jérémy Godard, Dáire Gibbons, Stéphanie Leroy-Lhez, René M. Williams, Nicolas Villandier, Tan-Sothéa Ouk, Frédérique Brégier, Vincent Sol

**Affiliations:** 1 Faculté des Sciences et Techniques, Université de Limoges, PEIRENE, EA 7500, 123 Avenue Albert Thomas, CEDEX, 87060 Limoges, France; jeremy.godard@unilim.fr (J.G.); daire.gibbons@unilim.fr (D.G.); stephanie.lhez@unilim.fr (S.L.-L.); nicolas.villandier@unilim.fr (N.V.); tan-sothea.ouk@unilim.fr (T.-S.O.); frederique.bregier@unilim.fr (F.B.); 2 Molecular Photonics Group, Van ’t Hoff Institute for Molecular Sciences (HIMS), Universiteit van Amsterdam, Science Park 904, 1098 XH Amsterdam, The Netherlands; R.M.Williams@uva.nl

**Keywords:** phenalenone, triazolium salt, bacteria photoinactivation, PDT, photosensitizers

## Abstract

The increasing number of hospital-acquired infections demand the development of innovative antimicrobial treatments. Antimicrobial photodynamic therapy (aPDT) is a versatile technique which relies on the production of reactive oxygen species (ROS) generated by light-irradiated photosensitizers (PS) in the presence of oxygen (O_2_). 1*H*-Phenalen-1-one is a very efficient photosensitizer known for its high singlet oxygen quantum yield and its antimicrobial potential in aPDT when covalently bound to quaternary ammonium groups. Triazolium salts are stable aromatic quaternary ammonium salts that recently appeared as interesting moieties endowed with antimicrobial activities. The coupling between phenalenone and triazolium groups bearing various substituents was realized by copper-catalyzed azide-alkyne cycloaddition followed by alkylation with methyl iodide or 2-(bromomethyl)-1*H*-phenalen-1-one. As expected, most of the compounds retained the initial singlet oxygen quantum yield, close to unity. Minimum inhibitory concentrations (MIC) of 14 new phenalenone-triazolium salt derivatives and 2 phenalenone-triazole derivatives were determined against 6 bacterial strains (Gram-negatives and Gram-positives species). Most of these PS showed significant photoinactivation activities, the strongest effects being observed against Gram-positive strains with as low as submicromolar MIC values.

## 1. Introduction

The rapid global spread of multidrug-resistant bacteria is a growing health concern. Nosocomial infections occur worldwide; 7 to 10% of the total hospitalized patients acquire a nosocomial infection with a particular incidence of surgical-site infections [1]. *Staphylococcus* spp., *Escherichia coli*, *Pseudomonas aeruginosa*, *Klebsiella pneumoniae*, *Enterobacter* spp., *Enterococcus* spp., and *Acinetobacter* spp. are the most commonly isolated bacteria, causing lung, urinary tract, and bloodstream infections [2,3], and previously “benign” strains, such as *Bacillus cereus*, tend to become more dangerous [4]. In order to counter the propagation of these pathogenic bacteria, alternative treatments have been developed which allow to bypass the use of antibiotics: immunotherapy [5], phage therapy [6], and antimicrobial photodynamic therapy (aPDT) [7,8].

**Figure 1:**
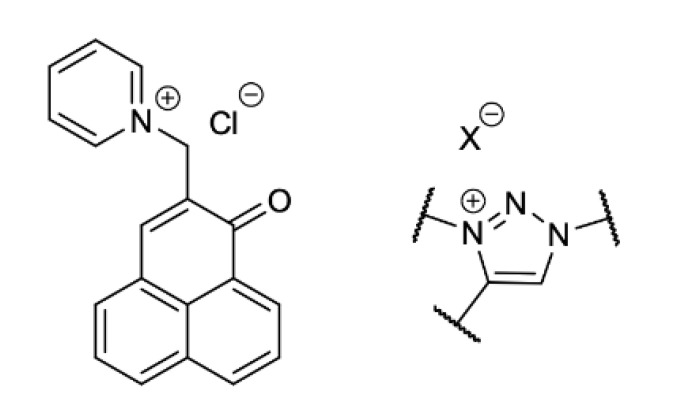
Structure of SAPyR (left) and 1,3,4-trisubstituted-1,2,3-triazolium salt (right).

Photodynamic therapy (PDT) involves the use of photosensitizers, which, upon excitation with visible light and in the presence of dioxygen, give rise to organic free radicals and reactive oxygen species (ROS) or to singlet oxygen, according to type I and type II PDT mechanisms, respectively. These two mechanisms lead to the photodynamic inactivation (PDI) of bacteria and, for the most potent photosensitizers, bacterial cell death [9]. In addition, no bacterial resistance against these ROS has been observed, constituting a huge advantage in comparison with the use of antibiotics. Photosensitizers displayed strong photobactericidal activities when covalently bound to specific permeabilizing agents, positively charged or bacteria-targeting moieties, e.g., pyridylporphyrin bound to polymyxins [10,11] or phenalenone-bound pyridinium, also known as SAPyR (Figure 1). The latter is of particular interest, due to its easy synthesis, its high singlet oxygen quantum yield, its stability toward Hoffman elimination as compared with non-aromatic quaternary ammonium salts (QAS), and its strong phototoxic activity against a wide range of bacterial species [12,13,14]. However, this compound presents a major drawback, since it is difficult to add substituents which could modulate its photodynamic activity.

Recently, triazolium salts (Figure 1), another class of aromatic QAS, were evaluated as very promising antimicrobial agents, effective against Gram-positive and Gram-negative bacteria as well as several yeast species [15]. Triazolium salts are easy to synthesize and, in addition, it is possible to graft three different functional groups in a regioselective manner. Moreover, to the best of our knowledge, this permanent cationic charge on the triazolium ring has never been coupled with a photosensitizer for antimicrobial applications. In connection with our research program on the design and development of new photosensitizers for photoantimicrobial applications [16,17], we report herein the synthesis and characterization of phenalenone derivatives bearing 1,3,4-trisubstituted-1,2,3-triazolium salts. The photoinactivation properties of these new photosensitizers were evaluated against six bacterial strains.

## 2. Results and Discussion

Seven types of triazole substituents were chosen for these assays. The adamantane group (Ada), a strongly apolar moiety whose hydrophobicity could counterbalance the hydrophilicity of the triazolium group, exhibited a medium to good antimicrobial activity toward a large variety of pathogens [18]. The benzyl moiety was also chosen because of its hydrophobicity and its good antimicrobial activity associated with triazolium salts [15]. Glucose (Glc) and primary ammonium (NH_3_^+^) derivatives were chosen as neutral and cationic hydrophilic substituents, respectively, in order to enhance the solubility in aqueous media even in the absence of triazolium, making it possible to compare triazole and triazolium activities. Their peracetylated (Glc(OAc)_4_) and t-butoxycarbonyl (BOC) derivatives, despite the lack of literature about noticeable antibacterial effects, were tested to compare their activities with their unprotected glucose and amino counterparts. Finally, a dimer of phenalenone (PN) was built in order to appreciate the impact of the number of photosensitizer moieties on the antibacterial activity.

**Figure 2:**
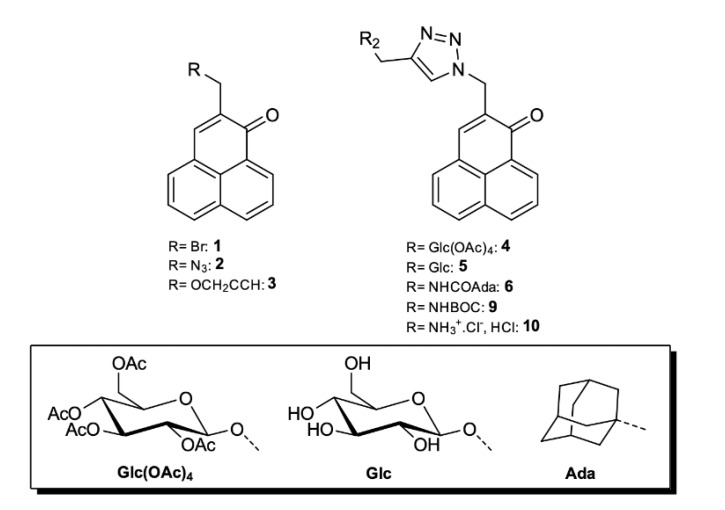
Structure of phenalenone derivatives used as precursors for the triazolium salt synthesis.

**Scheme 1:**
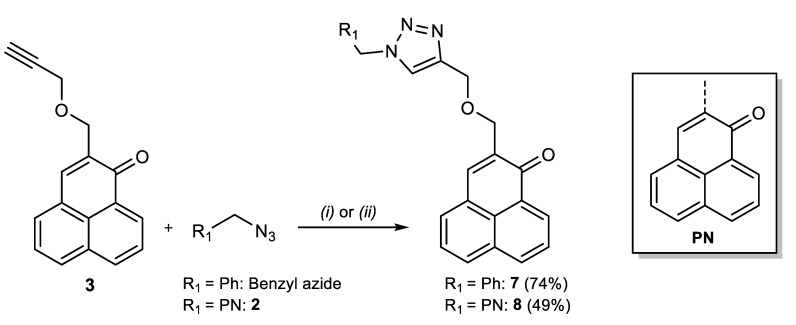
Synthesis of compounds **7** and **8** from propargyl precursor **3** by CuAAC. Reaction conditions: (i) Benzyle azide (2 eq.), CuI (0.02 eq.), diisopropylethylamine (0.04 eq.), acetic acid (0.02 eq.), 55 h, r.t. (ii) **2** (1 eq.), and CuSO_4_.5H_2_O (0.2 eq.) in tBuOH/H_2_O 1:1, 72 h, r.t.

Phenalenone derivatives **1** to **6**, **9** and **10** were synthetized as previously described [19], and compounds **7** and **8** were obtained from compound **3** (Figure 2). Briefly, the copper-catalyzed azide-alkyne cycloaddition (CuAAC) reactions were carried out in CH_2_Cl_2_ using copper iodide, diisopropylethylamine and acetic acid as catalysts [20], a method presenting the advantage to be easily feasible in organic media. Furthermore, **7** was synthesized by this way from **3** in good yield (74%), contrary to **8** (18% yield after 72 h). The classical synthesis in aqueous media with copper sulfate and sodium ascorbate [21] gave compound **8** in better yield (49%) (Scheme 1).

Even if a large variety of compounds can be used as alkylating agents, only two of them have been tested, namely, methyl iodide, the simplest one, and 2-(bromomethyl)-1*H*-phenalene-1-one (**1**). All the reactants were soluble in acetonitrile but, as expected, **1** was less reactive than methyl iodide, leading to lower yields despite longer reaction times. All the alkylated compounds were purified by column chromatography, except **5a** and **5b**, which are highly polar compounds. Finally, tert-butyl carbamates (BOC) of compounds **9a** and **9b** were cleaved as previously described for **9** [19] to obtain the primary amine hydrochloride derivatives **10a** and **10b** in excellent yields (99 and 97%, respectively) (Table 1). All of these compounds were fully characterized by NMR and HRMS (Figures S1–S48 in Supplementary Materials), their octanol/water partition coefficient (log P) was estimated using MarvinSketch Software v. 21.2 (Table 1), and their photophysical properties including singlet oxygen quantum yield (ΦΔ) measurements were completely studied in water (Table 1 and Table S1 in Supplementary Materials). As expected, all the compounds showed a very low fluorescence quantum yield, and most of the compounds exhibited a singlet oxygen quantum yield close to unity, but surprisingly, singlet oxygen phosphorescence at 1270 nm was hardly observed for dimers and trimers of PN, which could suggest an important π-stacking effect between multiple planar PN moieties. Due to the polar nature of water and the low lifetime of singlet oxygen in this solvent, further measurements in less polar solvents are required to confirm this hypothesis.

**Scheme 2:**
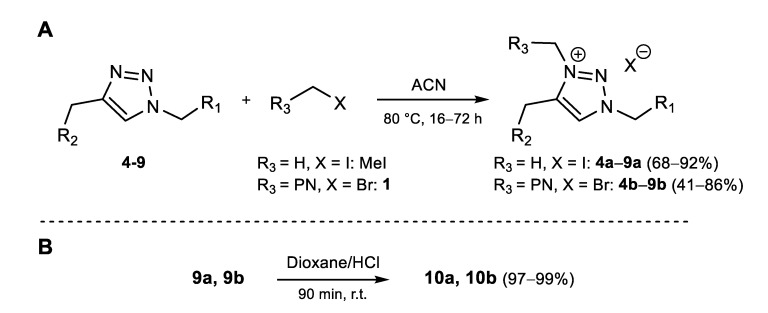
Synthesis of various triazolium salts by (**A**) alkylation with MeI and **1** and (**B**) deprotection of the BOC moiety. Compounds **4a**–**10a** are the methylated triazolium, compounds **4b**–**10b** are the phenalenomethylated triazolium.

Synthesis of the triazolium salts was conducted as described by Fletcher et al. using an alkylating agent in acetonitrile at 80 °C (Scheme 2) [15].

Minimum Inhibitory Concentration (MIC) of the 16 phenalenone derivatives and SAPyR was determined against Gram-positive (*Staphylococcus aureus*, *Staphylococcus epidermidis*, *Bacillus cereus*) and Gram-negative (*Escherichia coli*, *Pseudomonas aeruginosa*) bacteria (Table 2). All the phenalenone derivatives were tested at different concentrations ranging from 0.39 to 200 µM in triplicate in 96-well plates. For the most active compounds, concentrations down to 0.05 µM have been tested. Subsequently, the 96-well plates were irradiated with LED visible light (4.83 mW/cm^2^) for a total fluence of 25 J/cm^2^. Controls consisting of 96-well plates were prepared in the same conditions but kept in the dark.

Due to its low solubility in water, no result could be obtained with **6b** at concentrations higher than 50 µM. All the tested compounds displayed an increased toxicity in presence of light, and some of them were also active in the dark.

Hydrophilic compounds (containing glucose or amine moieties) showed very low effects against all the tested bacterial strains, as compared with more hydrophobic derivatives. No significant difference was observed between neutral **5** and its triazolium salt derivatives **5a** and **5b**, suggesting that hydrophobicity is the critical factor for a better efficacy of these salts. This was confirmed by the increase in cytotoxicity brought by acetylation of the hydroxyl groups of **5** (**4a** and **4b**), or by the addition of hydrophobic substituents, such as adamantane (**6a** and **6b**) or benzyle (**7a** and **7b**).

“Phenalenomethylated” compounds bearing an additional hydrophobic moiety (**6b**, **7b**, **8b**, and **9b**) proved to be the most active derivatives against Gram-positive strains under light as well as dark conditions. This result could be explained by the higher hydrophobicity of the phenalenone moiety compared with the methyl group. The best results were obtained at submicromolar concentrations with **6b**, **7b**, or **8b**. Low MIC values were also obtained in dark conditions, in particular with **6b**, **7b**, and **9b**. The activity against Gram-negative is completely different. Gram-negative strains are much less sensitive to aPDT than Gram-positive strains, mainly due to the presence of an outer layer of lipopolysaccharides lowering the degree of permeability to hydrophobic compounds [23]. No significant activity was detected in the dark against Gram-negative strains, except for **9b** against *P. aeruginosa*, with an MIC value of 25 µM. Under light irradiation, compounds with methylated triazole seem to be more active than “phenalenomethylated” derivatives, which could be explained by the larger size of the latter as well as by their higher hydrophobicity. Compounds **6a** and **10** presented the strongest activity against *E. coli*, and half of the compounds had equal to better activity than SAPyR. Surprisingly, **10** also exhibited an important photodynamic effect against *P. aeruginosa*, while its triazolium derivatives **10a** and **10b** showed very low or no activity.

In summary, we have synthesized and characterized 14 new phenalenone derivatives bearing the triazolium group which have shown medium to good water solubility. Compared with SAPyR, hydrophobic triazolium salt derivatives tested in these specific conditions seem to exhibit a better inhibitory activity against all the tested bacterial strains except *P. aeruginosa*. The next step will be to evaluate whether these new phenalenone derivatives are able to show photobactericidal activity, in particular when exposed to low fluence rates of light. More precisely, we plan to check these compounds for their activity against bacterial biofilms, which represent the real clinical challenge for aPDT.

## 3. Materials and Methods

General methods. *2-(bromomethyl)-1*H*-phenalen-1-one (**1**), 2-(azidomethyl)-1*H*-phenalen-1-one (**2**), 2-((prop-2-yn-1-yloxy)methyl)-1*H*-phenalen-1-one (**3**), (2R,3R,4S,5R,6R)-2-(acetoxymethyl)-6-((1-((1-oxo-1*H*-phenalen-2-yl)methyl)-1*H*-1,2,3-triazol-4-yl)methoxy)tetrahydro-2*H*-pyran-3,4,5-triyl triacetate (**4**), 2-((4-((((2R,3R,4S,5S,6R)-3,4,5-trihydroxy-6-(hydroxymethyl)tetrahydro-2*H*-pyran-2-yl)oxy)methyl)-1H-1,2,3-triazol-1-yl)methyl)-1*H*-phenalen-1-one (**5**), (3r,5r,7r)-N-((1-((1-oxo-1*H*-phenalen-2-yl)methyl)-1*H*-1,2,3-triazol-4-yl)methyl)adamantane-1-carboxamide (**6**), tert-butyl ((1-((1-oxo-1*H*-phenalen-2-yl)methyl)-1*H*-1,2,3-triazol-4-yl)methyl)carbamate (**9**), and (1-oxo-1*H*-phenalen-2-yl)methanaminium chloride (**10**) *were synthetized as described earlier [19]. SAPyR was synthesized following the protocole described by Späth et al. [13]. All other reagents and solvents were purchased from Alfa Aesar, TCI, Carlo Erba, Fisher Chemical, VWR, or Sigma Aldrich and were used as received. The column chromatographies were realized with a silica gel 60 (0.015–0.040 mm) which was purchased from Merck.

Structural characterizations. NMR analyses were conducted on a Bruker DPX 500 NMR spectrometer, operating at 500 MHz and with tetramethylsilane as reference. High-resolution electrospray ionization mass spectra (HR ESI-MS) were performed by the ICOA/CBM (FR2708) at the University of Orléans with a Bruker Q-TOF maXis mass spectrometer coupled to an Ultimate 3000 RSLC chain (Dionex).

Photophysical characterizations. The photophysical characterizations were conducted in water (HPLC grade, supplier = Merck). Both UV-Vis absorption spectra and fluorescence emission spectra were recorded at 21 °C. UV–Vis absorption spectra were measured in quartz cuvettes (1 cm path-length, Hellma) on a Shimadzu UV2700 spectrophotometer. Fluorescence emission spectra were recorded on a SPEX Fluorolog 3 fluorometer. In this fluorometer, double grating monochromators are used in the excitation and emission channels. A Xenon arc lamp (450 W, Osram) is the excitation light source, and a Peltier cooled photomultiplier tube (R636-10, Hamamatsu) is the detector for the fluorescence. For the singlet oxygen emission at 1275 nm, a highly sensitive liquid nitrogen-cooled InGaAs detector (Electro-Optical Systems DSS series cryogenic receiver, 2 mm InGaAs photodiode) was coupled to a Horiba Jobin Yvon Spex Fluorolog 3 spectrofluorometer. The fluorescence signal from the fluorophores in solution is collected in a right-angle geometry, and the fluorescence spectra are corrected for fluctuations of the excitation source flux and for the wavelength dependence of the detection sensitivity. The optical density for photoluminescence spectra was approximately 0.1.

Both quantum yields were determined using the SPEX fluorolog 3 fluorometer relative to a fluorescent standard of known quantum yield. From here, the integrated emission spectra of the unknown sample are compared with that of the standard under the same absorbance conditions (optical density = 0.1) at the same excitation wavelength of 370 nm.

For these measurements, approximately 0.1 mL of the solution was diluted with water. Absorption and emission spectra (both fluorescence and singlet oxygen) were measured using 3.0 mL of the sample and standard solutions in 1 × 1 cm optical path length quartz cells. The quantum yields were calculated using the equation 1 below [24]:

(1) $$\Phi_{x}={\;\Phi }_{r}\left[\frac{A_{r}\left(\lambda_{r}\right)}{A_{x}\left(\lambda_{x}\right)}\right]\left[\frac{I\left(\lambda_{r}\right)}{I\left(\lambda_{x}\right)}\right]\left[\frac{n_{x}{}^{2}}{n_{r}{}^{2}}\right]\left[\frac{D_{x}}{D_{r}}\right]$$

where Φ_x_ = the desired quantum yield (fluorescence or singlet oxygen) of the unknown sample; Φ_r_ = the quantum yield of the known sample according to the literature; A_r_(λ_r_) = absorbance of the reference at the excitation wavelength; A_x_(λ_x_) = absorbance of the unknown sample at the excitation wavelength; I(λ_r_) = relative intensity of the exciting light of a known reference at wavelength λ; I(λ_x_) = relative intensity of the exciting light of an unknown sample at wavelength λ; n_x_ = refractive index of the solvent in which the unknown sample is dissolved; n_r_ = refractive index of the solvent in which the known sample is dissolved; D_x_ = integrated area under the corrected emission spectrum of the unknown sample; D_r_ = integrated area under the corrected emission spectrum of the known sample. n_x_ and n_r_ must be equal for the singlet oxygen determination. The data were analyzed using the Origin program [25].

### 3.1. Synthesis of the Triazolium Salts

**Synthesis of the triazole derivatives.***2-(((1-benzyl-1*H*-1,2,3-triazol-4-yl)methoxy)methyl)-1*H*-phenalen-1-one (**7**).* In total, 91 mg (0.37 mmol) of **3** and 100 µL (0.74 mmol) of benzyl azide were dissolved in 10 mL of CH_2_Cl_2_. 1.3 mg (7 µmol) of CuI, 3 µL (15 µmol) of DIPEA and 1 µL (7 µmol) of acetic acid was added, after which the reaction is stirred at room temperature for 55 h. The solvent was then evaporated, and the crude substance was purified by column chromatography (CH_2_Cl_2_ then CHCl_3_) to give 104 mg (0.27 mmol, 74%) of yellow powder. **^1^H-NMR (500 MHz, CDCl_3_)**: **δ (ppm) =** 8.64 (dd, *J* = 1.0, 7.4 Hz, 1H), 8.21 (dd, *J* = 0.6, 8.0 Hz, 1H), 8.01 (d, *J* = 8.2 Hz, 1H), 7.86 (m, 1H), 7.79 (t, *J* = 8 Hz, 1H), 7.76 (s, 1H), 7.59 (dd, *J* = 7.1, 8.5 Hz, 1H), 7.56 (s, 1H), 7.38–7.27 (m, 5H), 5.54 (s, 2H), 4.82 (s, 2H), 4.66 (d, *J* = 1.2, 2H)—**^13^C-NMR (125 MHz, CDCl_3_)**: **δ (ppm) =** 184.42, 145.44, 138.08, 136.25, 134.95, 134.56, 132.03, 131.53, 131.51, 131.49, 130.42, 129.20, 129.13, 128.91, 128.77, 128.17, 127.89, 127.61, 127.08, 126.80, 122.55, 67.35, 64.62, 54.21.—**MS:** HRMS (ESI^+^), calcd for C_24_H_20_N_3_O_2_ [M + H]^+^: 382.155003, found 382.154573.—**MW**: 381.44 g/mol.—**MP:** 122 °C.

*2-((4-(((1-oxo-1*H*-phenalen-2-yl)methoxy)methyl)-1*H*-1,2,3-triazol-1-yl)methyl)-1*H*-phenalen-1-one (**8**).* In total, 235 mg (1 mmol) of **2** and 248 mg (1 mmol) of **3**, 250 mg (0.2 mmol) of CuSO_4_.5H_2_O, and 198 mg (0.4 mmol) of sodium ascorbate were dissolved in 10 mL of tBuOH/H_2_O 1:1. A precipitate rapidly appears. The reaction was stirred 72 h at room temperature. The solution was poured in 300 mL of water, filtered, and the solid was extracted several times with CH_2_Cl_2_. The organic phase was dried and evaporated. The crude substance was purified on column chromatography (CHCl_3_/MeOH 98:2) to yield 236 mg (0.49 mmol, 49%) of bright yellow powder. **^1^H-NMR (500 MHz, CDCl_3_)**: **δ (ppm) =** 8.67 (dd, *J* = 0.9, 7.4 Hz, 1H), 8.62 (dd, *J* = 0.9, 7.5 Hz, 1H), 8.24 (d, *J* = 7.7 Hz, 1H), 8.19 (d, *J* = 7.8 Hz, 1H), 8.05 (d, *J* = 8.2 Hz, 1H), 7.99 (d, *J* = 8.3 Hz, 1H), 7.94 (s, 1H), 7.87 (t, *J* = 1.0 Hz, 1H), 7.80 (t, *J* = 7.7 Hz, 1H), 7.78–7.74 (m, 3H), 7.74 (s, 1H), 7.61 (dd, *J* = 7.2, 8.1 Hz, 1H), 7.58 (dd, *J* = 7.2, 8.1 Hz, 1H), 5.60 (s, 2H), 4.85 (s, 2H), 4.69 (d, *J* = 1.1 Hz, 2H).—**^13^C-NMR (125 MHz, CDCl_3_)**: **δ (ppm) =** 184.40, 183.89, 145.18, 141.21, 137.93, 136.34, 135.53, 134.90, 133.53, 132.71, 132.65, 132.04, 132.01, 131.51, 131.43, 131.06, 130.38, 129.20, 128.85, 127.65, 127.35, 127.34, 127.07, 127.04, 126.91, 126.84, 126.78, 124.00, 67.30, 64.61, 48.89.—**MS:** HRMS (ESI^+^), calcd for C_31_H_22_N_3_O_3_ [M + H]^+^: 484.165568, found 484.165628.—**MW**: 483.53 g/mol.—**MP:** 125 °C (deg.).

**General procedure for the methylation of the triazoles**. A total of 1 eq. of triazole compound and 20 eq. of methyl iodide were dissolved in the minimum amount of acetonitrile. The reaction was then left at 80 °C for 16 to 24 h. The solvent was evaporated, and the crude substance was purified by column chromatography (CHCl_3_/MeOH 98:2 to 95:5), except for **5a**, and then crystallized in diethyl ether.

*3-methyl-1-((1-oxo-1*H*-phenalen-2-yl)methyl)-4-((((2R,3R,4S,5R,6R)-3,4,5-triacetoxy-6-(acetoxymethyl)tetrahydro-2*H*-pyran-2-yl)oxy)methyl)-1*H*-1,2,3-triazol-3-ium iodide (**4a**).* A total of 350 mg (0.56 mmol) of **4** gave 362 mg (0.47 mmol, 85%) of orange yellow powder. **^1^H-NMR (500 MHz, CDCl_3_)**: **δ (ppm) =** 9.20 (s, 1H), 8.60 (d, J = 7.3 Hz, 1H), 8.49 (s, 1H), 8.26 (d, J = 8.0 Hz, 1H), 8.17 (d, J = 7.0 Hz, 1H), 8.11 (d, J = 8.3 Hz, 1H), 7.80 (t, J = 7.7 Hz, 1H), 7.68 (t, J = 7.7 Hz, 1H), 5.87 (d, J = 14.5 Hz, 1H), 5.83 (d, J = 14.5 Hz, 1H), 5.40 (d, J = 14.4 Hz, 1H), 5.23 (d, J = 14.3 Hz, 1H), 5.22 (t, J = 9.3 Hz, 1H), 5.07 (m, 2H), 4.98 (m, 1H), 4.34 (m, 4H), 4.07 (m, 2H), 2.04 (s, 3H), 2.01 (s, 6H), 1.98 (s, 3H).—**^13^C-NMR (125 MHz, CDCl_3_)**: **δ (ppm) =** 183.45, 170.48, 169.89, 169.63, 169.52, 144.64, 140.52, 136.08, 134.62, 133.68, 132.05, 131.26, 131.24, 129.28, 128.54, 127.58, 127.45, 127.29, 126.25, 100.66, 72.70, 72.13, 71.04, 67.78, 61.07, 60.11, 53.31, 39.39, 20.86, 20.80, 20.60, 20.54.—**MS:** HRMS (ESI^+^), calcd for C_32_H_34_N_3_O_11_ [M]^+^: 636.218785, found 636.218796.—**MW**: 763.54 g/mol.

*3-methyl-1-((1-oxo-1H-phenalen-2-yl)methyl)-4-((((2R,3R,4S,5S,6R)-3,4,5-trihydroxy-6-(hydroxymethyl)tetrahydro-2*H*-pyran-2-yl)oxy)methyl)-1*H*-1,2,3-triazol-3-ium iodide* (**5a**). A total of 90 mg (0.20 mmol) of **5** gave 95 mg (0.16 mmol, 80%) of very hygroscopic yellow powder. The product is stocked in 10 mM aqueous solution at 4 °C until using. **^1^H-NMR (500 MHz, D_2_O)**: **δ (ppm) =** 8.76 (s, 1H), 8.22 (m, 2H), 8.10 (d, J = 8.3 Hz, 1H), 8.00 (s, 1H), 7.84 (d, J = 7.1 Hz, 1H), 7.69 (t, J = 7.7 Hz, 1H), 7.64 (t, J = 7.7 Hz, 1H), 5.63 (s, 2H), 5.21 (d, J = 14.5 Hz, 1H), 5.15 (d, J = 14.5 Hz, 1H), 4.61 (d, J = 7.9 Hz, 1H), 4.36 (s, 3H), 3.76 (dd, J = 1.7, 12.3 Hz, 1H), 3.60 (dd, J = 5.2, 12.3 Hz, 1H), 3.52 (t, J = 9.0 Hz, 1H), 3.40 (m, 2H), 3.36 (dd, J = 8.0, 9.3 Hz, 1H).—**^13^C-NMR (125 MHz, D_2_O)**: **δ (ppm) =** 184.51, 145.76, 140.01, 137.21, 135.23, 134.59, 131.41, 131.32, 130.14, 128.71, 127.41, 127.26, 127.15, 126.01, 125.26, 102.23, 76.03, 75.56, 72.82, 69.33, 60.43, 58.80, 52.78, 38.20.—**MS:** HRMS (ESI^+^), calcd for C_24_H_26_N_3_O_7_ [M + H]^+^: 468.176527, found 468.176445.—**MW**: 595.39 g/mol.

*4-(((1r,3r)-adamantane-1-carboxamido)methyl)-3-methyl-1-((1-oxo-1*H*-phenalen-2-yl)methyl)-1*H*-1,2,3-triazol-3-ium iodide* (**6a**). A total of 41 mg (90 µmol) of **6** gave 38 mg (63 µmol, 70%) of bright yellow powder. **^1^H-NMR (500 MHz, CDCl_3_)**: **δ (ppm) =** 8.97 (s, 1H), 8.59 (d, *J* = 7.3 Hz, 1H), 8.26 (s, 1H), 8.25 (d, *J* = 8 Hz, 1H), 8.11 (m, 3H), 7.80 (t, *J* = 7.7 Hz, 1H), 7.67 (t, *J* = 7.6 Hz, 1H), 5.72 (s, 2H), 4.75 (d, *J* = 5.3 Hz, 2H), 4.43 (s, 3H), 1.99 (s, 3H), 1.87 (s, 6H), 1.68 (s, 6H).—**^13^C-NMR (125 MHz, CDCl_3_)**: **δ (ppm) =** 183.24, 179.80, 144.13, 142.90, 136.06, 134.32, 133.73, 132.02, 131.40, 131.09, 129.37, 128.47, 127.52, 127.51, 127.16, 126.17, 53.23, 40.79, 39.23, 39.06 (3C), 36.37 (3C), 32.28, 28.03 (3C).—**MS:** HRMS (ESI^+^), calcd for C_29_H_31_N_4_O_2_ [M]^+^: 467.244153, found 467.243968.—**MW**: 594.50 g/mol.

*1-benzyl-3-methyl-4-(((1-oxo-1*H*-phenalen-2-yl)methoxy)methyl)-1*H*-1,2,3-triazol-3-ium iodide (**7a**).* A total of 270 mg (0.71 mmol) of **7** gave 339 mg (0.65 mmol, 92%) of yellow powder. **^1^H-NMR (500 MHz, CDCl_3_)**: **δ (ppm) =** 9.55 (s, 1H), 8.56 (d, *J* = 7.2 Hz, 1H), 8.20 (d, *J* = 8.0 Hz, 1H), 8.03 (d, *J* = 7.2 Hz, 1H), 8.01 (s, 1H), 7.97 (d, *J* = 7.0 Hz, 1H), 7.77 (t, *J* = 7.7 Hz, 1H), 7.63 (t, *J* = 7.7 Hz, 1H), 7.59 (m, 2H), 7.40 (m, 3H), 5.92 (s, 2H), 5.08 (s, 2H), 4.64 (s, 2H), 4.39 (s, 3H).—**^13^C-NMR (125 MHz, CDCl_3_)**: **δ (ppm) =** 184.41, 141.69, 140.60, 135.28, 134.00, 132.91, 132.33, 132.01, 131.05, 130.72, 130.56, 130.06, 129.61 (2C), 129.53 (2C), 129.06, 127.39, 127.14, 127.04, 126.99, 69.11, 60.69, 57.60, 39.41.—**MS:** HRMS (ESI^+^), calcd for C_25_H_22_N_3_O_2_ [M]^+^: 396.170653, found 396.170530.—**MW**: 523.37 g/mol.

*3-methyl-4-(((1-oxo-1*H*-phenalen-2-yl)methoxy)methyl)-1-((1-oxo-1*H*-phenalen-2-yl)methyl)-1H-1,2,3-triazol-3-ium iodide* (**8a**). A total of 70 mg (0.14 mmol) of **8** gave 82 mg (0.13 mmol, 91%) of yellow powder. **^1^H-NMR (500 MHz, CDCl_3_)**: **δ (ppm) =** 9.36 (s, 1H), 8.62 (s, 1H), 8.50 (d, *J* = 7.5 Hz, 1H), 8.46 (d, *J* = 7.5 Hz, 1H), 8.16 (m, 3H), 8.03 (d, *J* = 8.2 Hz, 1H), 7.97 (d, *J* = 7.0 Hz, 1H), 7.96 (s, 1H), 7.91 (d, *J* = 7.0 Hz, 1H), 7.71 (t, *J* = 7.8 Hz, 1H), 7.69 (t, *J* = 7.8 Hz, 1H), 7.62 (t, *J* = 7.6 Hz, 1H), 7.58 (t, *J* = 7.6 Hz, 1H), 5.91 (s, 2H), 5.10 (s, 2H), 4.65 (s, 2H), 4.41 (s, 3H).—**^13^C-NMR (125 MHz, CDCl_3_)**: **δ (ppm) =** 184.36, 183.54, 145.14, 141.43, 140.56, 135.92, 135.20, 134.70, 134.07, 133.51, 132.76, 132.26, 131.97, 131.96, 131.11, 130.91, 130.56, 129.16, 129.00, 128.54, 127.54, 127.32, 127.30, 127.26, 127.11, 126.99, 126.97, 126.29, 69.04, 60.76, 53.08, 39.41.—**MS:** HRMS (ESI^+^), calcd for C_32_H_24_N_3_O_3_ [M]^+^: 498.181218, found 498.180972.—**MW**: 625.47 g/mol.

*4-(((tert-butoxycarbonyl)amino)methyl)-3-methyl-1-((1-oxo-1*H*-phenalen-2-yl)methyl)-1*H*-1,2,3-triazol-3-ium iodide (**9a**).* A total of 539 mg (1.38 mmol) of **9** gave 501 mg (0.94 mmol, 68%) of orange yellow powder. **^1^H-NMR (500 MHz, CDCl_3_)**: **δ (ppm) =** 8.94 (s, 1H), 8.60 (d, *J* = 7.3 Hz, 1H), 8.39 (s, 1H), 8.25 (d, *J* = 7.8 Hz, 1H), 8.14 (d, *J* = 6.9 Hz, 1H), 8.11 (d, *J* = 8.2 Hz, 1H), 7.80 (t, *J* = 7.7 Hz, 1H), 7.67 (dd, *J* = 7.4, 8.1 Hz, 1H), 6.40 (t, *J* = 5.4 Hz, 1H), 5.78 (s, 2H), 4.73 (d, *J* = 6.1 Hz, 2H), 4.41 (s, 3H), 1.40 (s, 9H). —**^13^C-NMR (125 MHz, CDCl_3_)**: **δ (ppm) =** 183.35, 156.06, 144.37, 143.07, 136.05, 134.54, 133.67, 132.02, 131.32, 130.87, 129.27, 128.50, 127.55, 127.44, 127.23, 126.21, 80.78, 53.15, 39.29, 34.16, 28.28 (3C).—**MS:** HRMS (ESI^+^), calcd for C_23_H_25_N_4_O_3_ [M]^+^: 405.192117, found 405.192461.—**MW**: 532.38 g/mol.

**General procedure for the “phenalenomethylation” of the triazoles.** A total of 1 eq. of the PN-triazole and 2 eq. of **1** are dissolved in the minimum amount of acetonitrile. The reaction remained at 80 °C for 48 to 72 h. The solvent was evaporated, and the crude substance was purified by column chromatography (CHCl_3_/MeOH 98:2 to 95:5), except for **5b**, then crystallized in diethyl ether.

*1,3-bis((1-oxo-1*H*-phenalen-2-yl)methyl)-4-((((2R,3R,4S,5R,6R)-3,4,5-triacetoxy-6-(acetoxymethyl)tetrahydro-2*H*-pyran-2-yl)oxy)methyl)-1*H*-1,2,3-triazol-3-ium bromide (**4b**).* A total of 350 mg (0.56 mmol) of **4** gave 335 mg (0.37 mmol, 67%) of yellow powder. **^1^H-NMR (500 MHz, CDCl_3_)**: **δ (ppm) =** 9.22 (s, 1H), 8.58 (dd, J = 1.0, 7.3 Hz, 1H), 8.57 (dd, J = 1.0, 7.3 Hz, 1H), 8.51 (s, 1H), 8.31 (s, 1H), 8.28 (d, J = 8.0 Hz, 1H), 8.25 (d, J = 8.0 Hz, 1H), 8.15 (m, 2H), 8.10 (m, 2H), 7.82 (t, J = 7.6 Hz, 1H), 7.79 (t, J = 7.6 Hz, 1H), 7.70 (dd, J = 7.3, 8.0 Hz, 1H), 7.67 (dd, J = 7.3, 8.0 Hz, 1H), 5.88 (s, 2H), 5.83 (s, 2H), 5.50 (d, J = 14.4 Hz, 1H), 5.41 (d, J = 14.4 Hz, 1H), 5.23 (t, J = 9.4 Hz, 1H), 5.05 (m, 3H), 4.21 (m, 1H), 4.01 (m, 2H), 2.02 (s, 3H), 2.00 (s, 3H), 1.97 (s, 3H), 1.92 (s, 3H).—**^13^C-NMR (125 MHz, CDCl_3_)**: **δ (ppm) =** 183.42, 183.33, 170.45, 169.92, 169.64, 169.51, 144.55, 144.13, 141.19, 135.98, 135.90, 134.57, 134.24, 133.50, 133.44, 132.08, 132.02, 131.22, 131.13, 130.92, 129.64, 129.63, 128.60, 128.57, 127.54 (2C), 127.46, 127.33, 127.26, 127.22, 126.42 (2C), 100.76, 72.77, 72.11, 71.08, 67.90, 61.14, 60.40, 53.20, 20.74, 20.69, 20.59, 20.56.—**MS:** HRMS (ESI^+^), calcd for C_45_H_40_N_3_O_12_ [M]^+^: 814.260650, found 814.260689. —**MW**: 894.73 g/mol.

*1,3-bis((1-oxo-1*H*-phenalen-2-yl)methyl)-4-((((2R,3R,4S,5S,6R)-3,4,5-trihydroxy-6-(hydroxymethyl)tetrahydro-2*H*-pyran-2-yl)oxy)methyl)-1*H*-1,2,3-triazol-3-ium bromide (**5b**).* A total of 138 mg (0.30 mmol) of **5** gave 104 mg (0.14 mmol, 48%) of very hygroscopic orange yellow powder. The product is stocked in 10 mM aqueous solution at 4 °C until using. **^1^H-NMR (500 MHz, D_2_O)**: **δ (ppm) =** 9.09 (s, 1H), 8.52 (d, J = 8.2 Hz, 2H), 8.44 (d, J = 7.4 Hz, 2H), 8.34 (d, J = 8.2 Hz, 2H), 8.23 (s, 2H), 8.09 (m, 2H), 7.91 (t, J = 7.7 Hz, 2H), 7.80 (t, J = 7.7 Hz, 1H), 7.79 (t, J = 7.7 Hz, 1H), 5.80 (s, 2H), 5.79 (s, 2H), 5.28 (d, J = 14.1 Hz, 1H), 5.22 (d, J = 14.1 Hz, 1H), 4.44 (d, J = 7.8 Hz, 1H), 3.66 (d, J = 1.4, 11.7 Hz, 1H), 3.42 (dd, J = 6.2, 11.7 Hz, 1H), 3.17 (m, 2H), 3.06 (m, 2H).—**^13^C-NMR (125 MHz, D_2_O)**: **δ (ppm) =** 182.50, 182.48, 143.48, 143.00, 140.87, 136.06 (2C), 133.69, 133.62, 133.33, 133.25, 131.60 (2C), 130.52, 130.40 (2C), 130.35, 130.20, 127.86, 127.85, 127.54, 127.53, 127.30, 127.26, 126.46, 126.40, 125.89, 125.84, 102.78, 77.02, 76.39, 73.09, 69.76, 60.89, 58.55, 52.63, 50.71.—**MS:** HRMS (ESI^+^), calcd for C_37_H_32_N_3_O_8_ [M]^+^: 646.218391, found 646.217763.—**MW**: 726.58 g/mol.

*4-(((3r,5r,7r)-adamantane-1-carboxamido)methyl)-1,3-bis((1-oxo-1*H*-phenalen-2-yl)methyl)-1*H*-1,2,3-triazol-3-ium bromide (**6b**).* A total of 172 mg (0.38 mmol) of **6** gave 113 mg (0.16 mmol, 41%) of bright yellow powder. **^1^H-NMR (500 MHz, CDCl_3_)**: **δ (ppm) =** 8.93 (s, 1H), 8.54 (t, *J* = 7.0 Hz, 1H), 8.54 (t, *J* = 7.0 Hz, 1H), 8.45 (t, *J* = 5.7 Hz, 1H), 8.31 (s, 1H), 8.23 (t, *J* = 8.0 Hz, 1H), 8.23 (t, *J* = 8.0 Hz, 1H), 8.18 (s, 1H), 8.08 (dd, *J* = 7.1, 8.0 Hz, 1H), 8.08 (dd, *J* = 7.1, 8.0 Hz, 1H), 8.04 (d, *J* = 7.0 Hz, 1H), 8.04 (d, *J* = 7.0 Hz, 1H), 7.77 (t, *J* = 7.7 Hz, 1H), 7.76 (t, *J* = 7.7 Hz, 1H), 7.65 (dd, *J* = 7.4, 8.3 Hz, 1H), 7.63 (dd, *J* = 7.4, 8.3 Hz, 1H), 6.03 (s, 2H), 5.72 (s, 2H), 4.96 (d, *J* = 5.8 Hz, 2H), 1.93 (s, 3H), 1.84 (s, 6H), 1.64 (m, 6H).—**^13^C-NMR (125 MHz, CDCl_3_)**: **δ (ppm) =** 183.33, 183.08, 179.70, 143.72, 143.58, 143.53, 135.89, 135.77, 134.20, 134.02, 133.49, 133.28, 131.99, 131.95, 131.24, 131.09, 131.04, 130.13, 129.66, 128.61, 128.43, 127.44, 127.41, 127.38, 127.37, 127.12, 127.10, 126.43, 126.24, 53.06, 51.90, 40.75, 38.90 (3C), 36.38 (3C), 32.37, 28.03 (3C).—**MS:** HRMS (ESI^+^), calcd for C_42_H_37_N_4_O_3_ [M]^+^: 645.286017, found 645.286100.—**MW**: 725.69 g/mol.

*1-benzyl-4-(((1-oxo-1*H*-phenalen-2-yl)methoxy)methyl)-3-((1-oxo-1*H*-phenalen-2-yl)methyl)-1*H*-1,2,3-triazol-3-ium bromide (**7b**).* A total of 49 mg (0.13 mmol) of **7** gave 72 mg (0.11 mmol, 86%) of yellow powder. **^1^H-NMR (500 MHz, CDCl_3_)**: **δ (ppm) =** 9.79 (s, 1H), 8.51 (t, *J* = 8.0 Hz, 2H), 8.17 (d, *J* = 7.0 Hz, 1H), 8.16 (s, 1H), 8.11 (d, *J* = 7.9 Hz, 1H), 8.00 (m, 3H), 7.84 (s, 1H), 7.77 (d, *J* = 7.0 Hz, 1H), 7.73 (t, *J* = 7.7 Hz, 1H), 7.69 (t, *J* = 7.7 Hz, 1H), 7.62 (m, 2H), 7.58 (t, *J* = 7.7 Hz, 1H), 7.58 (t, *J* = 7.7 Hz, 1H), 7.39 (m, 3H), 6.04 (s, 2H), 5.89 (s, 2H), 5.16 (s, 2H), 4.63 (s, 2H).—**^13^C-NMR (125 MHz, CDCl_3_)**: **δ (ppm) =** 184.21, 183.08, 143.18, 141.03, 140.74, 135.71, 135.06, 134.14, 133.94, 133.28, 132.57, 132.03, 131.91, 131.89, 131.40, 131.14, 130.97, 130.47, 129.92, 129.81, 129.61 (2C), 129.47 (2C), 128.98, 128.44, 127.31, 127.30, 127.22, 127.05, 127.02, 127.02, 126.92, 126.25, 68.90, 60.61, 57.70, 51.83.—**MS:** HRMS (ESI^+^), calcd for C_38_H_28_N_3_O_3_ [M]^+^: 574.212518, found 574.212562.—**MW**: 654.56 g/mol.

*4-(((1-oxo-1*H*-phenalen-2-yl)methoxy)methyl)-1,3-bis((1-oxo-1*H*-phenalen-2-yl)methyl)-1*H*-1,2,3-triazol-3-ium bromide (**8b**).* A total of 202 mg (0.42 mmol) of **8** gave 256 mg (0.34 mmol, 81%) of bright yellow powder. **^1^H-NMR (500 MHz, CDCl_3_)**: **δ (ppm) =** 9.45 (s, 1H), 8.73 (s, 1H), 8.54 (dd, *J* = 0.8, 7.5 Hz, 1H), 8.41 (dd, *J* = 0.8, 7.5 Hz, 1H), 8.38 (dd, *J* = 0.8, 7.5 Hz, 1H), 8.20 (d, *J* = 8.1 Hz, 1H), 8.18 (s, 1H), 8.12 (m, 2H), 8.05 (d, *J* = 8.2 Hz, 1H), 8.03 (d, *J* = 7.2 Hz, 1H), 7.98 (d, *J* = 8.1 Hz, 1H), 7.92 (d, *J* = 8.2 Hz, 1H), 7.87 (d, *J* = 8.2 Hz, 1H), 7.74 (t, *J* = 7.7 Hz, 1H), 7.70 (s, 1H), 7.66 (t, *J* = 7.6 Hz, 1H), 7.63 (dd, *J* = 7.4, 8.2 Hz, 1H), 7.62 (m, 1H), 7.57 (t, *J* = 7.7 Hz, 1H), 7.54 (dd, *J* = 7.4, 8.1 Hz, 1H), 7.50 (dd, *J* = 7.4, 8.1 Hz, 1H), 6.06 (s, 2H), 5.92 (s, 2H), 5.18 (s, 2H), 4.61 (s, 2H).—**^13^C-NMR (125 MHz, CDCl_3_)**: **δ (ppm) =** 183.94, 183.65, 183.05, 145.53, 142.86, 140.77, 139.89, 135.77, 135.48, 134.88, 134.64, 134.33, 134.10, 133.33, 133.01, 132.23, 131.97, 131.78, 131.74, 130.99, 130.97, 130.93, 130.34, 130.06, 129.50, 128.80, 128.65, 128.34, 127.55, 127.22 (2C), 127.16, 127.03 (3C), 126.98, 126.97, 126.91, 126.78, 126.48, 126.33, 68.64, 60.84, 53.33, 51.44.—**MS:** HRMS (ESI^+^), calcd for C_45_H_30_N_3_O_4_ [M]^+^: 676.223083, found 676.222808.—**MW**: 756.66 g/mol.

*4-(((tert-butoxycarbonyl)amino)methyl)-1,3-bis((1-oxo-1*H*-phenalen-2-yl)methyl)-1*H*-1,2,3-triazol-3-ium bromide (**9b**).* A total of 460 mg (1.18 mmol) of **9** gave 658 mg (0.99 mmol, 84%) of yellow powder. **^1^H-NMR (500 MHz, CDCl_3_)**: **δ (ppm) =** 8.92 (s, 1H), 8.55 (dd, *J* = 1.1, 7.3 Hz, 1H), 8.54 (d, *J* = 7.3 Hz, 1H), 8.33 (s, 1H), 8.30(s, 1H), 8.24 (dd, *J* = 0.9, 8.1 Hz, 1H), 8.21 (d, *J* = 8.4 Hz, 1H), 8.09 (m, 4H), 7.78 (t, *J* = 7.8 Hz, 1H), 7.76 (t, *J* = 7.8 Hz, 1H), 7.66 (dd, *J* = 7.2, 8.1 Hz, 1H), 7.64 (dd, *J* = 7.2, 8.1 Hz, 1H), 6.84 (s, 1H), 5.97 (s, 2H), 5.79 (s, 2H), 4.89 (d, *J* = 6.0 Hz, 2H), 1.32 (s, 9H).—**^13^C-NMR (125 MHz, CDCl_3_)**: **δ (ppm) =** 183.37, 183.28, 156.17, 144.02, 143.77, 135.88, 135.81, 134.45, 134.16, 133.44, 133.29, 131.98, 131.93, 131.16, 131.13, 130.65, 129.94, 129.56, 128.58, 128.46, 127.43, 127.34, 127.31, 127.17 (2C), 127.16 (2C), 126.44, 126.30, 80.39, 53.08, 51.65, 34.19, 28.22 (3C).—**MS:** HRMS (ESI^+^), calcd for C_36_H_31_N_4_O_4_ [M]^+^: 583.233982, found 583.233983.—**MW**: 663.57 g/mol.

**Deprotection of BOC derivatives.** Hydrolysis of BOC moiety was carried out by dissolving BOC derivatives in dioxane-HCl 37% mixture (10:1 v/v) and stirring the resulting solution for 90 min at room temperature. The solvents were evaporated, and the crude substance was repeatedly dissolved in a minimum amount of methanol, crystallized in diethyl ether and evaporated until obtaining a dry powder.

*4-(ammoniomethyl)-3-methyl-1-((1-oxo-1*H*-phenalen-2-yl)methyl)-1*H*-1,2,3-triazol-3-ium chloride (**10a**)*. A total of 100 mg (0.19 mmol) of **9a** gave 70 mg (0.19 mmol, 99%) of orange yellow powder. **^1^H-NMR (500 MHz, DMSO-d_6_)**: **δ (ppm) =** 9.13 (s, 1H), 9.06 (s, 2H), 8.56 (d, *J* = 8.4 Hz, 1H), 8,55 (d, *J* = 7.3 Hz, 1H), 8.37 (d, *J* = 8.3 Hz, 1H), 8.35 (s, 1H), 8.22 (d, *J* = 7.0 Hz, 1H), 7.97 (t, *J* = 7.7 Hz, 1H), 7.83 (t, *J* = 7.7 Hz, 1H), 5.91 (s, 2H), 4.42 (s, 2H), 4,41 (s, 3H).—**^13^C-NMR (125 MHz, DMSO-d_6_)**: **δ (ppm) =** 182.46, 143.13, 137.47, 136.25, 133.96, 133.45, 131.72 (2C), 130.56 (2C), 127.93, 127.69, 127.44, 126.52, 125.95, 52.41, 38.72, 30.60.—**MS:** HRMS (ESI^+^), calcd for C_18_H_17_N_4_O [M − H]^+^: 305.139688, found 305.139596.—**MW**: 377.27 g/mol.

*4-(ammoniomethyl)-1,3-bis((1-oxo-1*H*-phenalen-2-yl)methyl)-1*H*-1,2,3-triazol-3-ium chloride (**10b**).* A total of 144 mg (0.22 mmol) of **9b** gave 117 mg (0.21 mmol, 97%) of orange yellow powder. **^1^H-NMR (500 MHz, D_2_O)**: **δ (ppm) =** 9.11 (s, 1H), 7.88 (d, *J* = 4.7 Hz, 1H), 7.73 (m, 1H), 7.63 (m, 1H), 7.59 (m, 1H), 7.57 (m, 1H), 7.53 (m, 1H), 7.52 (m, 1H), 7.46 (m, 1H), 7.37 (m, 1H), 7.20 (m, 1H), 7.13 (m, 2H), 7.06 (m, 2H), 5.63 (s, 2H), 5.54 (s, 2H), 4.96 (s, 2H).—**^13^C-NMR (125 MHz, D_2_O)**: **δ (ppm) =** 186.79, 186.49, 148.63, 147.91, 139.46, 139.20, 139.12, 137.42, 137.09, 136.81, 136.56, 134.15, 133.50, 133.12 (2C), 133.02, 131.42, 131.05, 129.40, 129.30, 129.23, 129.07, 129.01, 128.83, 127.91, 127.65, 127.20, 127.00, 56.29, 54.22, 34.11.—**MS:** HRMS (ESI^+^), calcd for C_31_H_23_N_4_O_2_ [M − H]^+^: 483.181552, found 483.181547.—**MW**: 555.13 g/mol.

### 3.2. Microbiological Assays

**Bacterial strains and conditions of culture.** Two Gram-positive (*Staphylococcus aureus* CIP76.25 and *Staphylococcus epidermidis* CIP109.562) and three Gram-negative (*Pseudomonas aeruginosa* CIP76110; *Escherichia coli* CIP53.126 and *Escherichia coli* CIP54.8T) bacterial strains were obtained from the Collection Institut Pasteur (CIP, Institut Pasteur Paris, France). *Bacillus cereus* CH was obtained from Anyang Yuanshou Biopharmaceutical (China). These strains were cultured in liquid Tryptic Soy Broth (pancreatic casein extract 17 g/L, soy flour papaic digest 3 g/L, dextrose 2.5 g/L, NaCl 5 g/L, and K_2_HPO_4_ 2.5 g/L) and incubated overnight at 37 °C under aerobic conditions.

**Minimum Inhibitory Concentration (MIC) assays**. Inoculum were prepared following a standard microdilution assay procedure using tryptic soy broth (TSB) and performed in triplicate. All the compounds were dissolved in a water/ethanol 99:1 mixture to obtain stock solutions at a concentration of 1 mM and kept at 4 °C. Then, fresh solutions of each compound at 400 µM in TSB were prepared. A total of 200 µL of these solutions were added in 96-well plates, and a 1:1 serial dilution was performed from 400 µM to 1.56 µM. A total of 100 µL of a bacterial suspension at 2.10^6^ CFU/mL was added to each well, which resulted in a range of concentrations from 200 to 0.78 µM. Subsequently, the 96-well plates were irradiated with white LED visible light (wavelength around: 390–700 nm, 4.83 mW/cm^2^) for a total fluence of 25 J/cm^2^. Controls consisting of 96-well plates were prepared in the same conditions but kept in the dark. After irradiation, the 96-well plates were incubated overnight at 37 °C under aerobic conditions. The lowest concentration of each compound that prevented bacterial growth was considered the Minimum Inhibitory Concentration (MIC) of each compound. All compounds displaying activity at the minimum 0.78 µM concentration of the original assay were tested again with 25 µM as the initial concentration instead of 200 µM and using the same procedure previously described. A total of three independent experiments were performed with each strain.

## Figures and Tables

**Table 1:** Structure of the triazolium salts, yield of alkylation or deprotection, partition coefficients (log P) calculated at pH = 7.3, and singlet oxygen quantum yields (ΦΔ) in H_2_O based on 1*H*-phenalene-1-one-2-sulfonic acid (PNS) [22] with Φ∆ = 0.97 as a reference. For all chemical structures see Figure S49 in Supplementary Materials.

Compound	R_1_	R_2_	R_3_	X	Yield (%)	Log P	Φ_Δ_
**4a**	PN	Glc (OAc)_4_	H	I	85	−2.0	0.80
**4b**	PN	Glc (OAc)_4_	PN	Br	67	+0.4	>0.98
**5a**	PN	Glc	H	I	80	−3.7	0.97
5b *	PN	Glc	PN	Br	48	−1.3	0.31
**6a**	PN	NHCOAda	H	I	70	+0.7	0.70
**6b**	PN	NHCOAda	PN	Br	41	+3.1	0.59
**7a**	Ph	OCH_2_PN	H	I	92	+0.7	>0.99
**7b**	Ph	OCH_2_PN	PN	Br	86	+3.2	N/A
**8a**	PN	OCH_2_PN	H	I	91	+1.4	>0.99
**8b**	PN	OCH_2_PN	PN	Br	81	+3.8	N/A
**9a**	PN	NHBOC	H	I	68	−0.4	0.93
**9b**	PN	NHBOC	PN	Br	84	+2.0	0.23
**10a**	PN	NH_3_^+^	H	2Cl	99	−1.8	0.87
**10b**	PN	NH_3_^+^	PN	2Cl	97	+0.7	N/A
**5**	PN	Glc				−0.7	0.87
**10**	PN	NH_3_^+^				+0.6	0.91
**SAPYR**						−0.5 (Litt. −1.3) [13]	0.83

* Compound obtained with a purity of 70%, mixed with 30% of compound **5**. N/A: signal too weak.

**Table 2:** Minimum Inhibitory Concentration of 17 phenalenone derivatives tested against 6 bacterial strains (E.c. 1: *Escherichia coli* CIP54.8T, E.c. 2: *Escherichia coli* CIP53.126, S.a.: *Staphylococcus aureus* CIP76.25, S.e.: *Staphylococcus epidermidis* CIP109.562, B.c.: *Bacillus cereus* CH, P.a.: *Pseudomonas aeruginosa* CIP76.110) under dark (D) and light (L) conditions.

Compound	Minimum Inhibitory Concentration (µM)											
	*E.c. 1*		*E.c. 2*		*P.a.*		*S.a.*		*S.e.*		*B.c.*	
	D	L	D	L	D	L	D	L	D	L	D	L
**4a**	>200	100	>200	100	>200	200	>200	100	>200	200	>200	200
**4b**	>200	>200	>200	100	200	200	100	12.5	100	12.5	100	50
**5**	>200	>200	>200	>200	>200	>200	>200	100	>200	>200	>200	>200
**5a**	>200	>200	>200	>200	>200	>200	>200	>200	>200	200	>200	>200
5b *	>200	>200	>200	>200	>200	200	>200	200	>200	200	>200	200
**6a**	>200	**50**	>200	**25**	>200	200	50	6.25	200	12.5	>200	100
**6b**	*ND*	*ND*	*ND*	ND	*ND*	*ND*	25	3.12	6.25	**0.78**	25	6.25
**7a**	>200	200	>200	100	>200	>200	50	6.25	25	6.25	100	50
**7b**	>200	>200	>200	>200	>200	>200	6.25	0.78	6.25	**0.78**	12.5	**3.12**
**8a**	>200	100	>200	50	>200	100	25	0.78	12.5	1.56	25	12.5
**8b**	>200	200	>200	100	>200	200	50	**0.39**	12.5	**0.78**	50	6.25
**9a**	>200	100	>200	50	>200	>200	>200	25	>200	12.5	>200	200
**9b**	>200	>200	>200	>200	25	**25**	6.25	1.56	12.5	1.56	6.25	6.25
**10**	>200	100	>200	50	>200	12.5	>200	50	>200	100	>200	>200
**10a**	>200	>200	>200	>200	>200	200	>200	200	>200	100	>200	>200
**10b**	>200	>200	>200	>200	>200	>200	100	50	>200	12.5	>200	>200
**SAPyR**	>200	200	>200	>200	>200	50	>200	50	200	25	>200	50

* Compound obtained with a purity of 70%, mixed with 30% of compound **5**. ND: Not data, compounds not soluble at the concentrations.

## Data Availability

No datastets were generated.

## Supplementary Materials

The following are available online at https://www.mdpi.com/article/10.3390/antibiotics10060626/s1: Table S1. Molar extinction coefficient (ε), fluorescence quantum yield (Φf), and singlet oxygen quantum yield (ΦΔ) of the tested PN in water. Figure S1 to S48: ^1^H and ^13^C NMR spectra, HRMS and ESI+ spectra. Figure S49: chemical strcuture of all compounds.
