# Supplementary material for: Development of Phenalenone-Triazolium Salt Derivatives for aPDT: Synthesis and Antibacterial Screening
Source: Antibiotics (Basel). 2021 May 24;10(6):626. doi: 10.3390/antibiotics10060626 (PMC8225023; doi:10.3390/antibiotics10060626)

Supplementary Materials

Development of phenalenone-triazolium salts derivatives for aPDT: synthesis and antibacterial screening.

Jérémy Godard [1], Dáire Gibbons [1,2], Stéphanie Leroy-Lhez[1], René M. Williams[2], Nicolas Villandier[1], Tan-Sothea Ouk [1], Frédérique Brégier [1] and Vincent Sol [1*]

1 *Université de Limoges, PEIRENE, EA 7500, 123 Avenue Albert Thomas, 87060 Limoges Cedex, France.*
2 *Molecular Photonics Group, Van't Hoff Institute for Molecular Sciences (HIMS),Universiteit van Amsterdam, Science Park 904, 1098 XH Amsterdam, The Netherlands*
* Correspondence: vincent.sol@unilim.fr

Table S1. Molar extinction coefficient (ε), fluorescence quantum yield (Φ_f), and singlet oxygen quantum yield (Φ_Δ) of the tested PN in water 3

Figure S1. ^{1}H NMR of compound **7** in $CDCl_3$ 4
Figure S2. ^{13}C NMR of compound **7** in $CDCl_3$ 4
Figure S3. HRMS ESI^+ of compound **7** 5
Figure S4. ^{1}H NMR of compound **8** in $CDCl_3$ 6
Figure S5. ^{13}C NMR of compound **8** in $CDCl_3$ 6
Figure S6. HRMS ESI^+ of compound **8** 7
Figure S7. ^{1}H NMR of compound **4a** in $CDCl_3$ 8
Figure S8. ^{13}C NMR of compound **4a** in $CDCl_3$ 8
Figure S9. HRMS ESI^+ of compound **4a** 9
Figure S10. ^{1}H NMR of compound **4b** in $CDCl_3$ 10
Figure S11. ^{13}C NMR of compound **4b** in $CDCl_3$ 10
Figure S12. HRMS ESI^+ of compound **4b** 11
Figure S13. ^{1}H NMR of compound **5a** in D_2O 12
Figure S14. ^{13}C NMR of compound **5a** in D_2O 12
Figure S15. HRMS ESI^+ of compound **5a** 13
Figure S16. ^{1}H NMR of compound **5b** in D_2O 14
Figure S17. ^{13}C NMR of compound **5b** in D_2O (*contamination by compound **5**) 14
Figure S18. HRMS ESI^+ of compound **5b** 15
Figure S19. ^{1}H NMR of compound **6a** in $CDCl_3$ 16
Figure S20. ^{13}C NMR of compound **6a** in $CDCl_3$ 16
Figure S21. HRMS ESI^+ of compound **6a** 17
Figure S22. ^{1}H NMR of compound **6b** in $CDCl_3$ 18
Figure S23. ^{13}C NMR of compound **6b** in $CDCl_3$ 18
Figure S24. HRMS ESI^+ of compound **6b** 19
Figure S25. ^{1}H NMR of compound **7a** in $CDCl_3$ 20
Figure S26. ^{13}C NMR of compound **7a** in $CDCl_3$ 20
Figure S27. HRMS ESI^+ of compound **7a** 21
Figure S28. ^{1}H NMR of compound **7b** in $CDCl_3$ 22
Figure S29. ^{13}C NMR of compound **7b** in $CDCl_3$ 22
Figure S30. HRMS ESI^+ of compound **7b** 23

Figure S31. ^{1}H NMR of compound **8a** in $CDCl_3$ 24
Figure S32. ^{13}C NMR of compound **8a** in $CDCl_3$ 24
Figure S33. HRMS ESI^+ of compound **8a** 25
Figure S34. ^{1}H NMR of compound **8b** in $CDCl_3$ 26
Figure S35. ^{13}C NMR of compound **8b** in $CDCl_3$ 26
Figure S36. HRMS ESI^+ of compound **8b** 27
Figure S37. ^{1}H NMR of compound **9a** in $CDCl_3$ 28
Figure S38. ^{13}C NMR of compound **9a** in $CDCl_3$ 28
Figure S39. HRMS ESI^+ of compound **9a** 29
Figure S40. ^{1}H NMR of compound **9b** in $CDCl_3$ 30
Figure S41. ^{13}C NMR of compound **9b** in $CDCl_3$ 30
Figure S42. HRMS ESI^+ of compound **9b** 31
Figure S43. ^{1}H NMR of compound **10a** in DMSO-d_6 32
Figure S44. ^{13}C NMR of compound **10a** in DMSO-d_6 32
Figure S45. HRMS ESI^+ of compound **10a** 33
Figure S46. ^{1}H NMR of compound **10b** in D_2O 34
Figure S47. ^{13}C NMR of compound **10b** in D_2O 34
Figure S48. HRMS ESI^+ of compound **10b** 35
Figure S49. Chemical structure of all compounds 36

Table S1. Molar extinction coefficient (ε), fluorescence quantum yield (Φ_f), and singlet oxygen quantum yield (Φ_Δ) of the tested PN in water

**Compound**	**ε ($L.mol^{-1}.cm^{-1}$)**	**Φ_f**	**Φ_Δ**
**PNS**	9026.88	<0.01	0.97
**4a**	3538.81	0.02	0.80
**4b**	6824.01	0.01	>0.98
**5a**	8074.44	0.02	0.97
**5b**	7265.28	<0.01	0.31
**6a**	7818.8	<0.01	0.70
**6b**	4009.84	<0.01	0.59
**7a**	3493.81	<0.01	>0.99
**7b**	4421.33	<0.01	N/A – signal not good
**8a**	4189.9	<0.01	>0.99
**8b**	5932.82	<0.01	N/A – signal not good
**9a**	2417.86	0.02	0.93
**9b**	3590.89	0.01	0.23
**10a**	3243.01	0.02	0.87
**10b**	6414.40	0.02	N/A – signal not good
**5**	10396.61	0.02	0.87
**10**	2790.03	0.02	0.91
**SAPyr**	2834.47	0.02	0.83

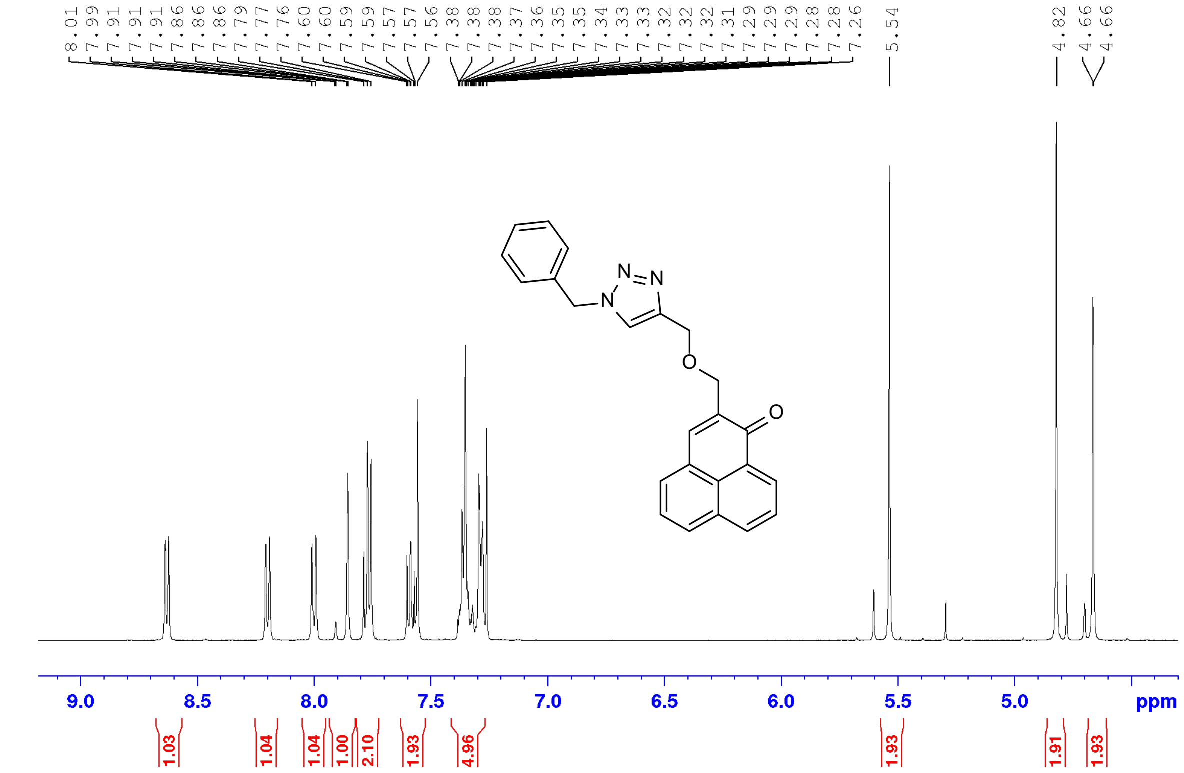

Figure S1. ^{1}H NMR of compound **7** in $CDCl_3$

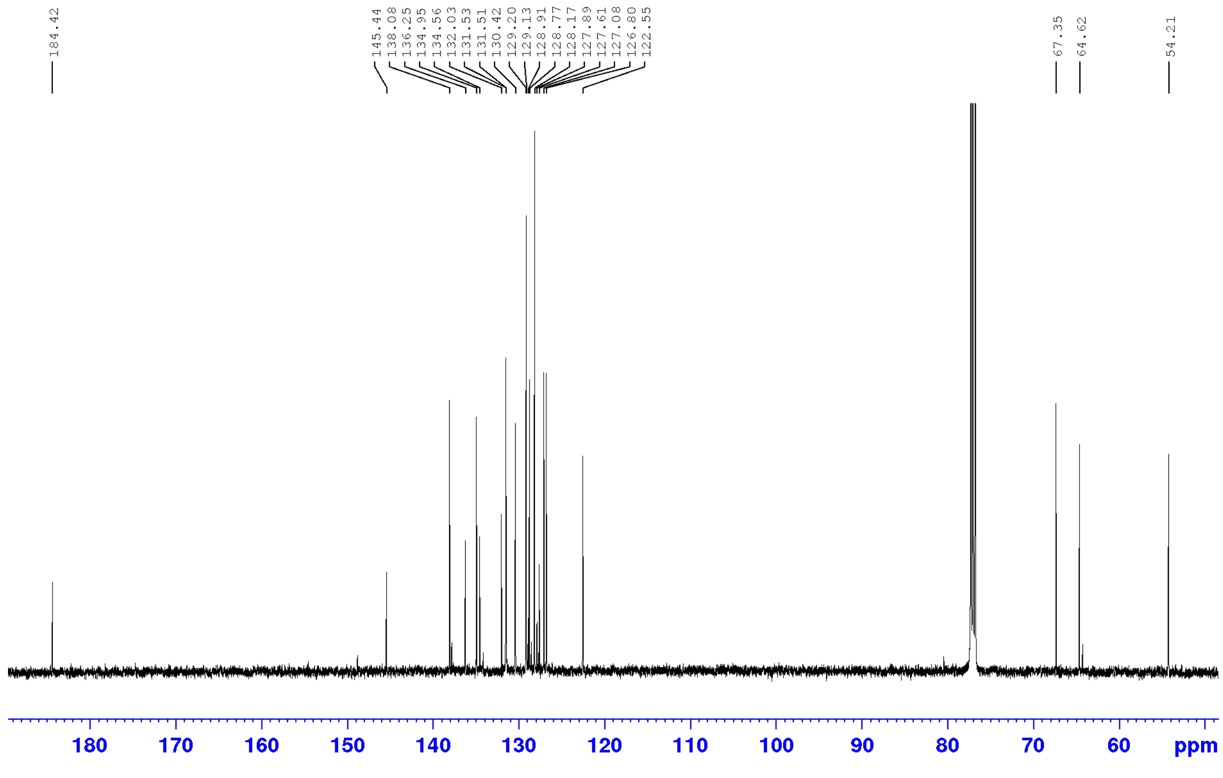

Figure S2. ^{13}C NMR of compound **7** in $CDCl_3$

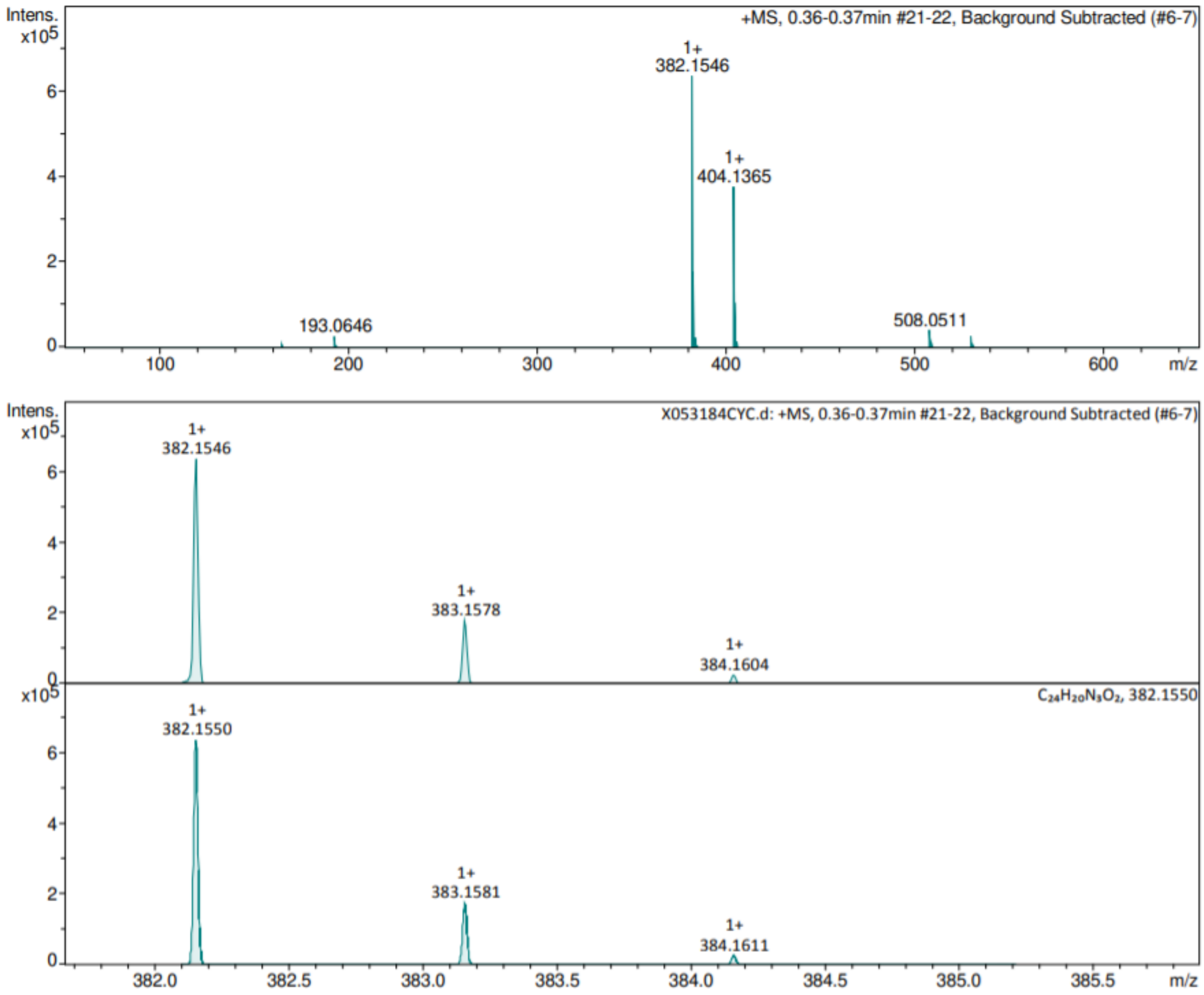

Figure S3. HRMS ESI+ of compound **7**

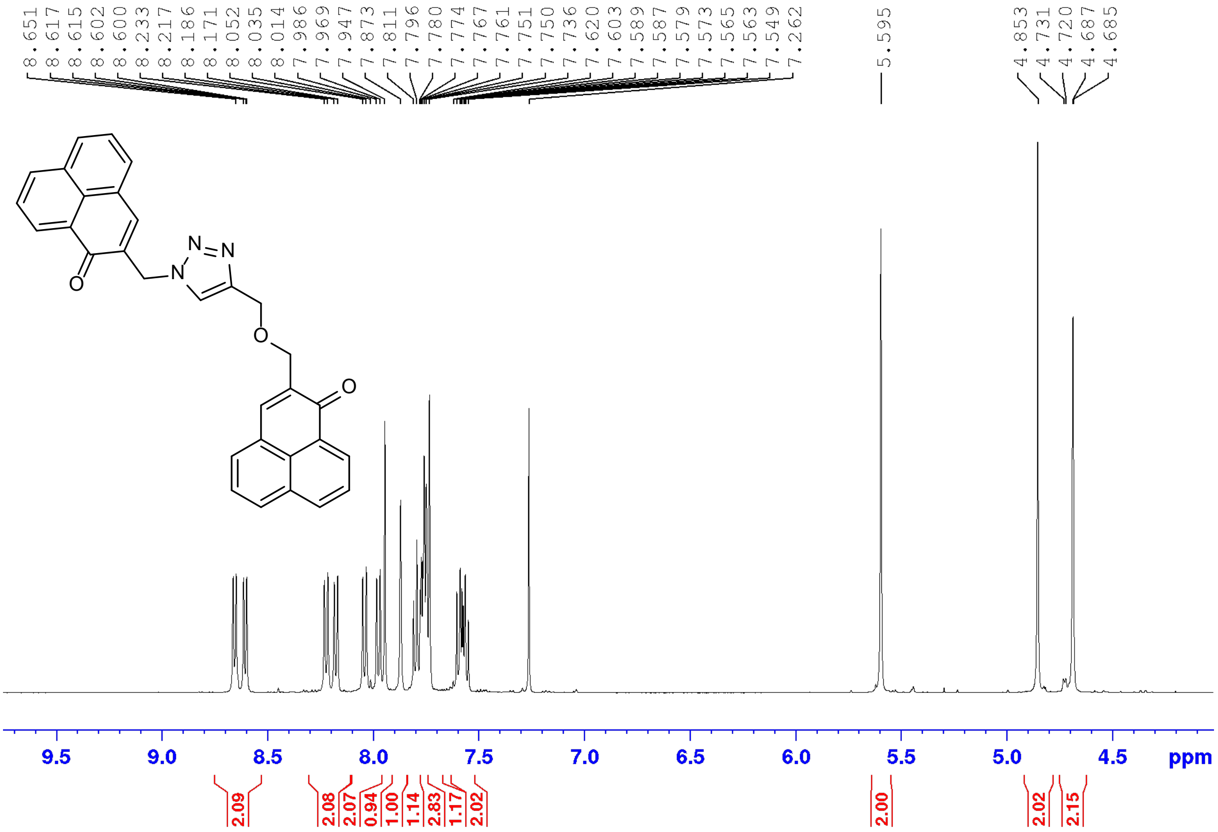

Figure S4. ^{1}H NMR of compound **8** in $CDCl_3$

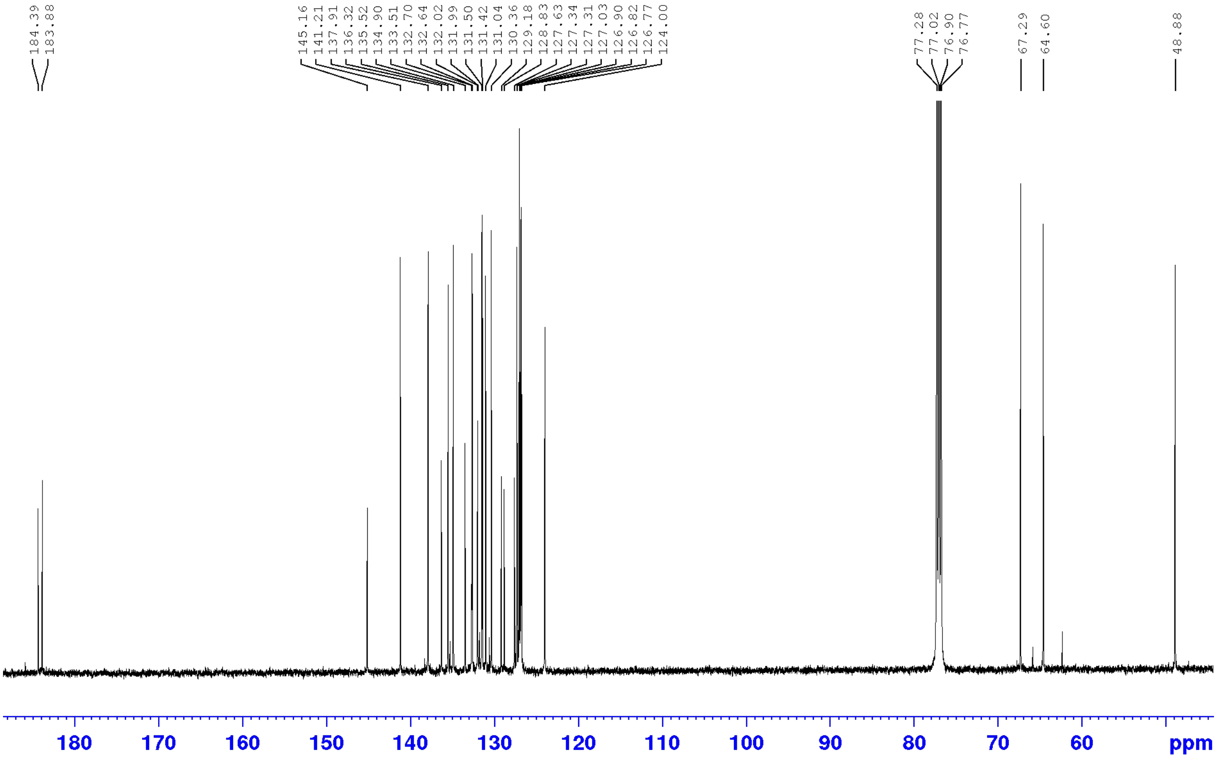

Figure S5. ^{13}C NMR of compound **8** in $CDCl_3$

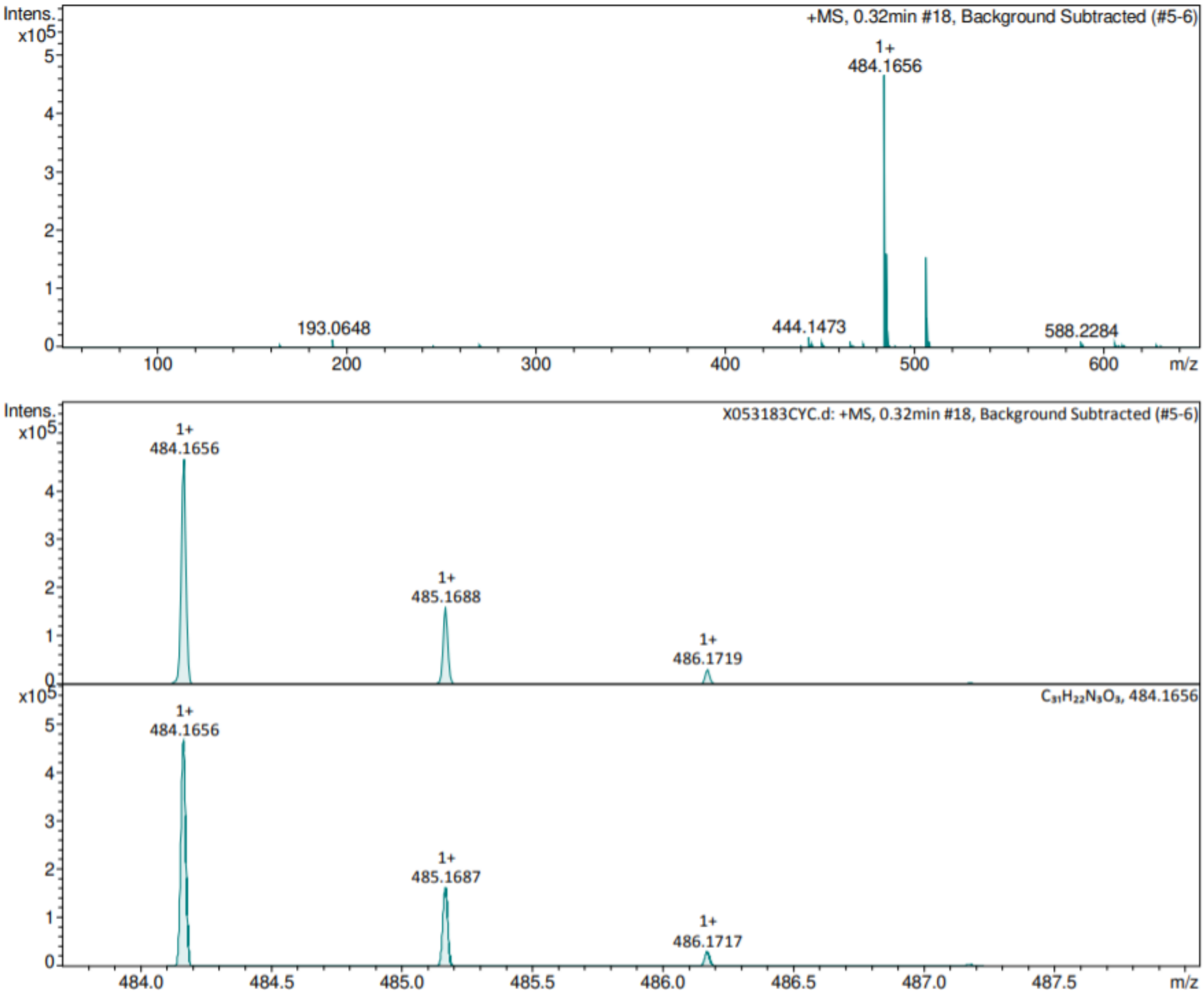

Figure S6. HRMS ESI+ of compound **8**

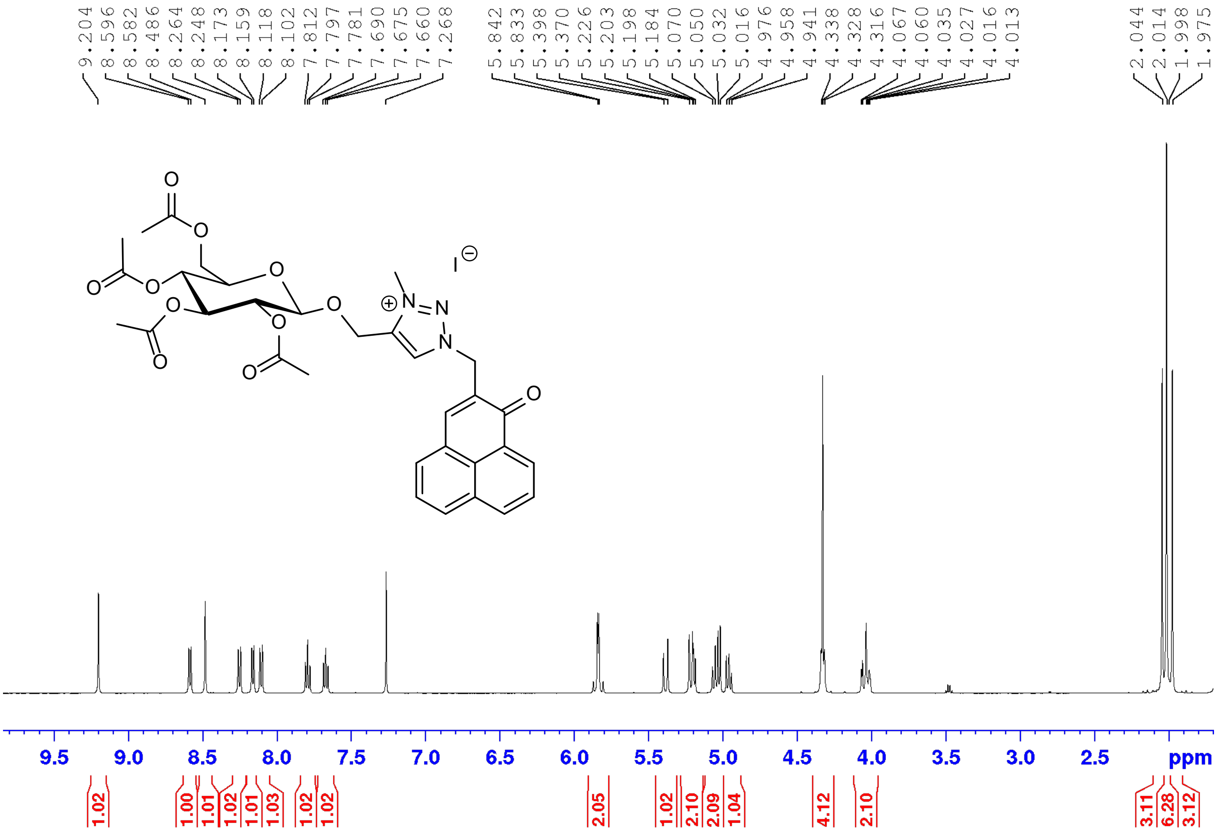

Figure S7. ^{1}H NMR of compound **4a** in $CDCl_3$

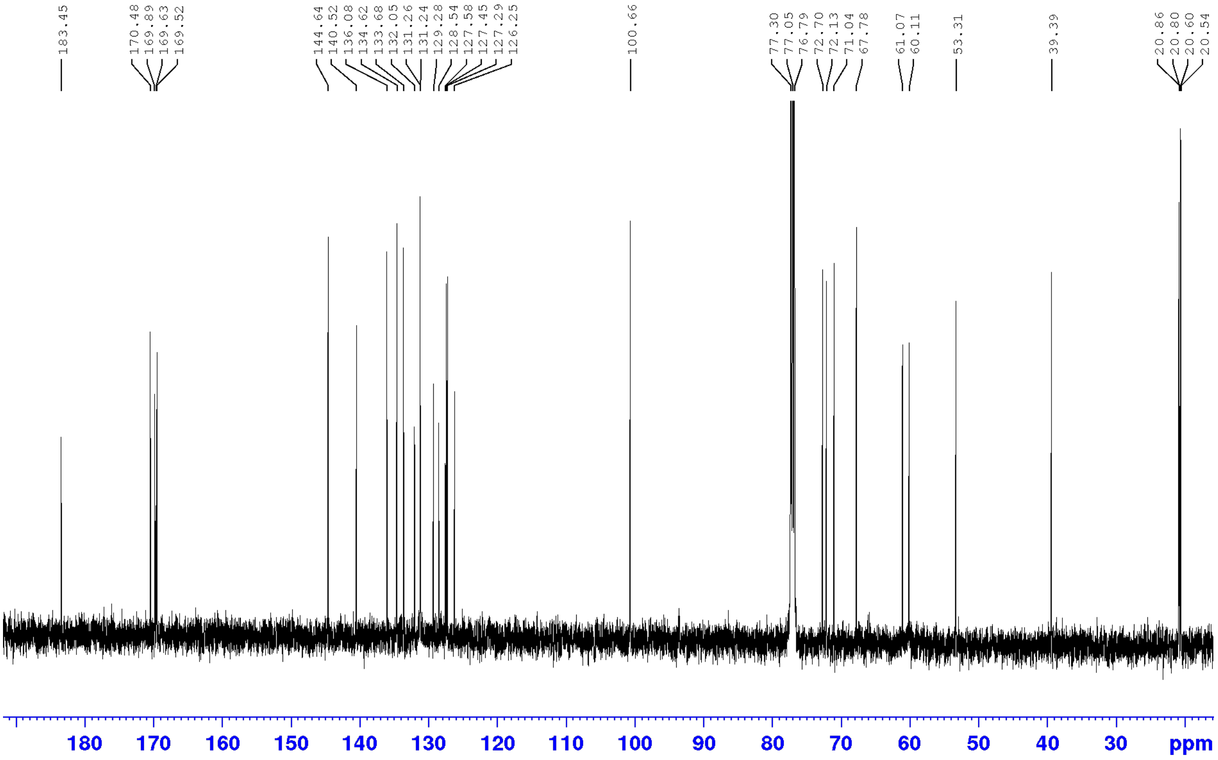

Figure S8. ^{13}C NMR of compound **4a** in $CDCl_3$

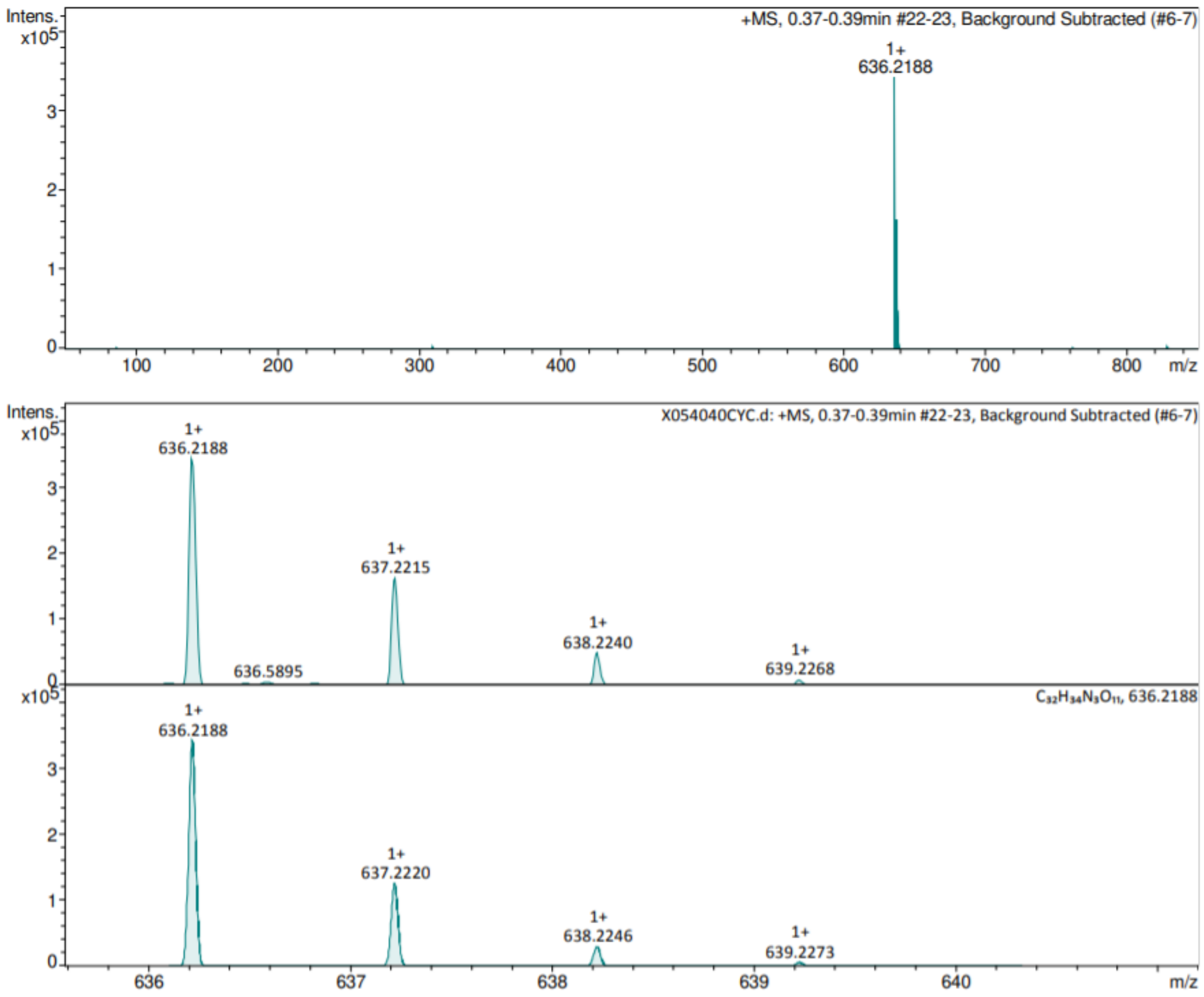

Figure S9. HRMS ESI+ of compound **4a**

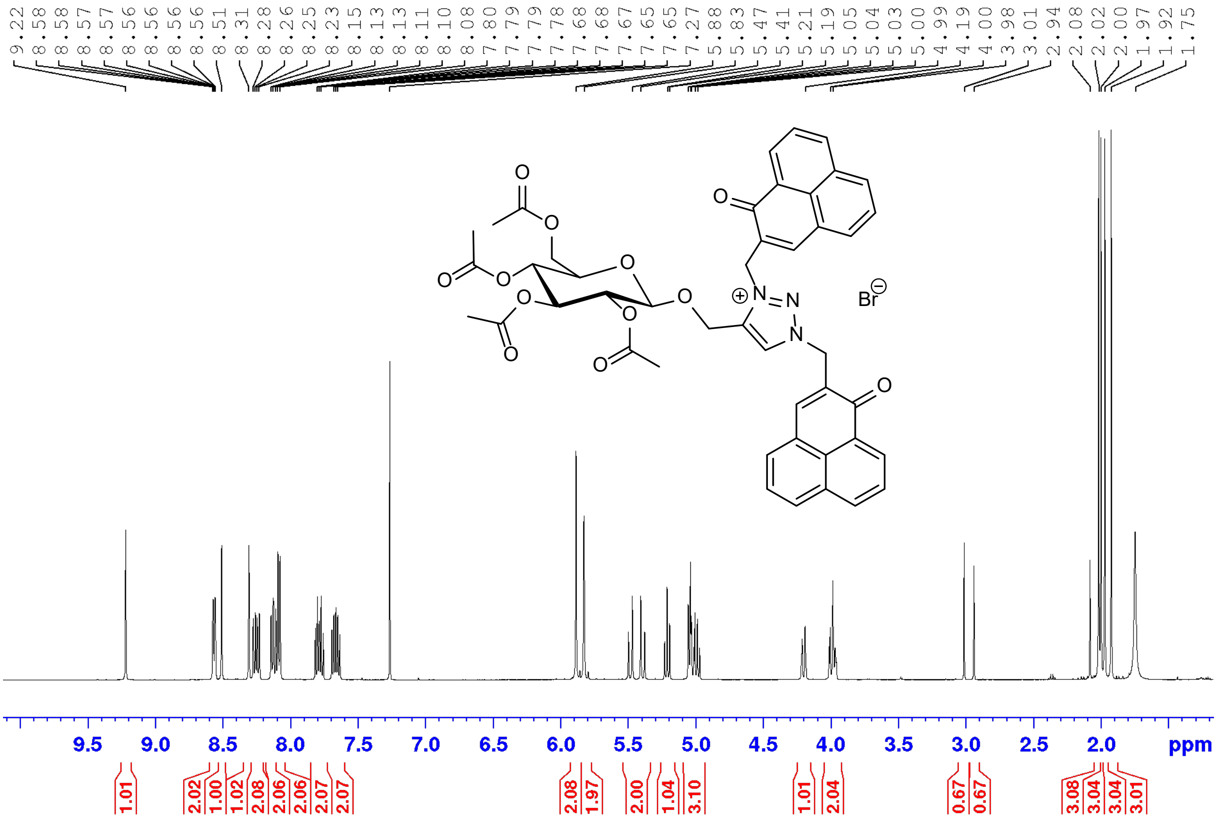

Figure S10. ^{1}H NMR of compound **4b** in $CDCl_3$

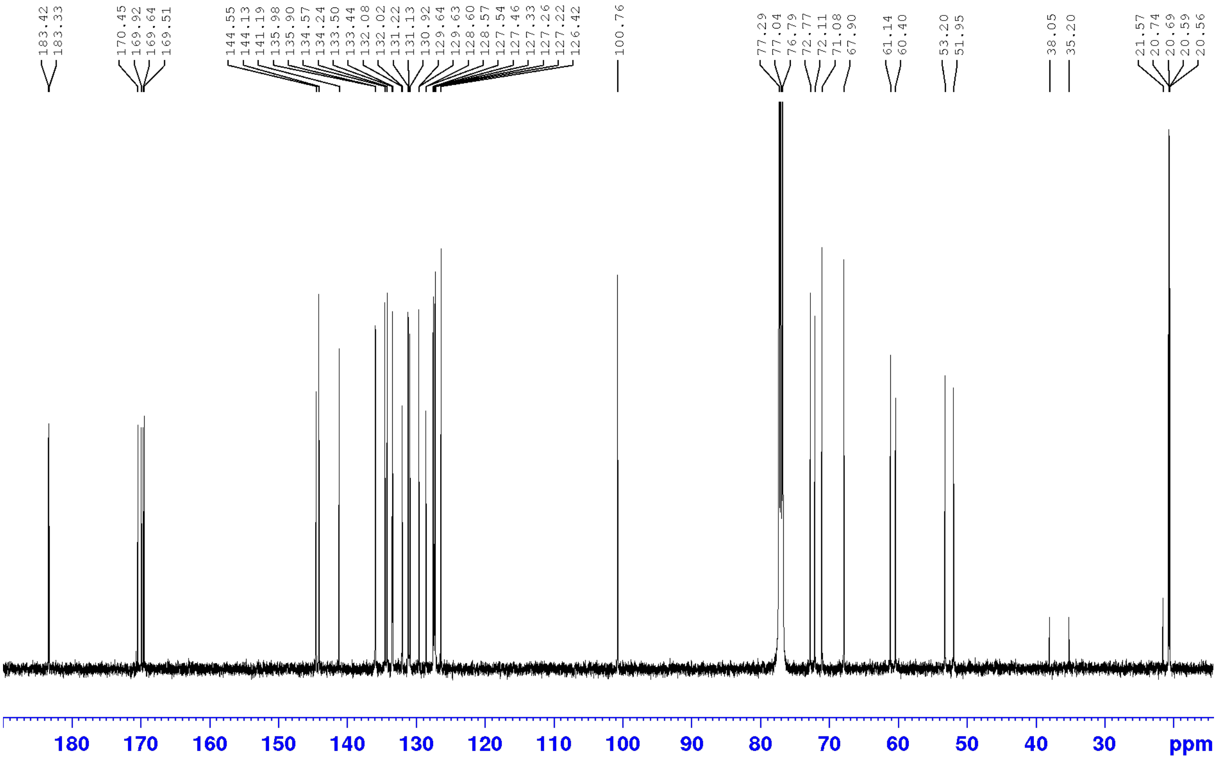

Figure S11. ^{13}C NMR of compound **4b** in $CDCl_3$

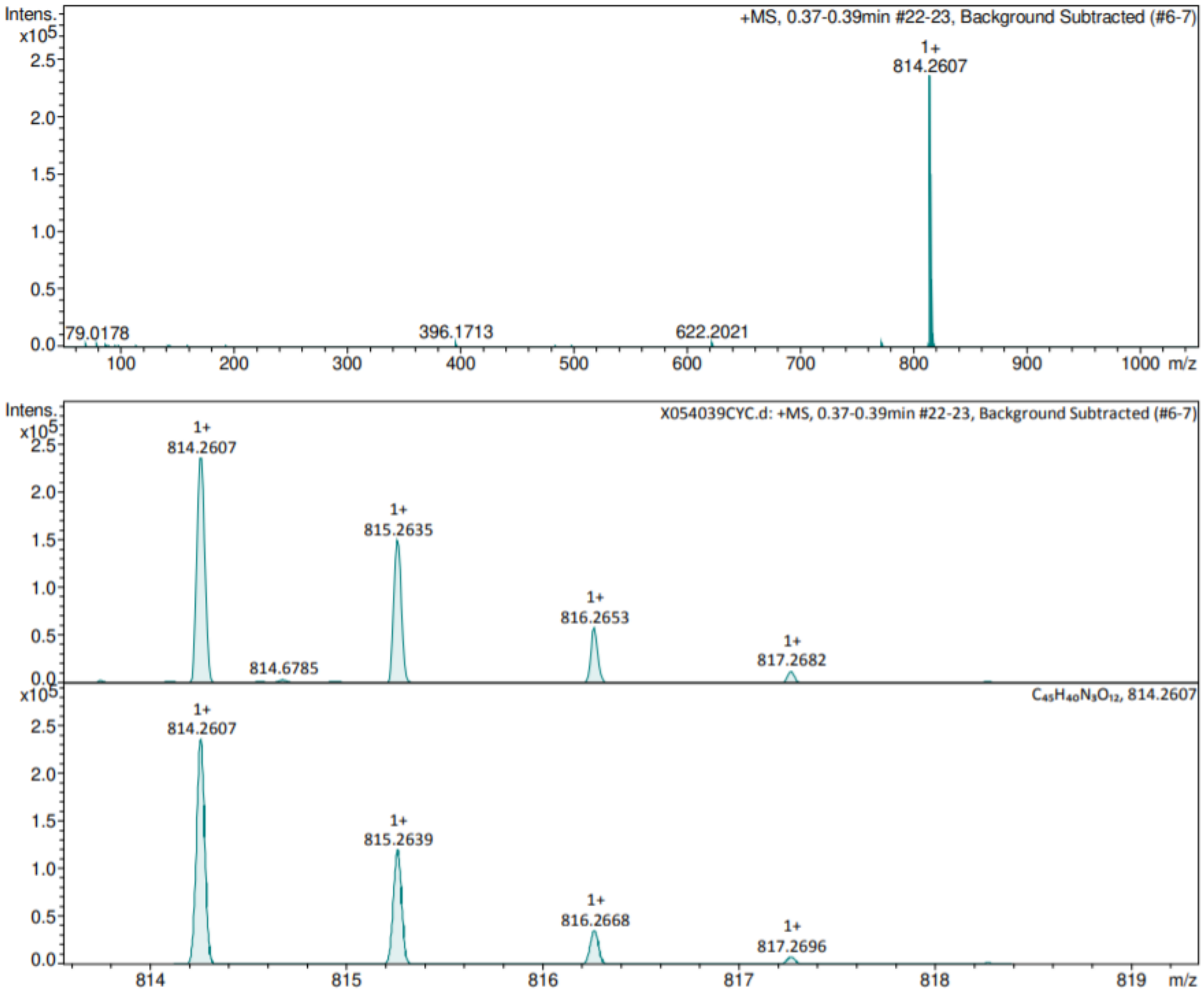

Figure S12. HRMS ESI+ of compound **4b**

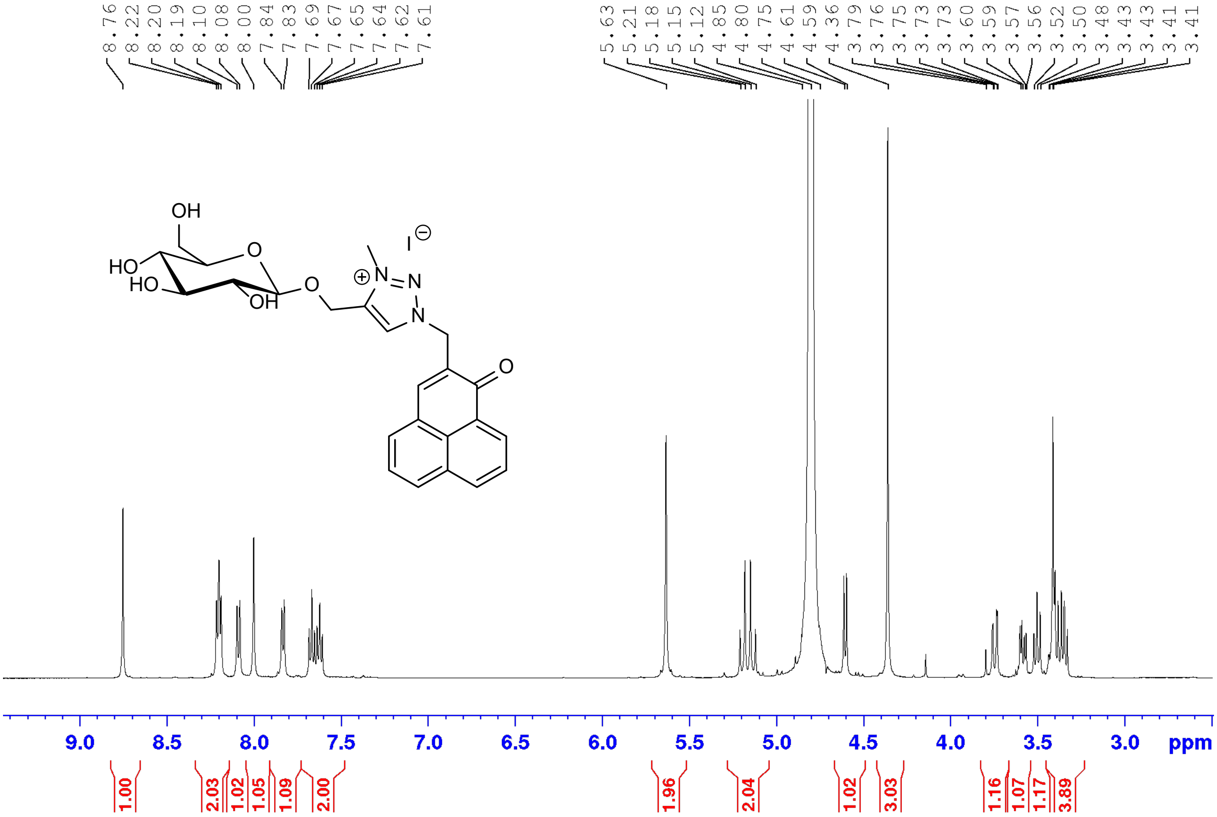

Figure S13. ^{1}H NMR of compound **5a** in D_2O

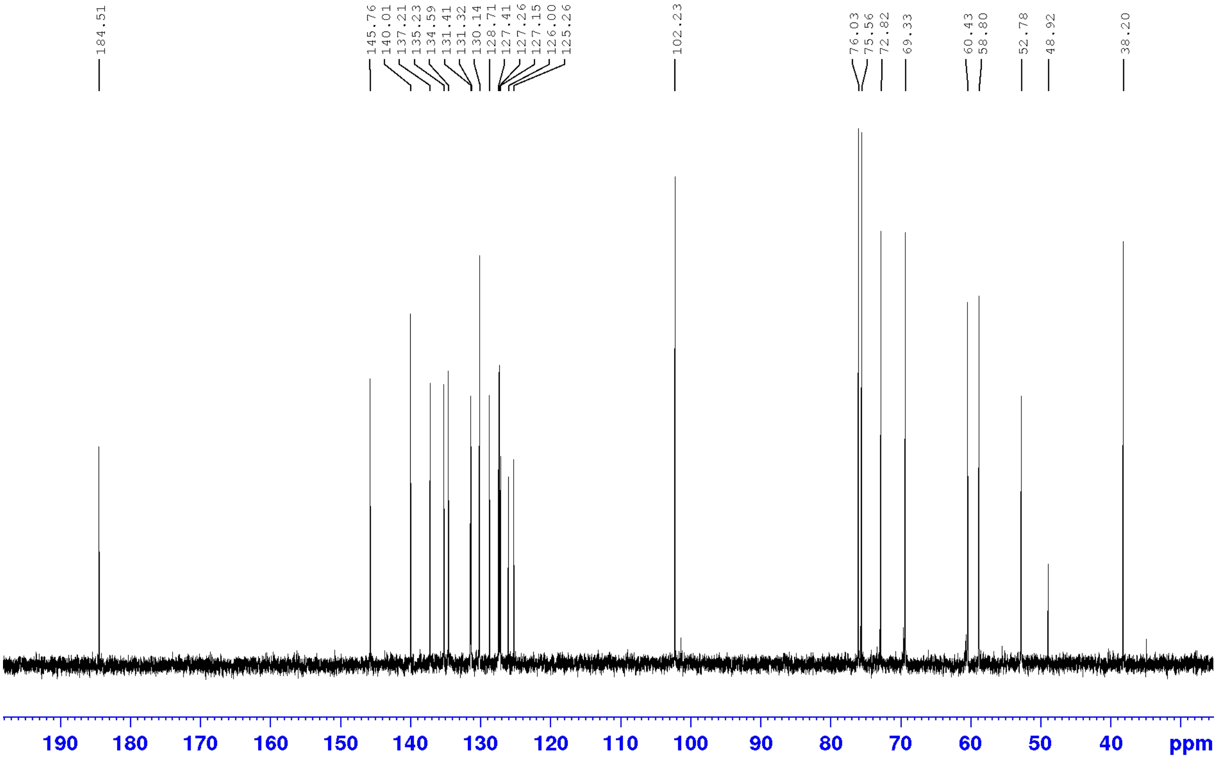

Figure S14. ^{13}C NMR of compound **5a** in D_2O

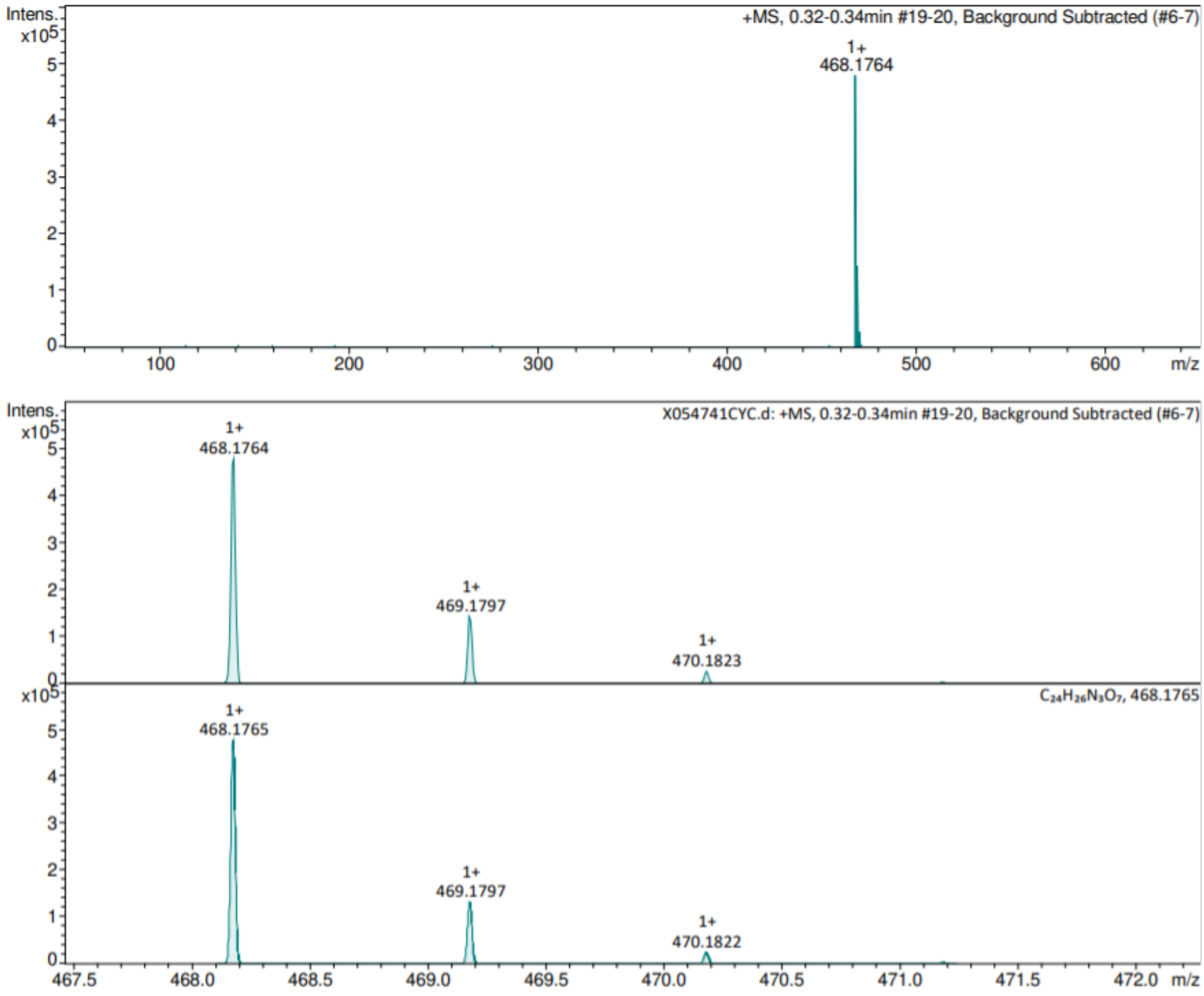

Figure S15. HRMS ESI+ of compound **5a**

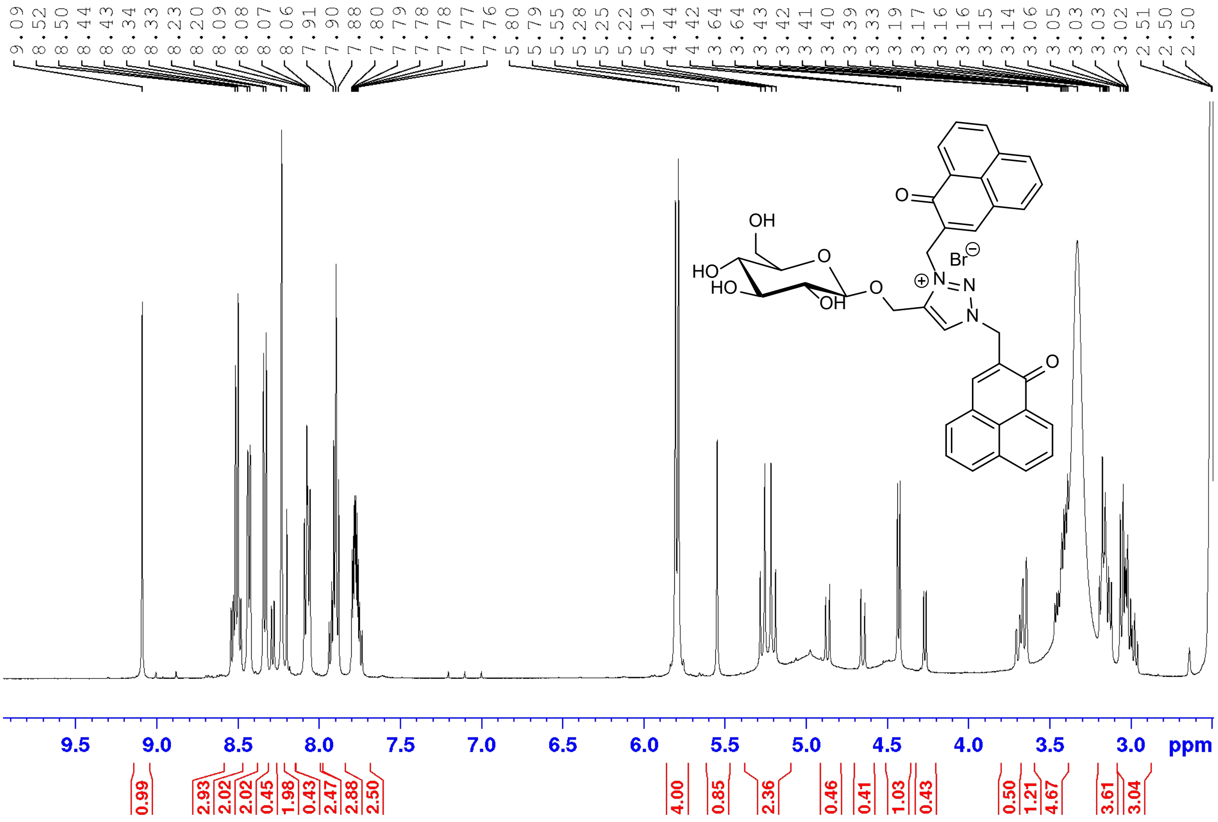

Figure S16. ^{1}H NMR of compound **5b** in D_2O

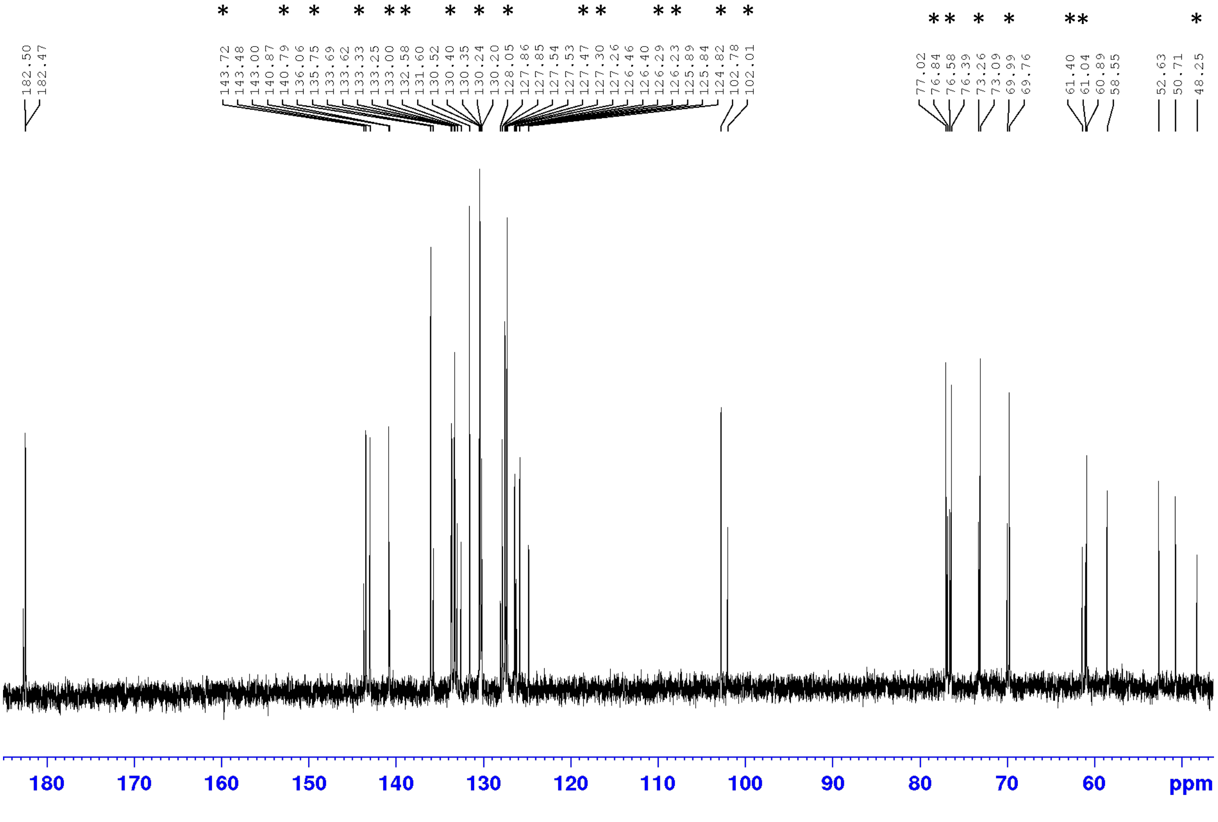

Figure S17. ^{13}C NMR of compound **5b** in D_2O (*contamination by compound **5**)

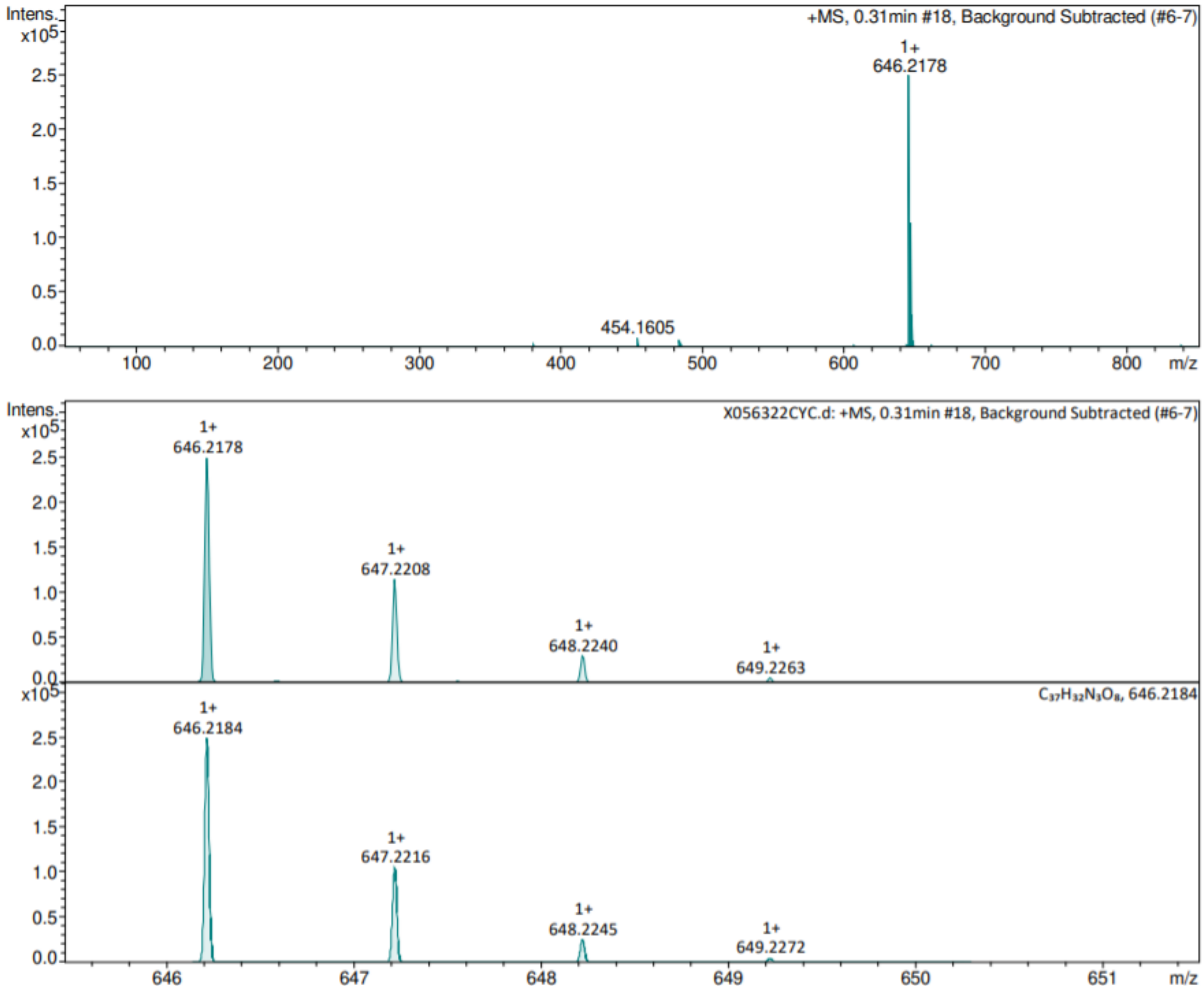

Figure S18. HRMS ESI+ of compound **5b**

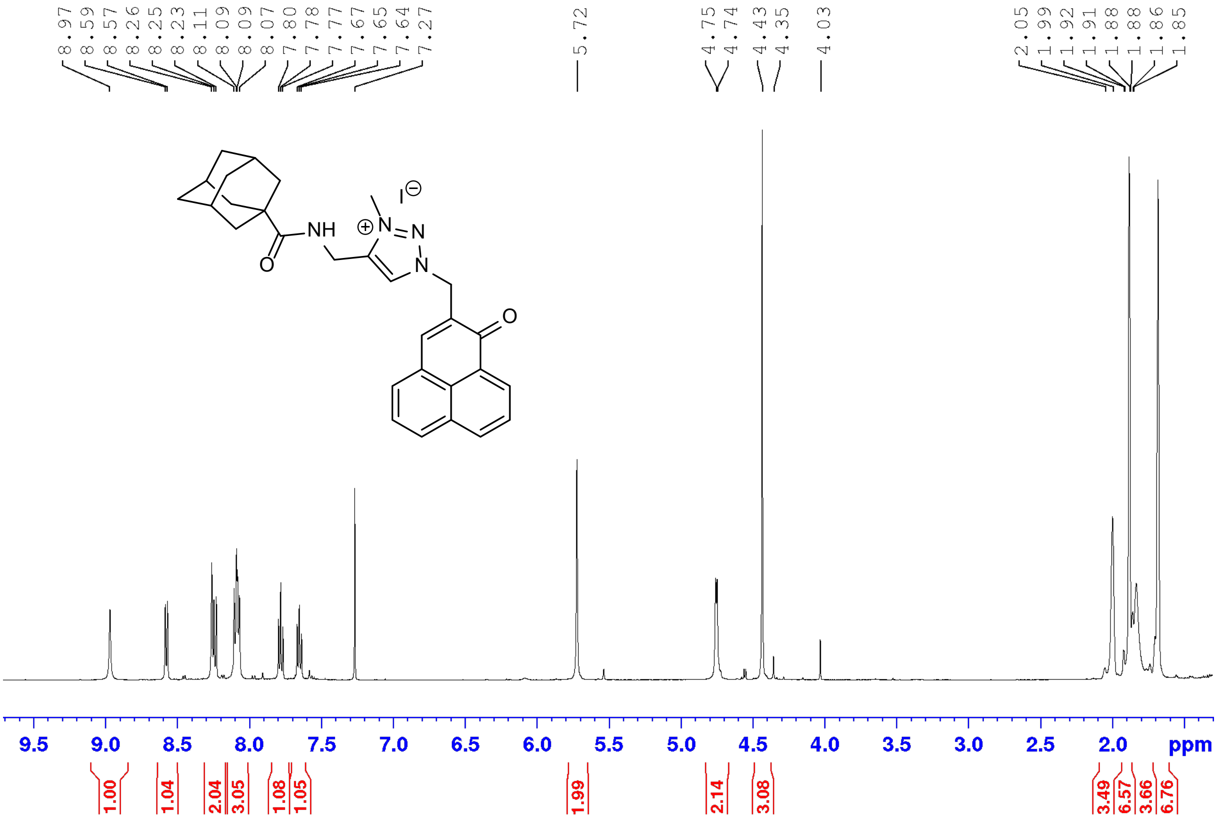

Figure S19. ^{1}H NMR of compound **6a** in $CDCl_3$

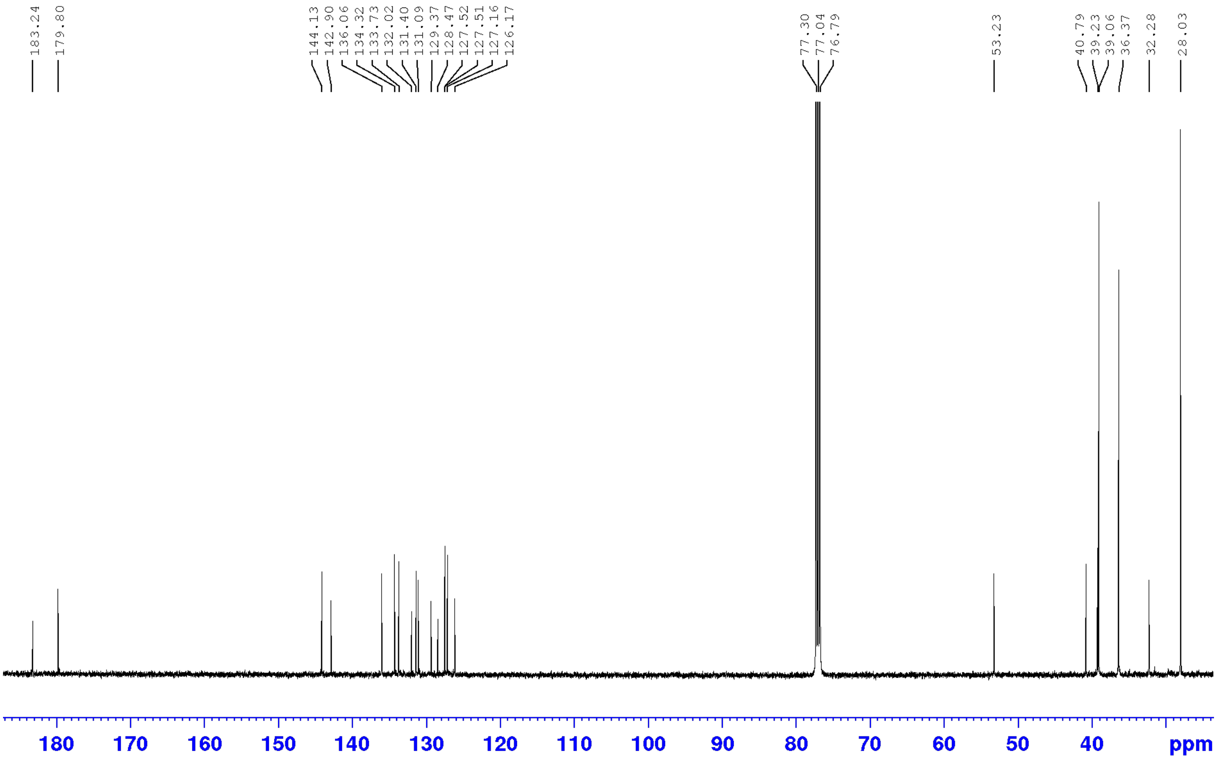

Figure S20. ^{13}C NMR of compound **6a** in $CDCl_3$

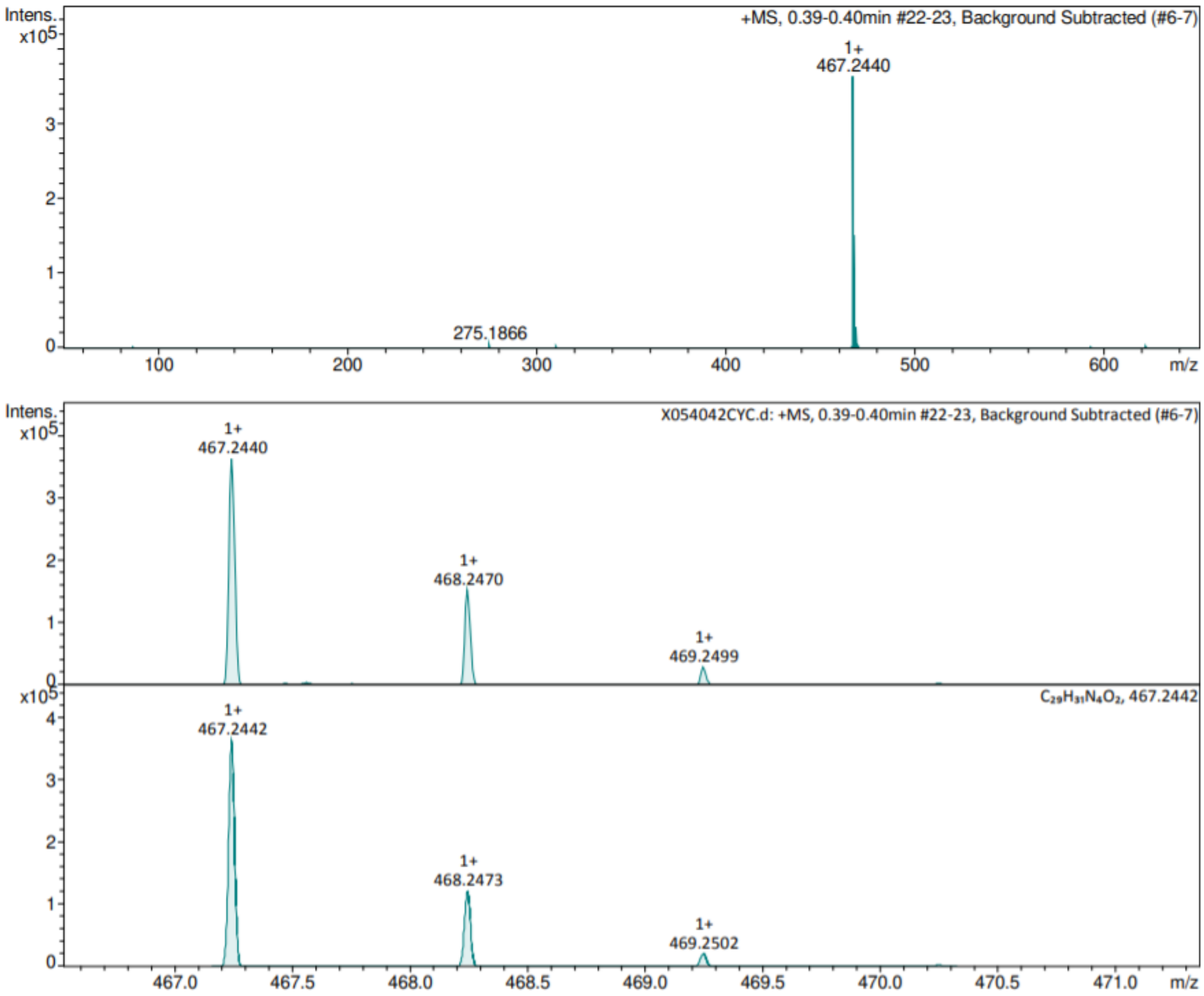

Figure S21. HRMS ESI+ of compound **6a**

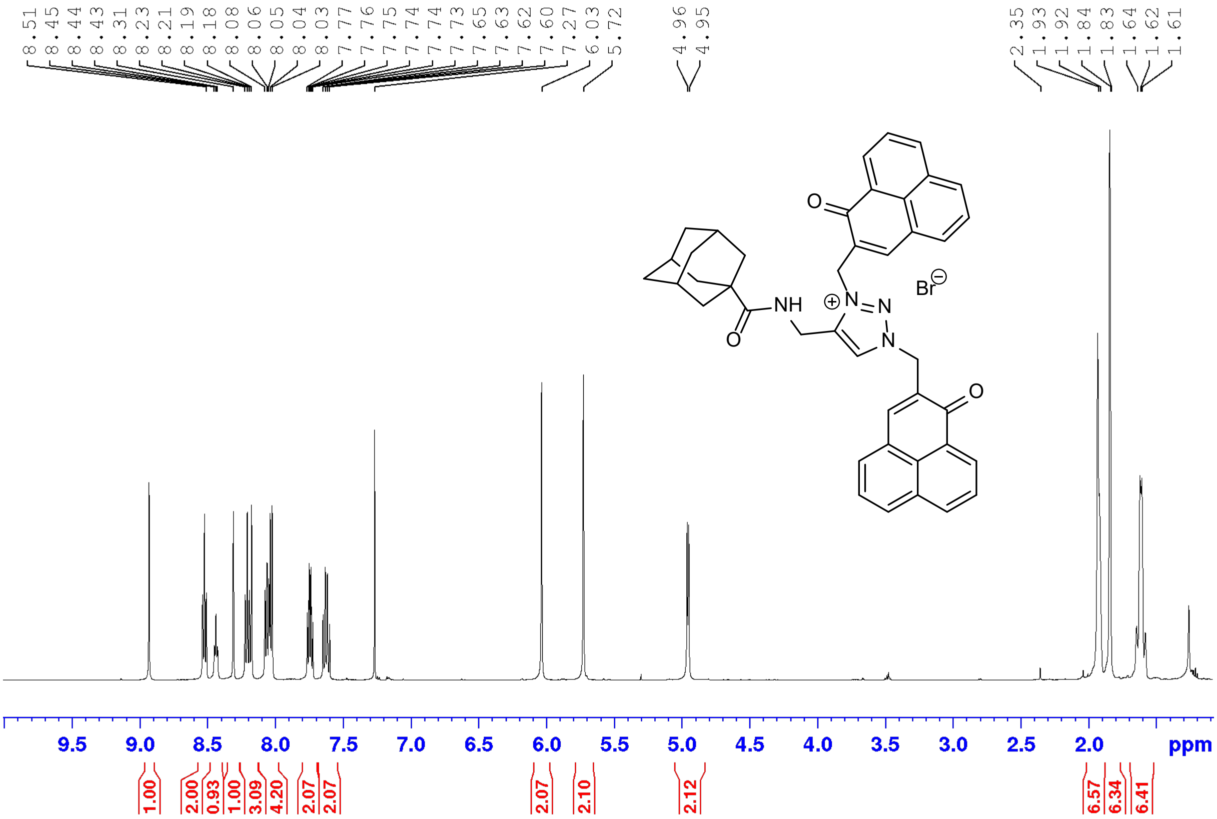

Figure S22. ^{1}H NMR of compound **6b** in $CDCl_3$

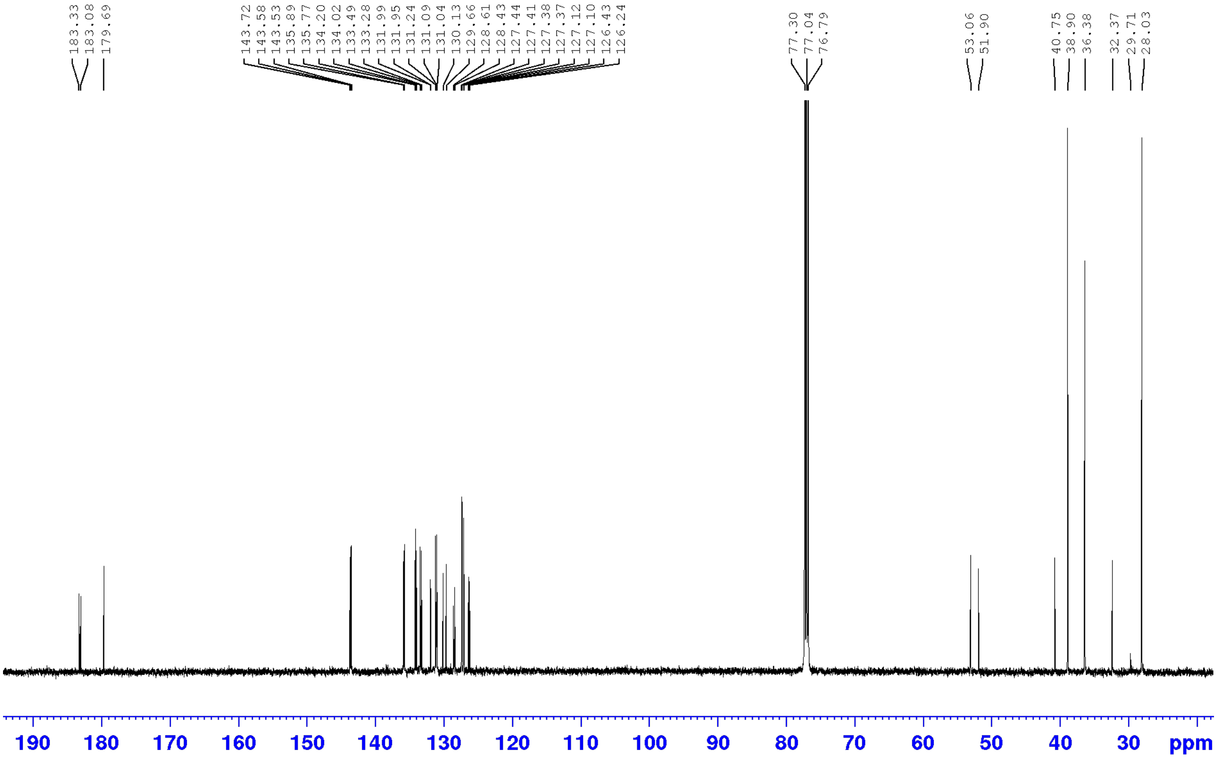

Figure S23. ^{13}C NMR of compound **6b** in $CDCl_3$

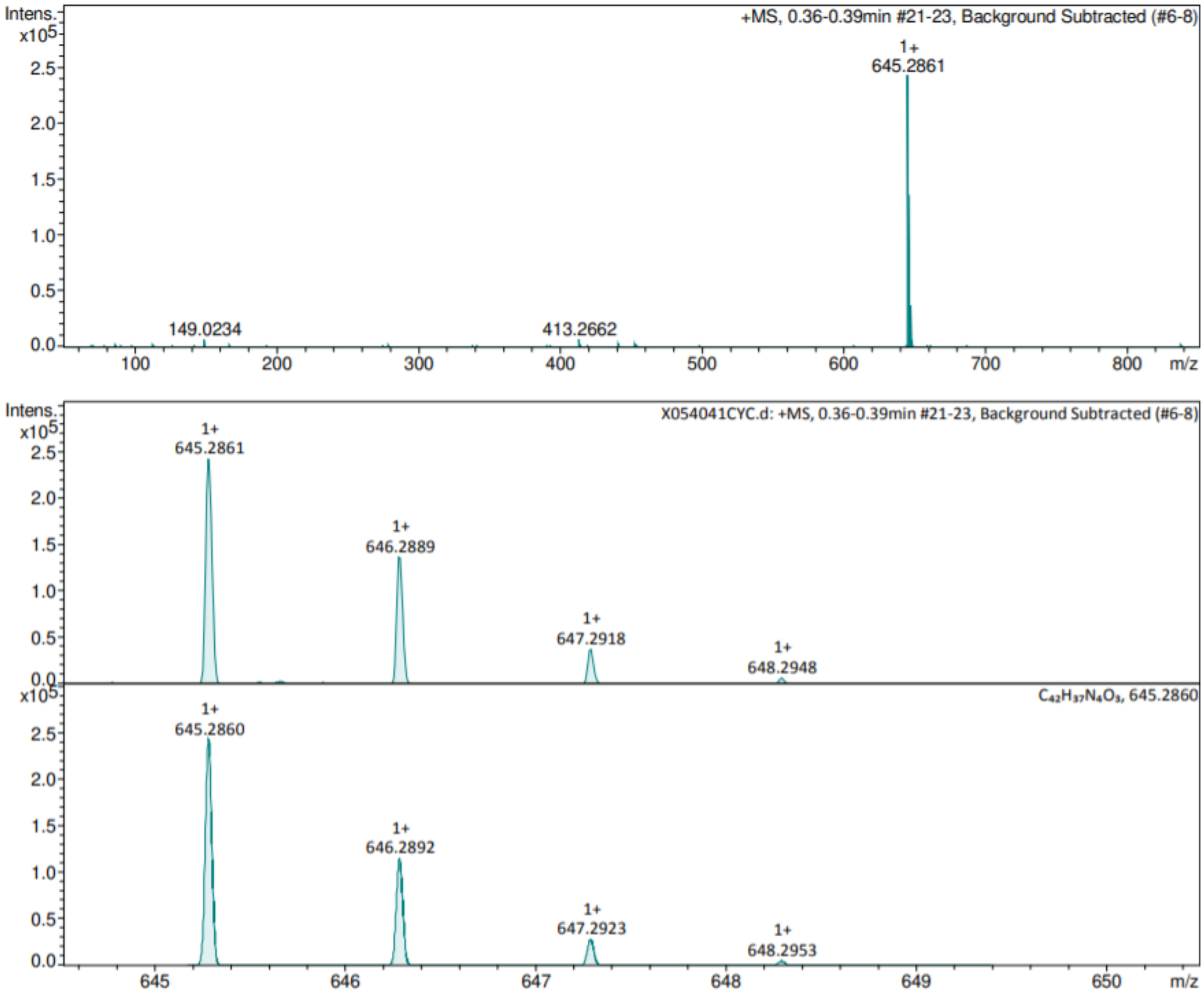

Figure S24. HRMS ESI+ of compound **6b**

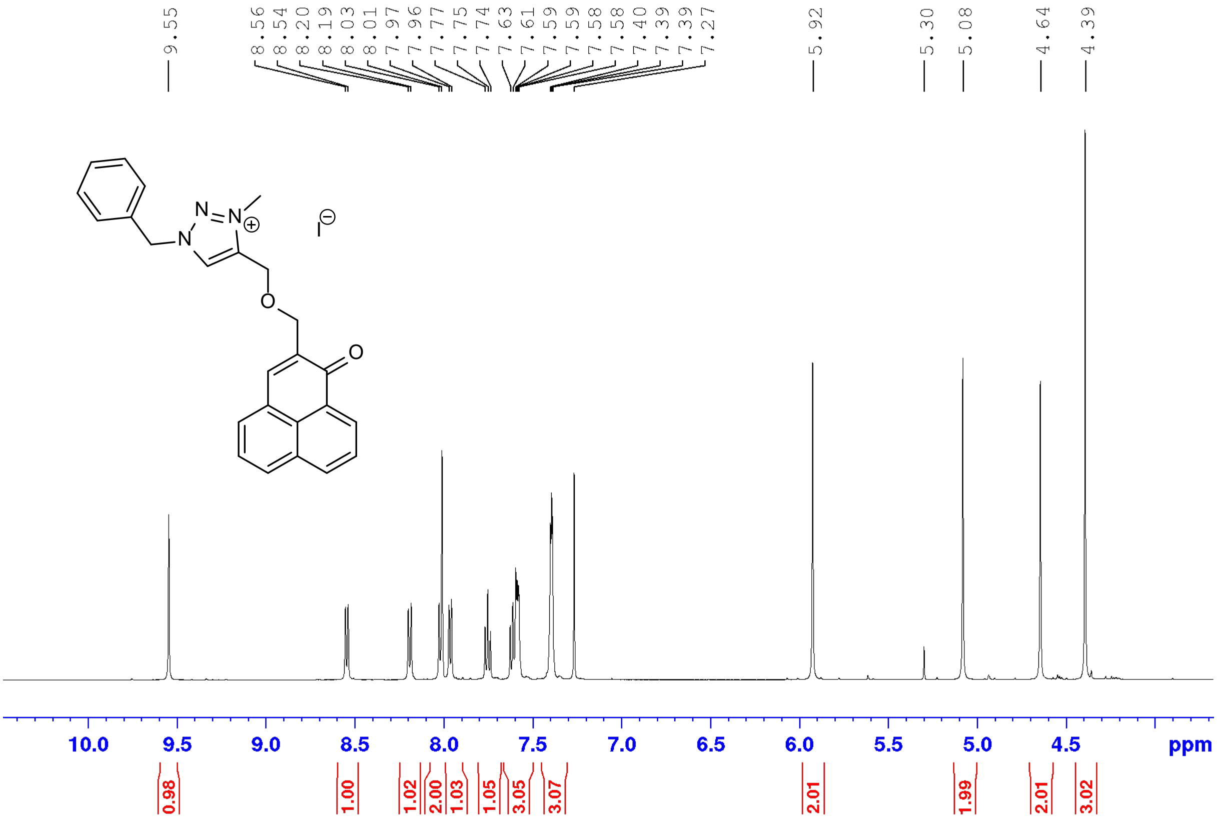

Figure S25. ^{1}H NMR of compound **7a** in $CDCl_3$

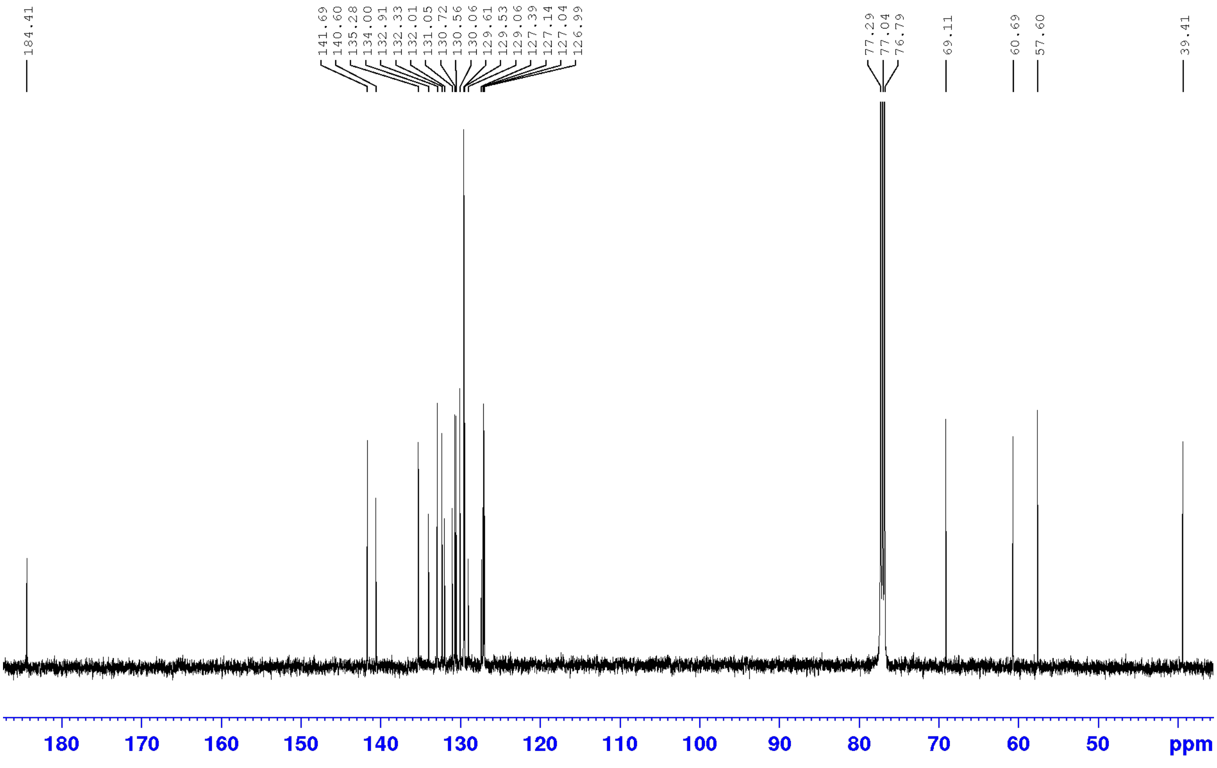

Figure S26. ^{13}C NMR of compound **7a** in $CDCl_3$

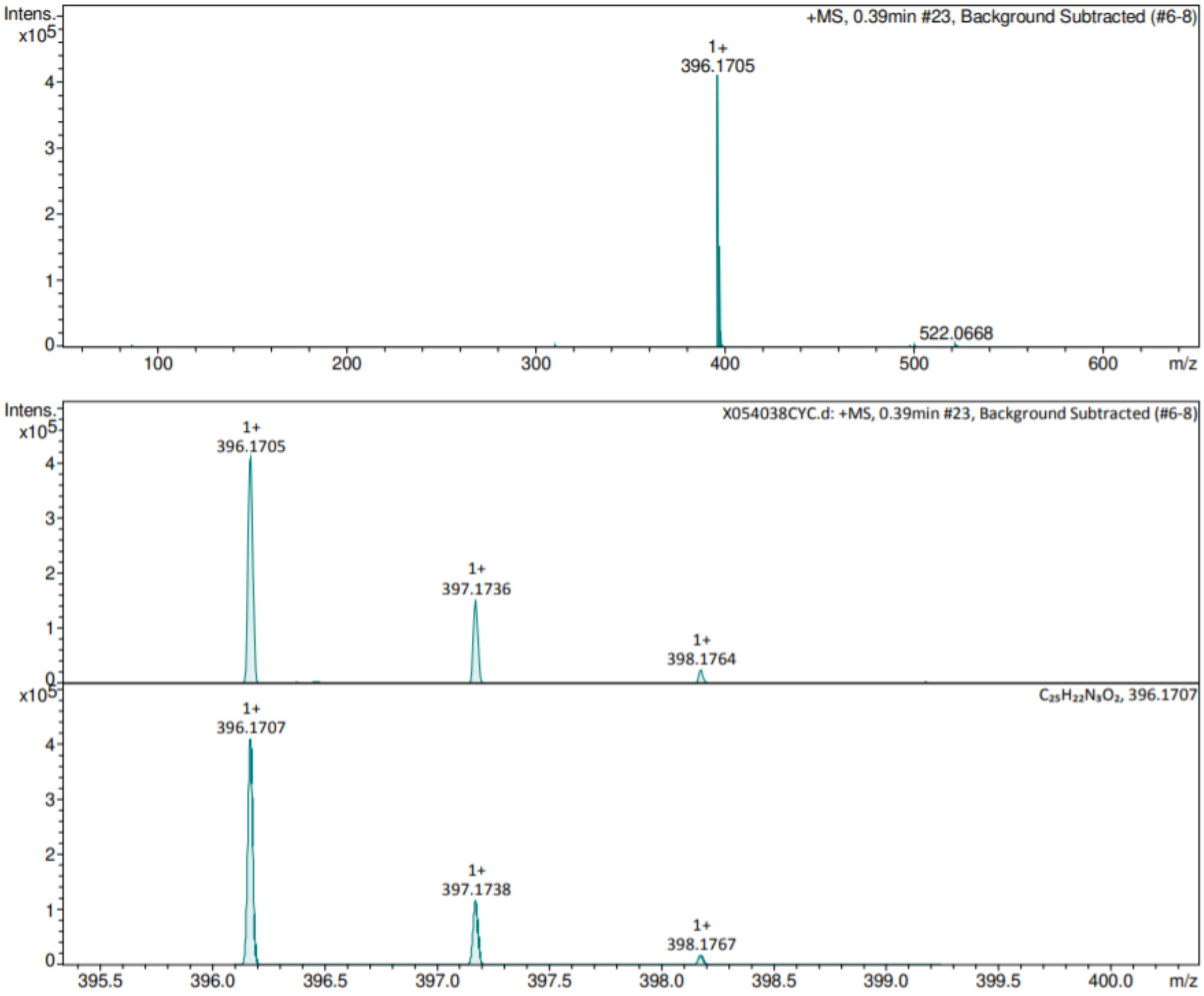

Figure S27. HRMS ESI$^+$ of compound **7a**

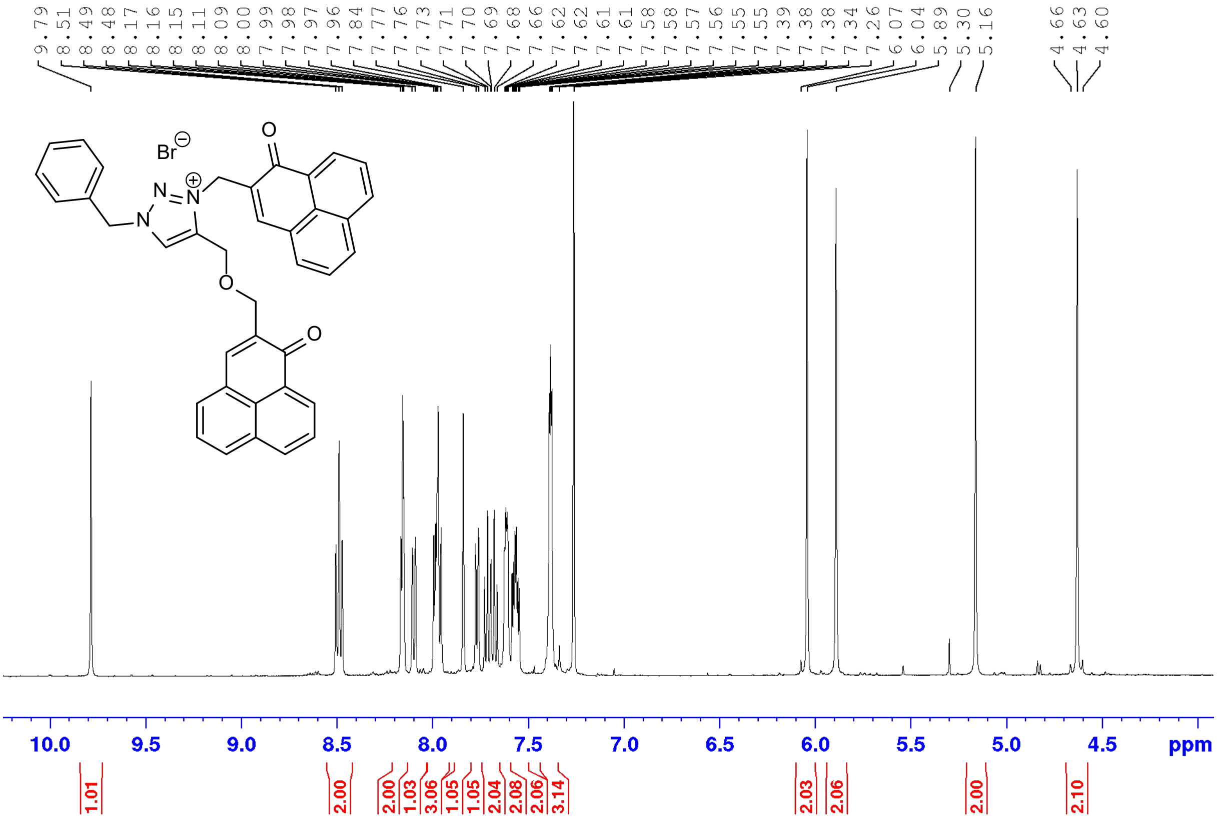

Figure S28. ^{1}H NMR of compound **7b** in $CDCl_3$

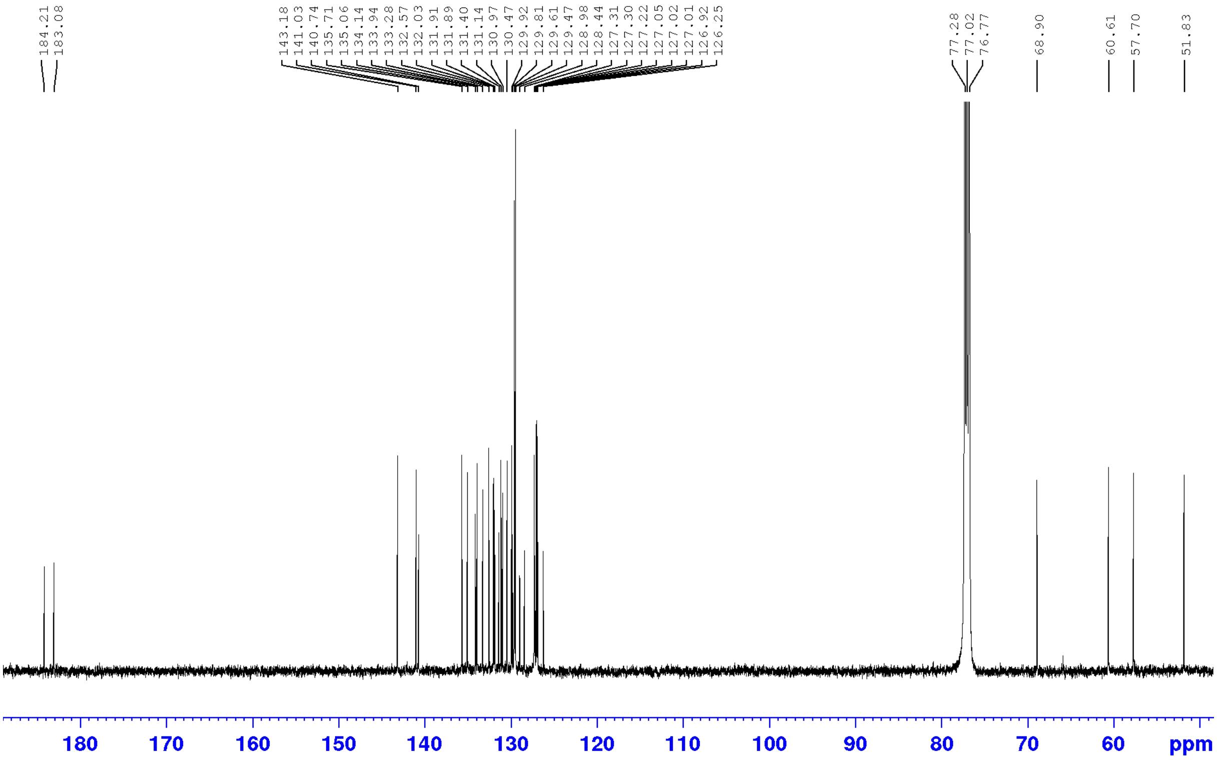

Figure S29. ^{13}C NMR of compound **7b** in $CDCl_3$

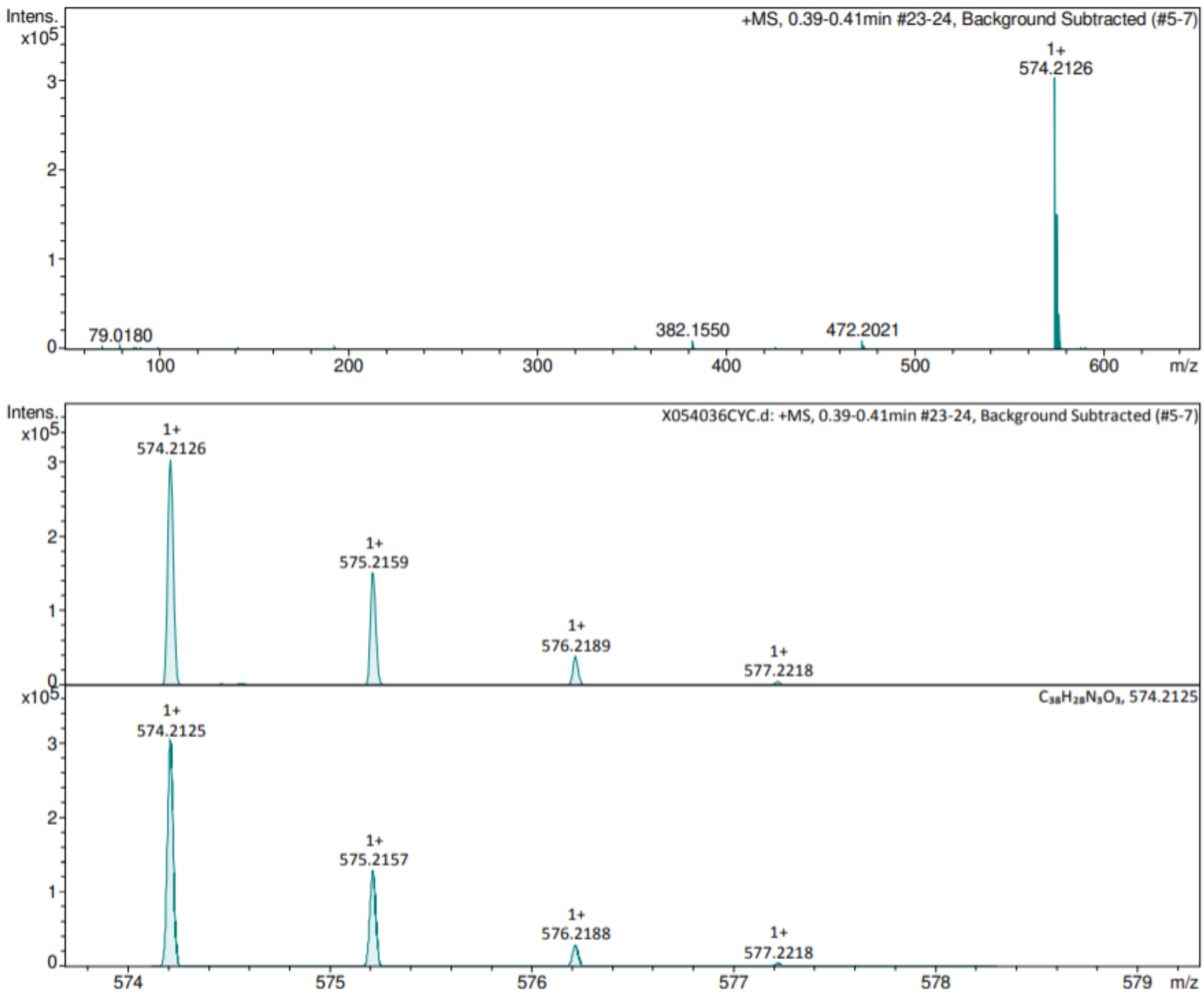

Figure S30. HRMS ESI+ of compound **7b**

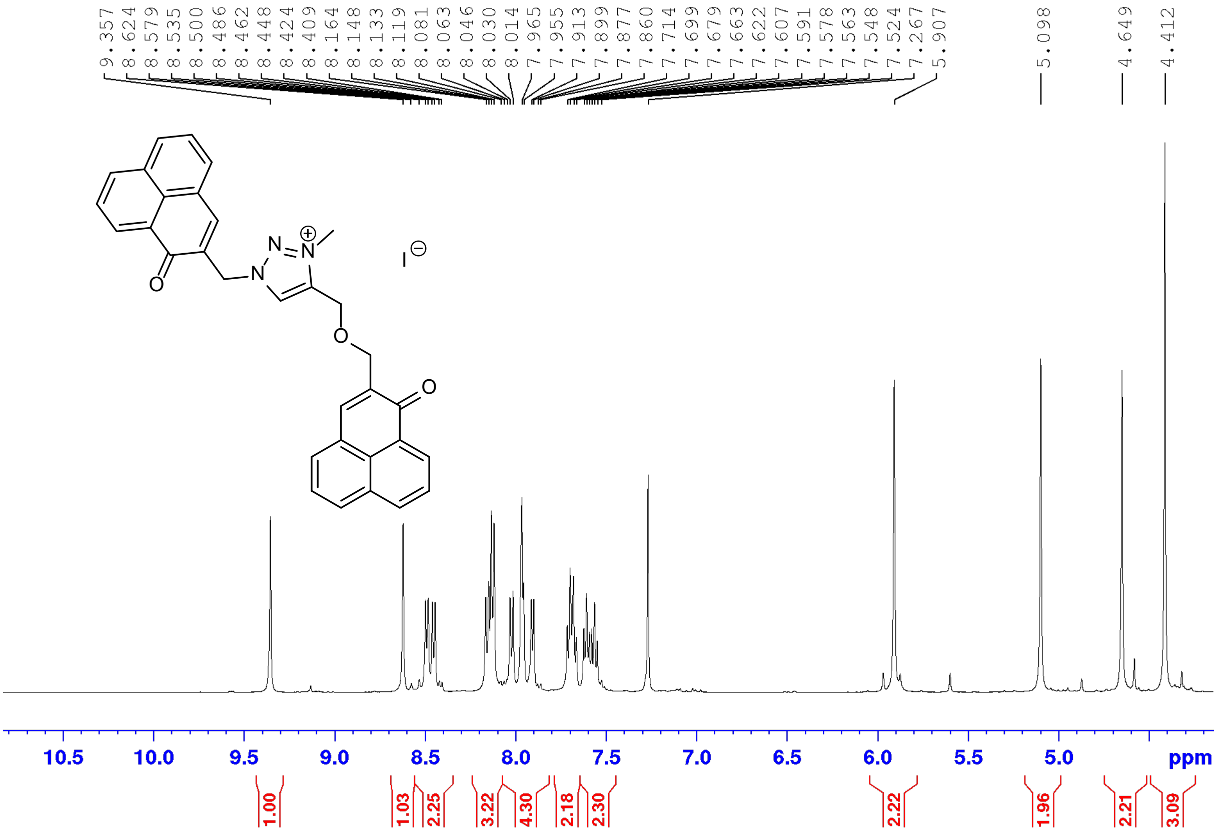

Figure S31. ^{1}H NMR of compound **8a** in $CDCl_3$

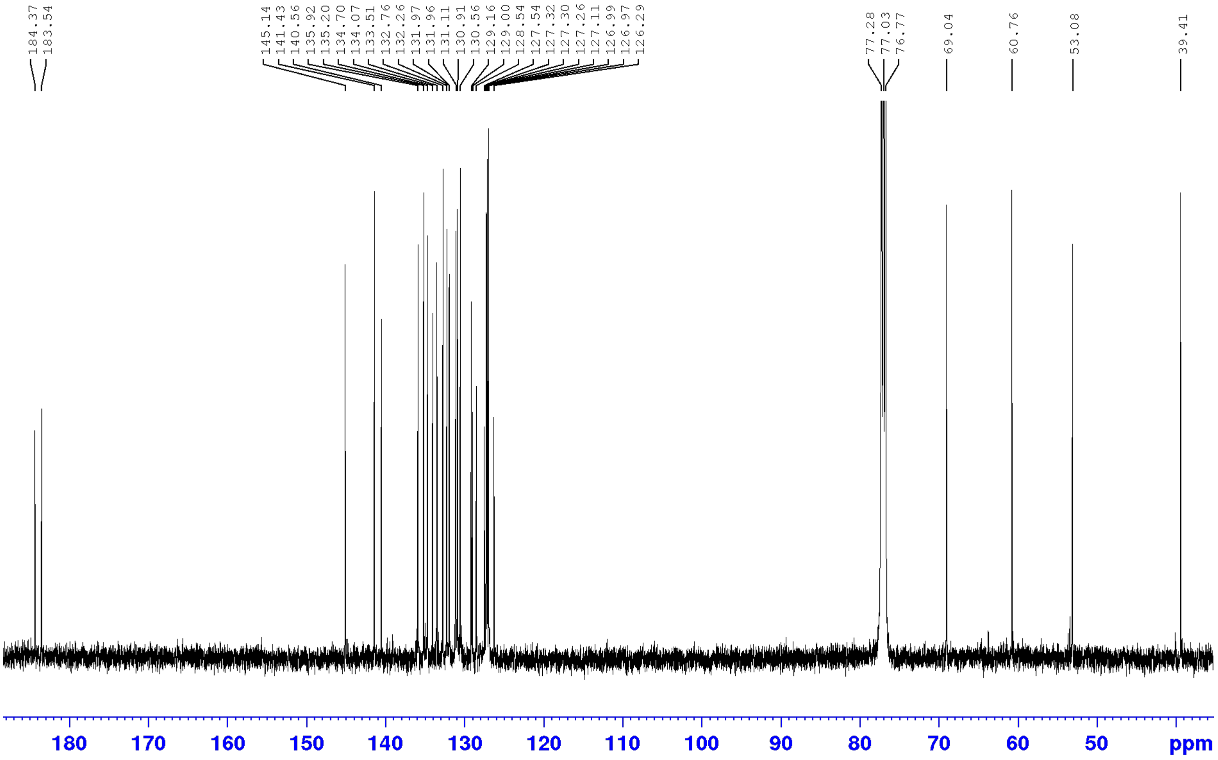

Figure S32. ^{13}C NMR of compound **8a** in $CDCl_3$

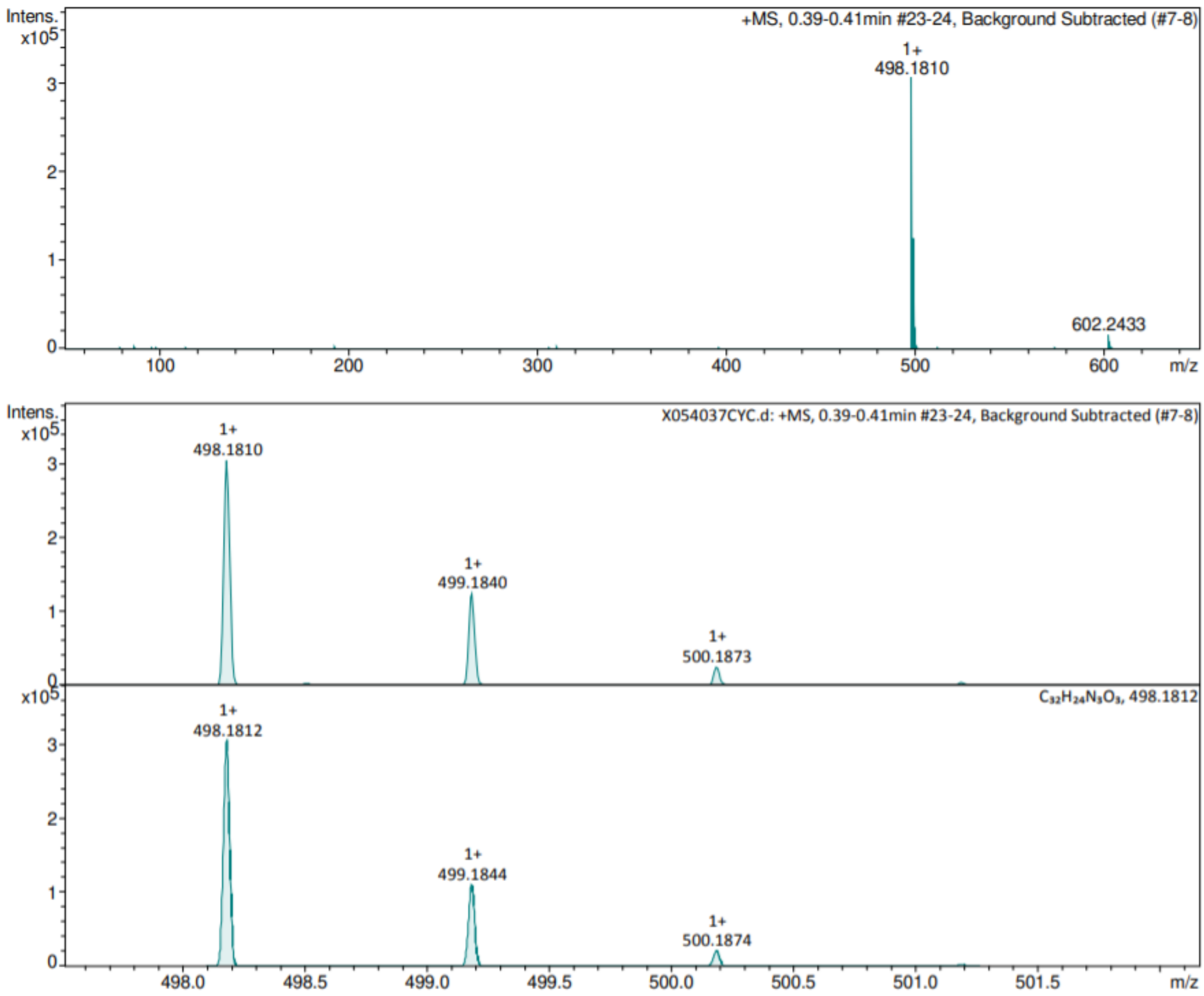

Figure S33. HRMS ESI$^+$ of compound **8a**

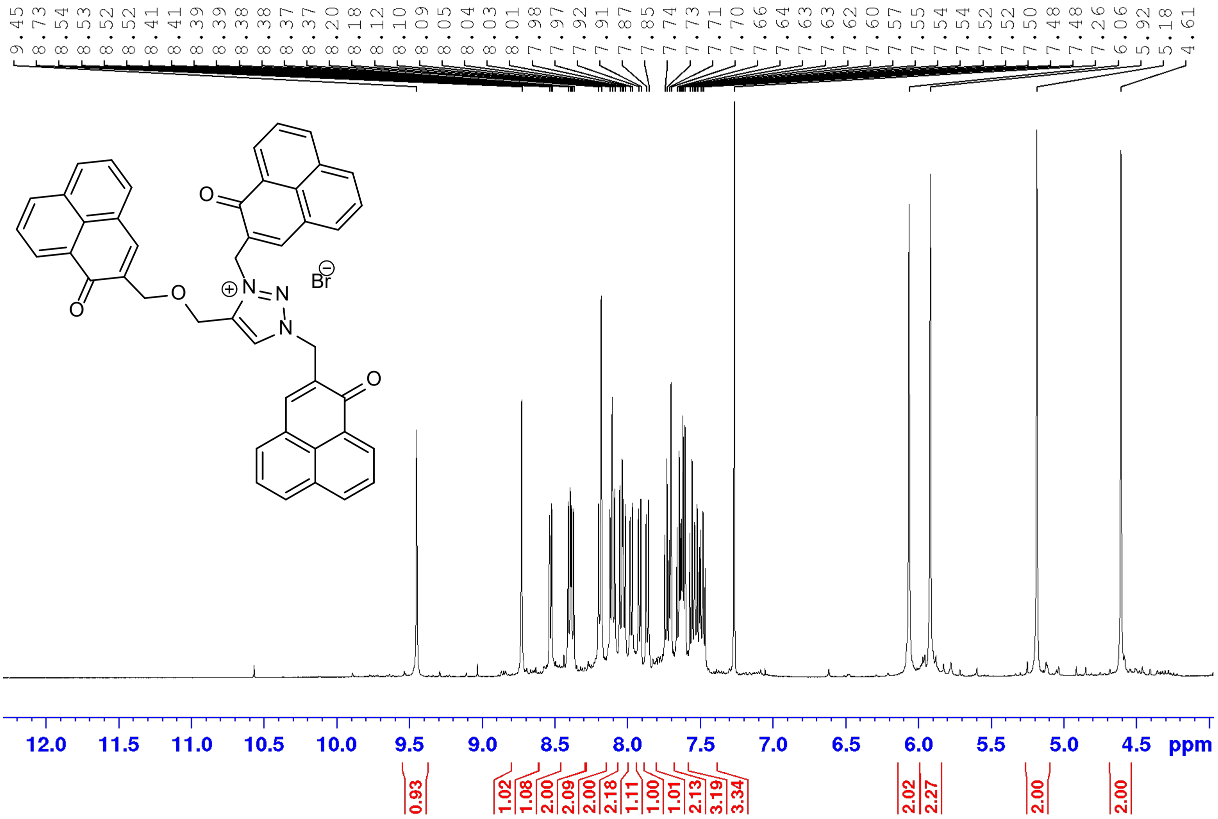

Figure S34. ^{1}H NMR of compound **8b** in $CDCl_3$

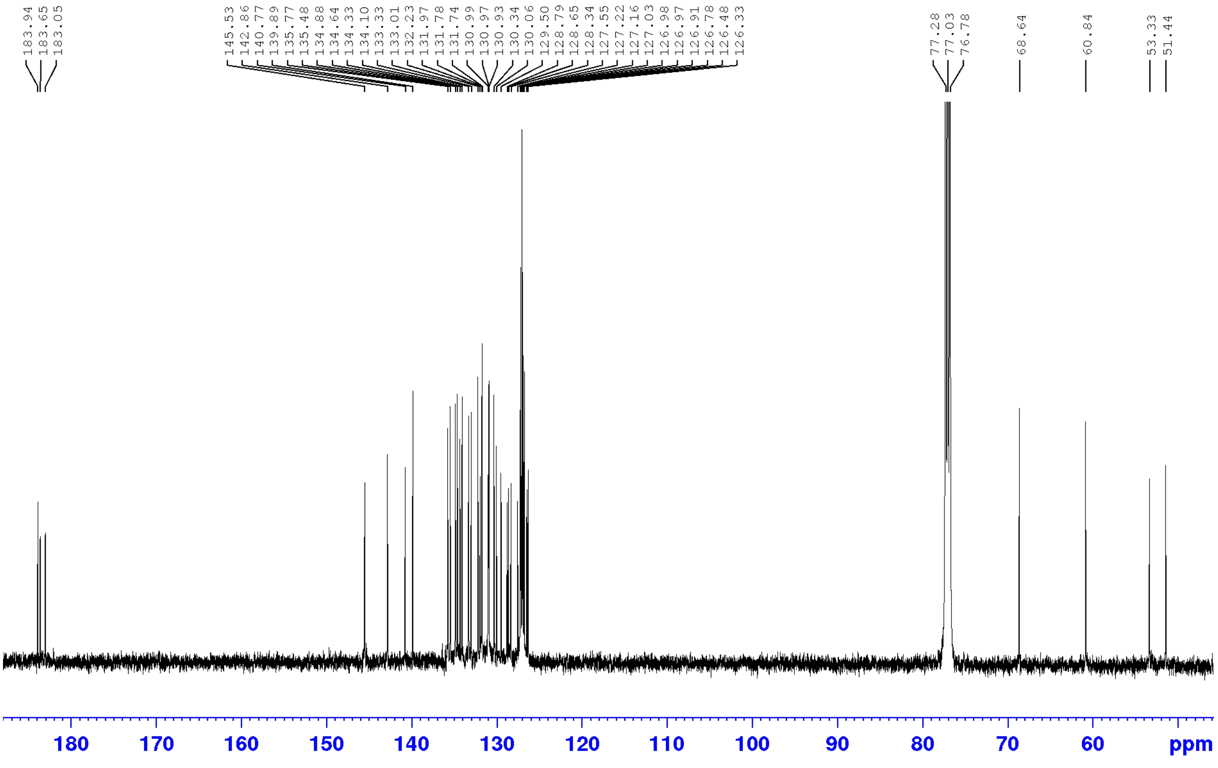

Figure S35. ^{13}C NMR of compound **8b** in $CDCl_3$

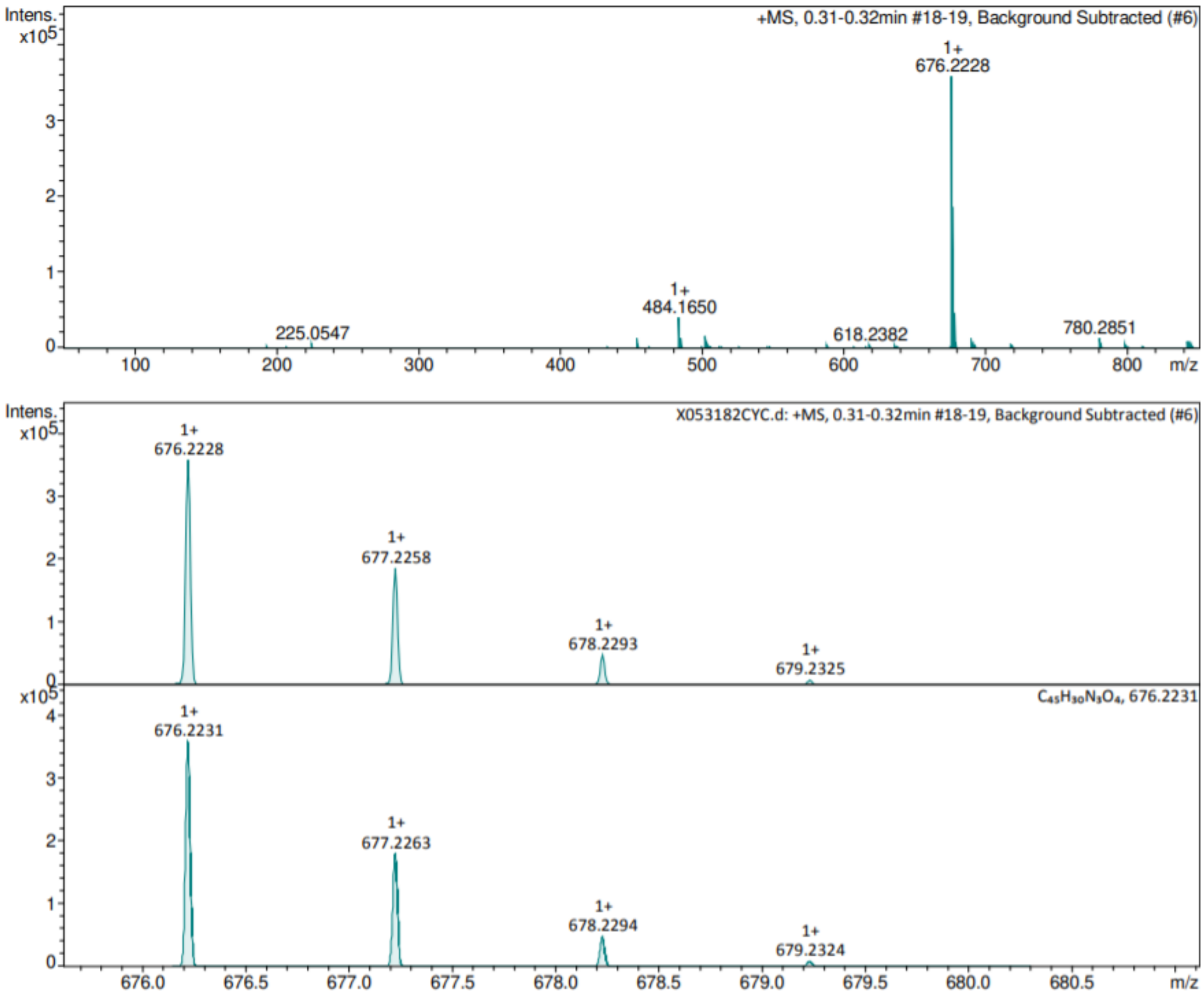

Figure S36. HRMS ESI+ of compound **8b**

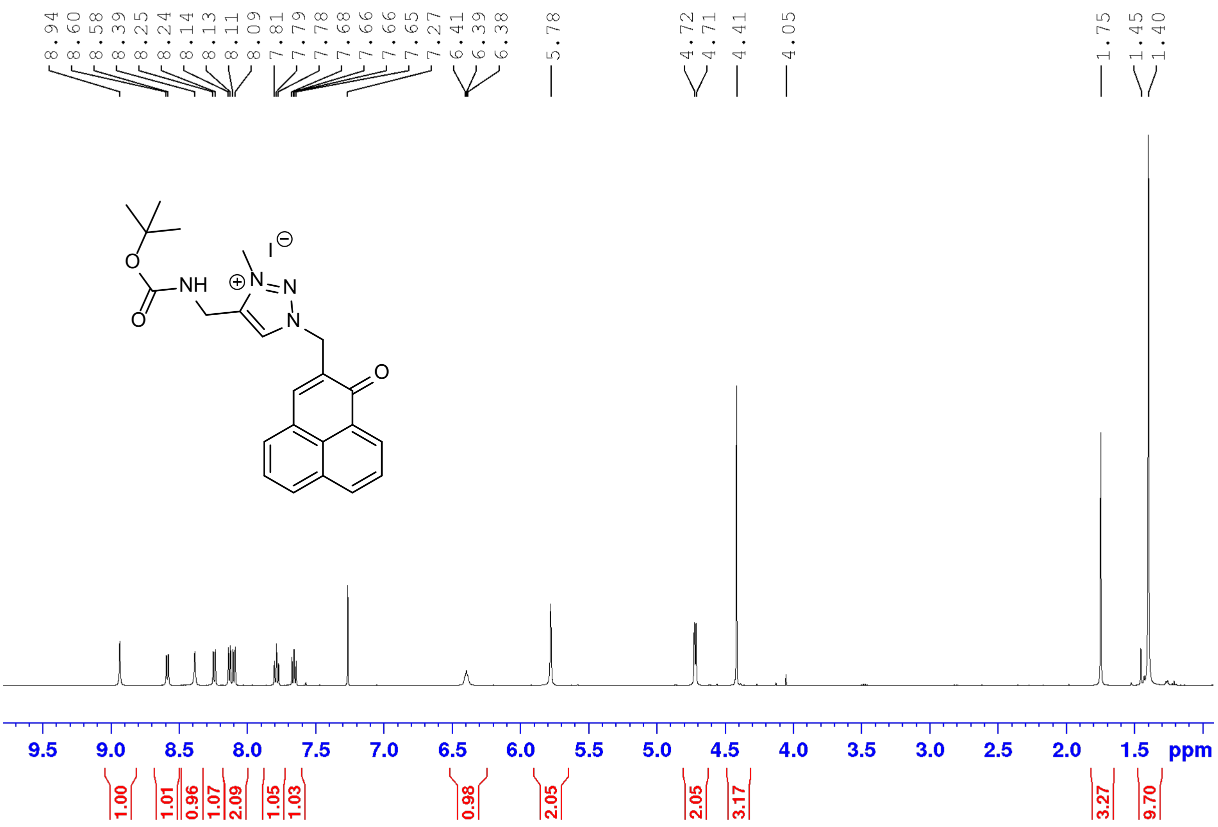

Figure S37. ^{1}H NMR of compound **9a** in $CDCl_3$

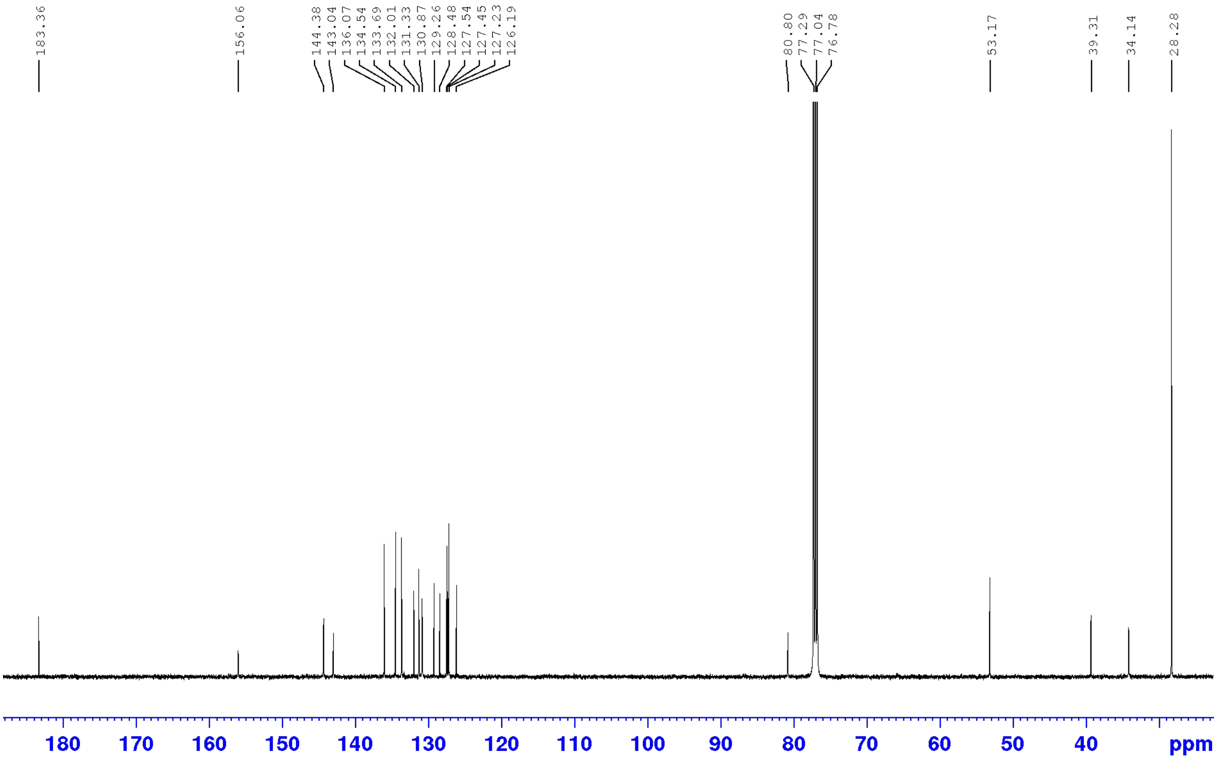

Figure S38. ^{13}C NMR of compound **9a** in $CDCl_3$

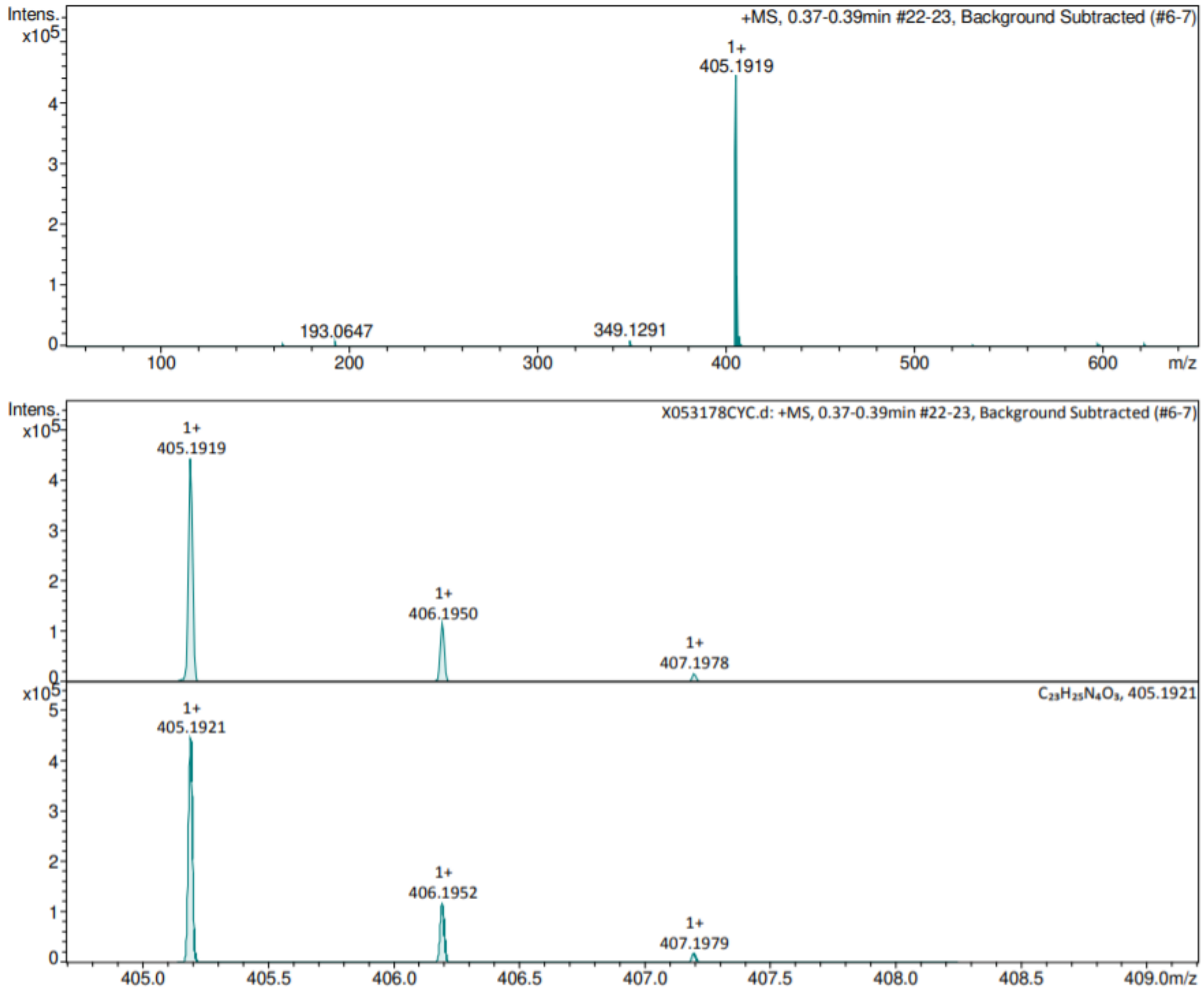

Figure S39. HRMS ESI$^+$ of compound **9a**

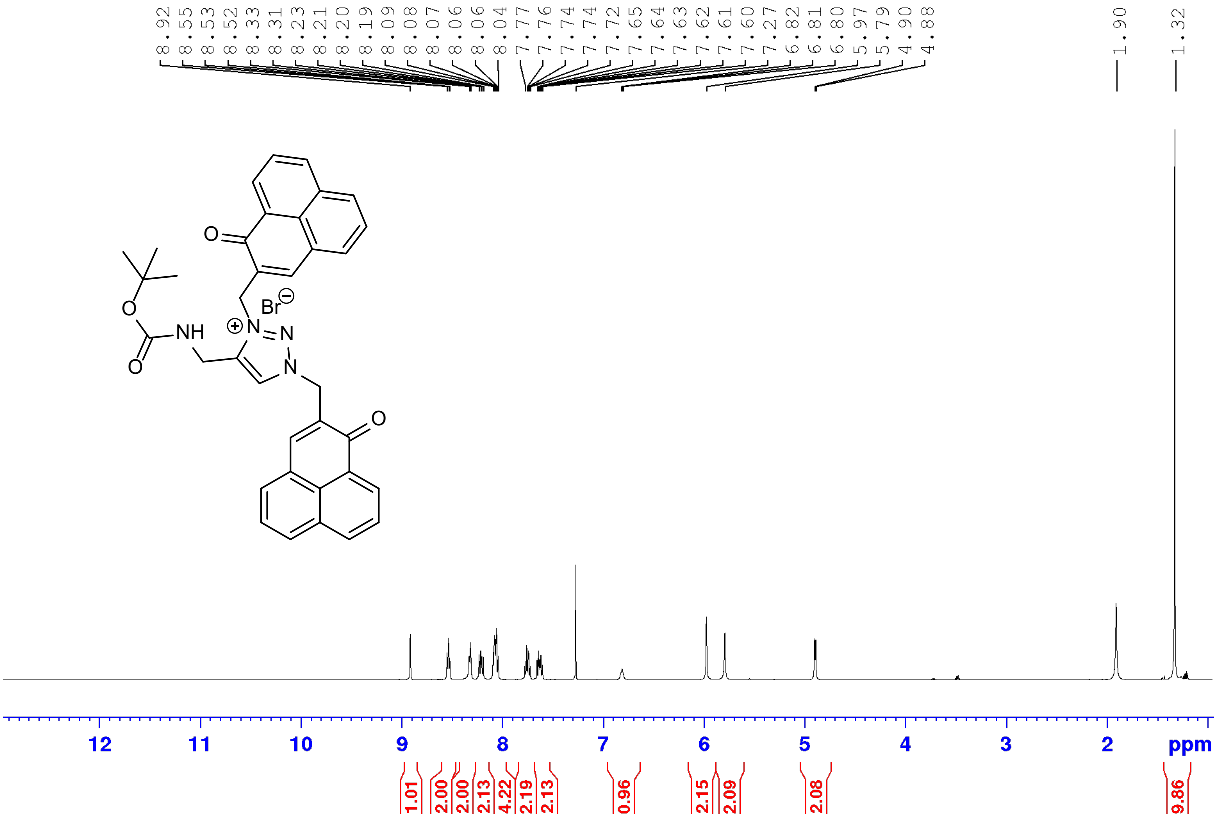

Figure S40. ^{1}H NMR of compound **9b** in $CDCl_3$

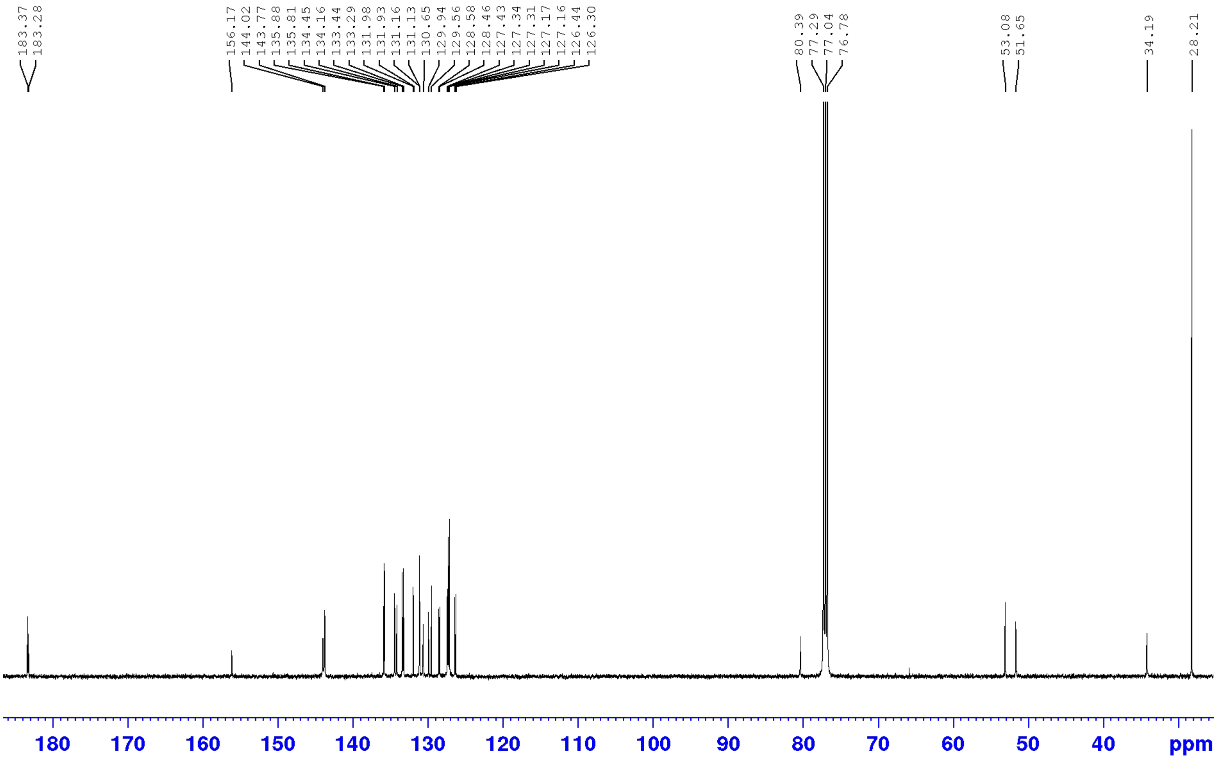

Figure S41. ^{13}C NMR of compound **9b** in $CDCl_3$

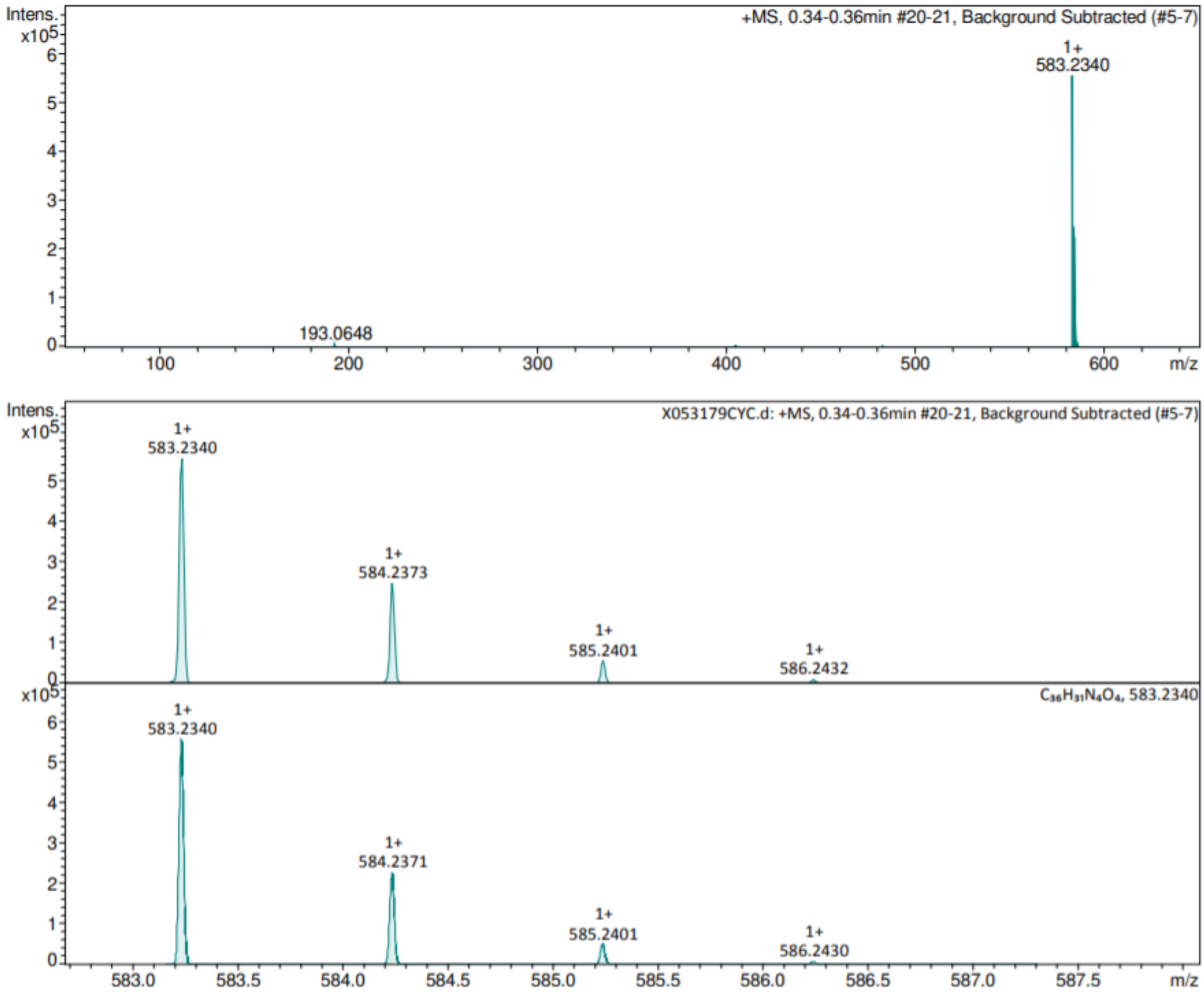

Figure S42. HRMS ESI+ of compound **9b**

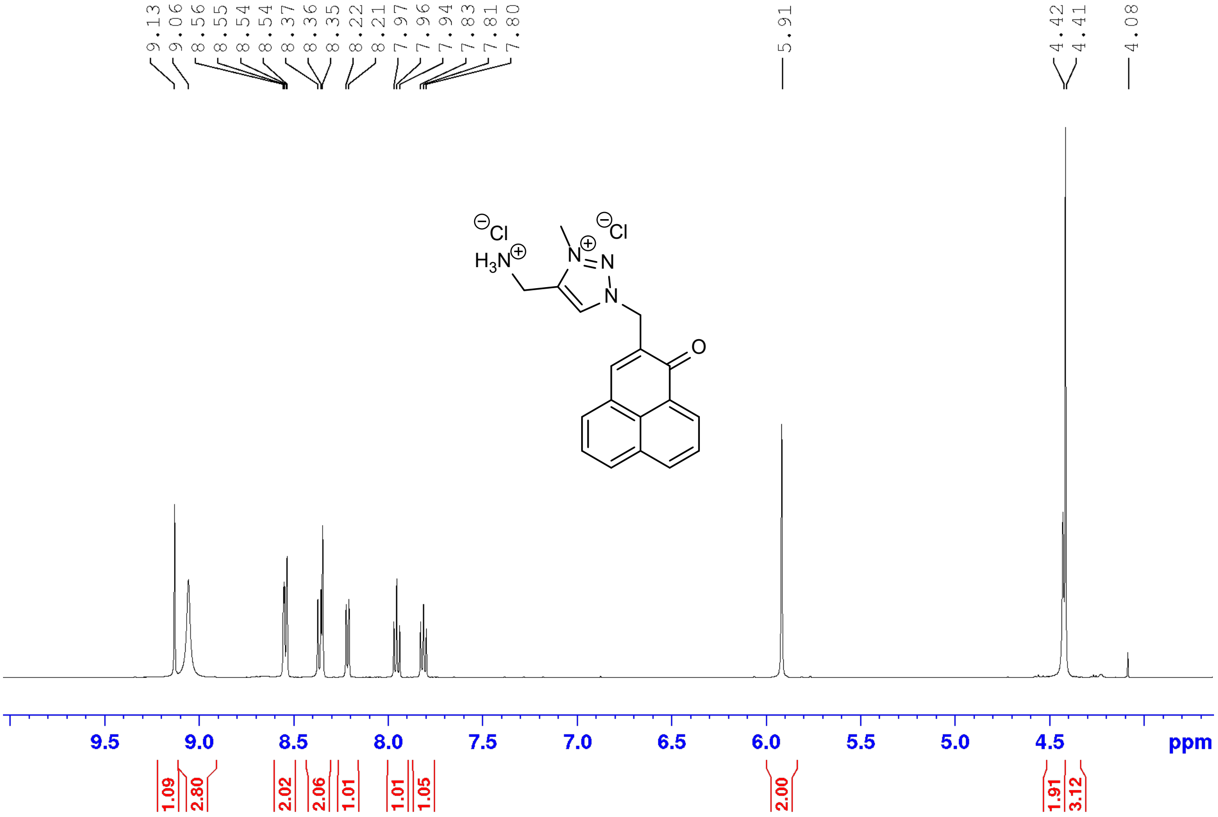

Figure S43. ^{1}H NMR of compound **10a** in DMSO-d_6

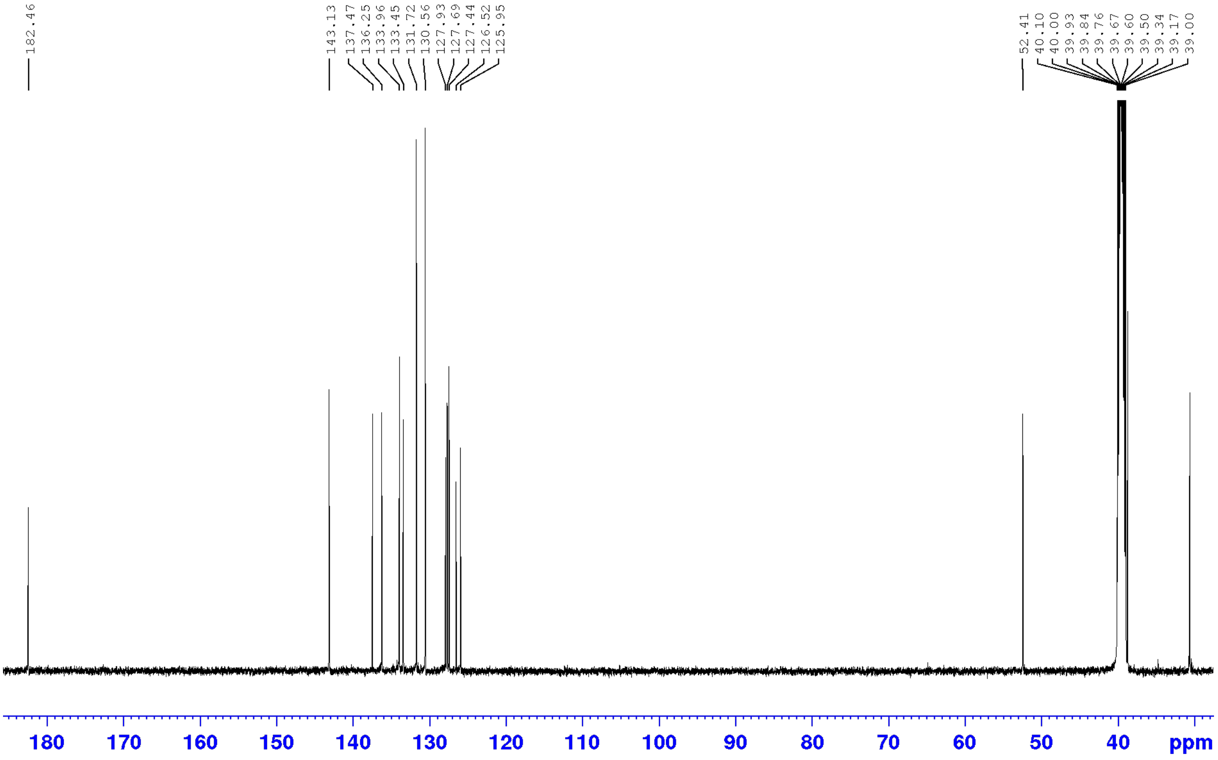

Figure S44. ^{13}C NMR of compound **10a** in DMSO-d_6

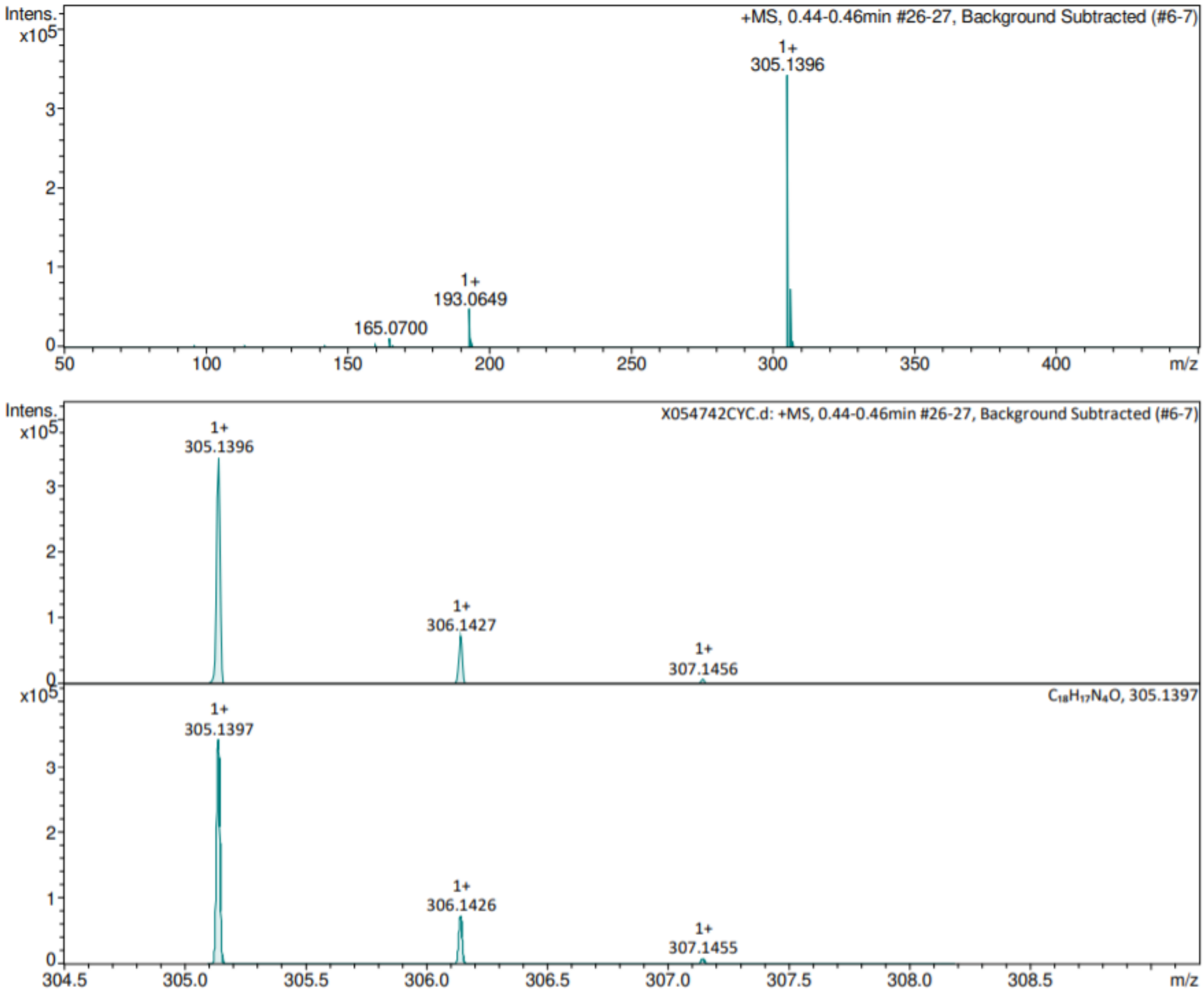

Figure S45. HRMS ESI+ of compound **10a**

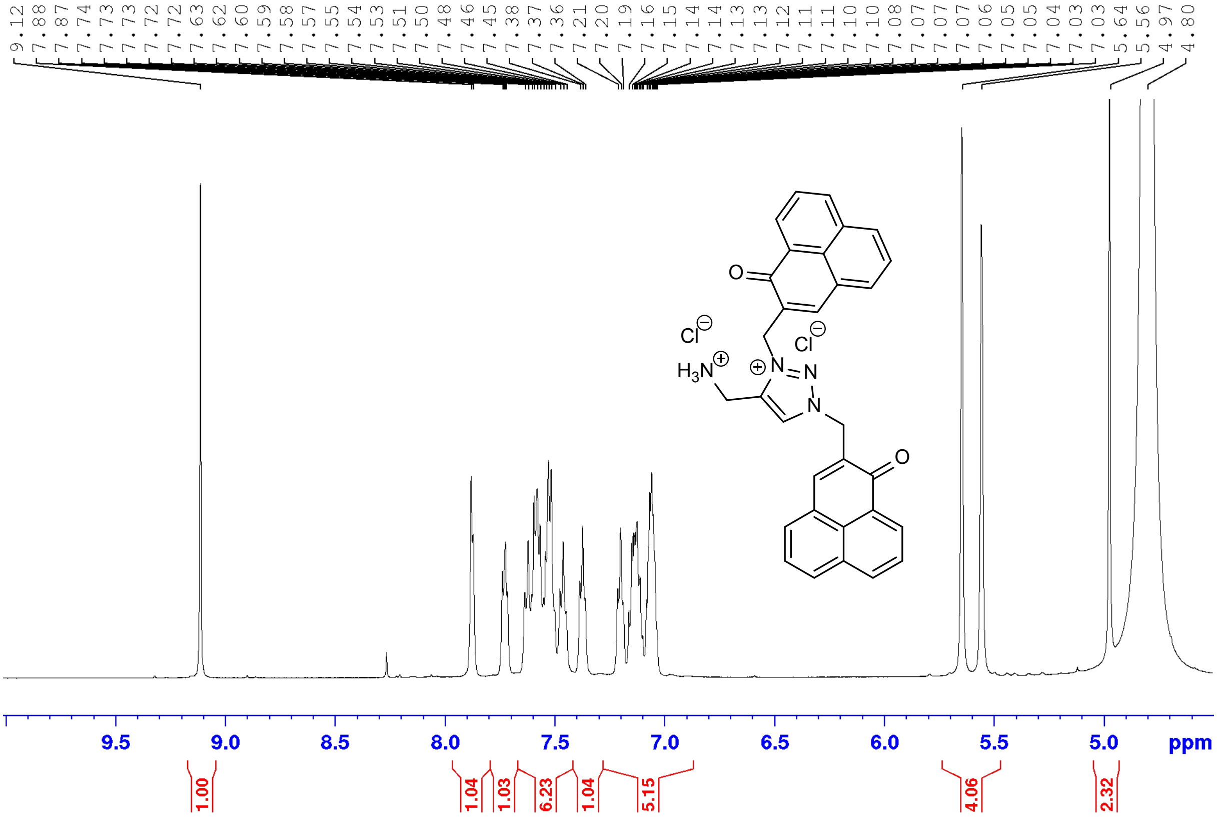

Figure S46. ^{1}H NMR of compound **10b** in D_2O

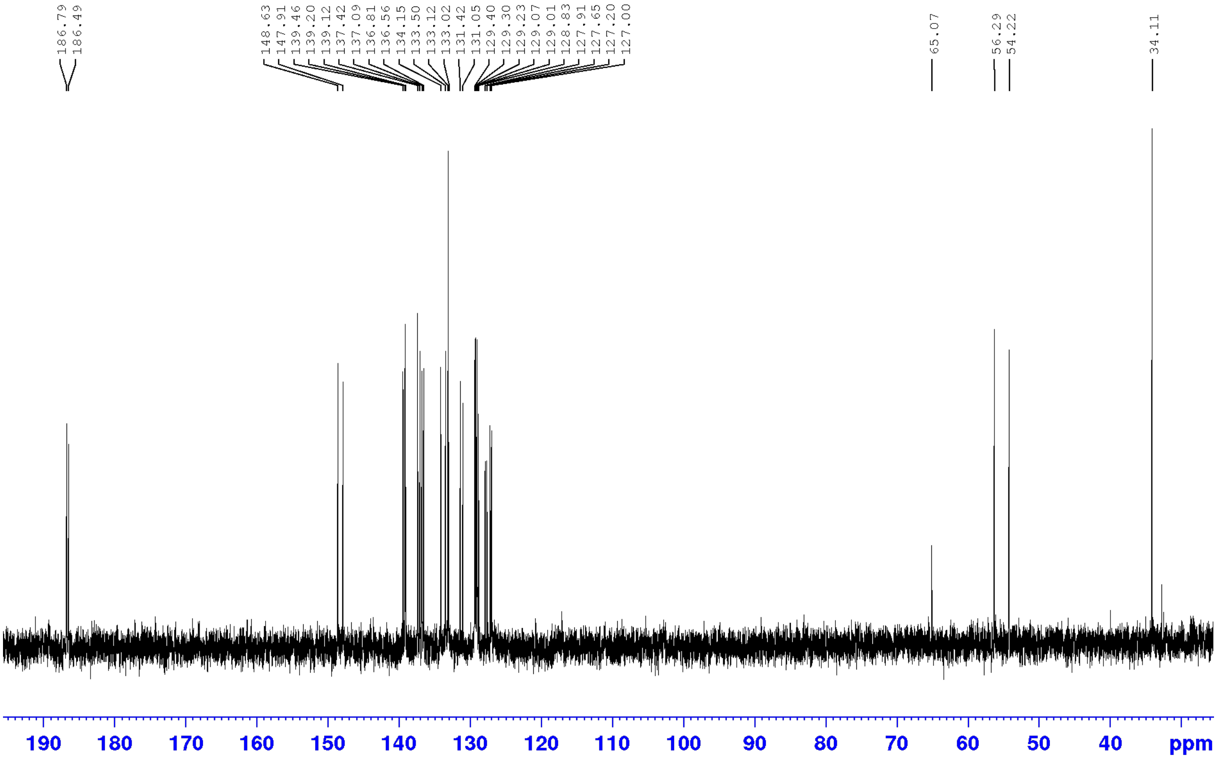

Figure S47. ^{13}C NMR of compound **10b** in D_2O

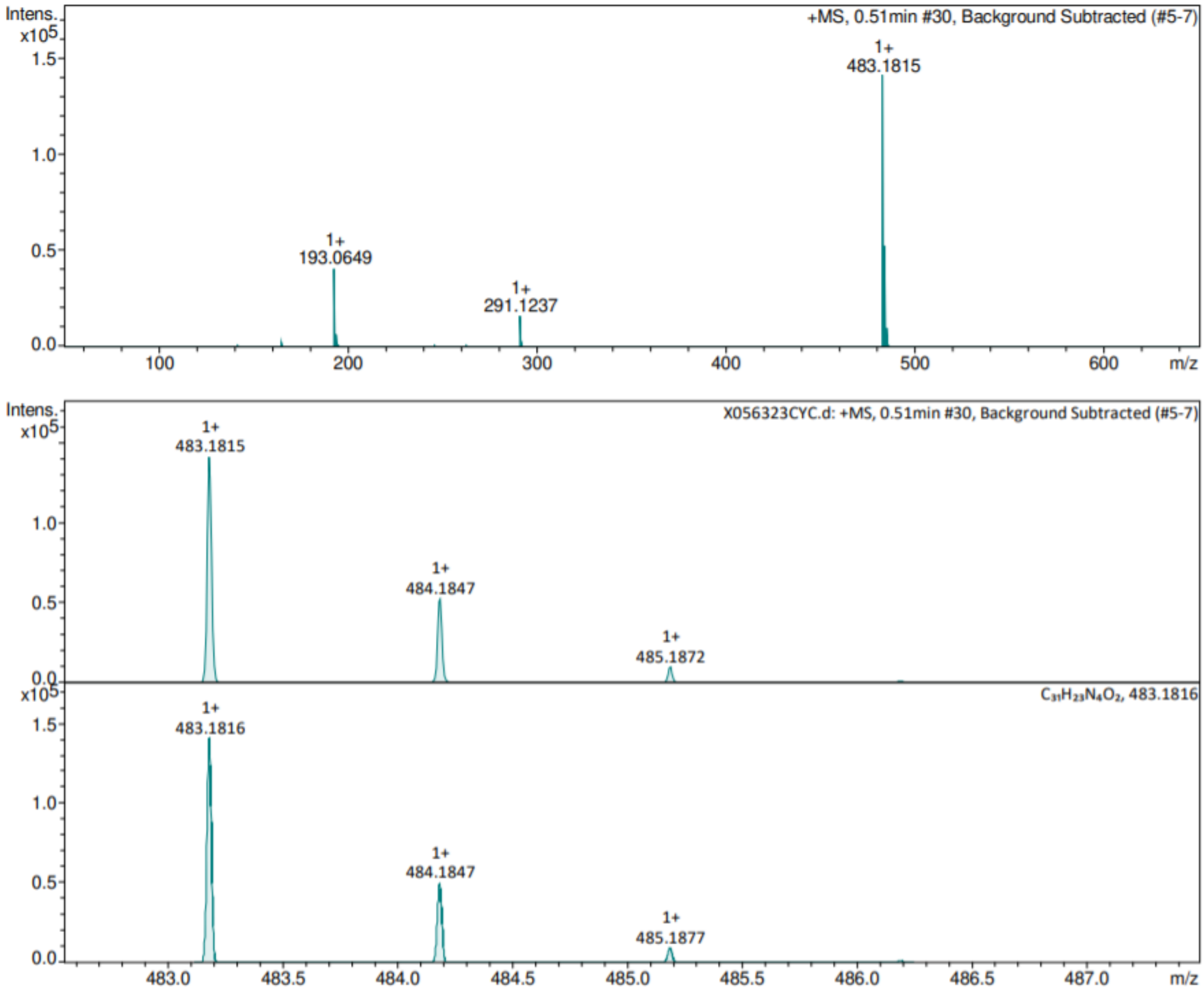

Figure S48. HRMS ESI+ of compound **10b**

Figure S49 : Chemical structures of all compounds

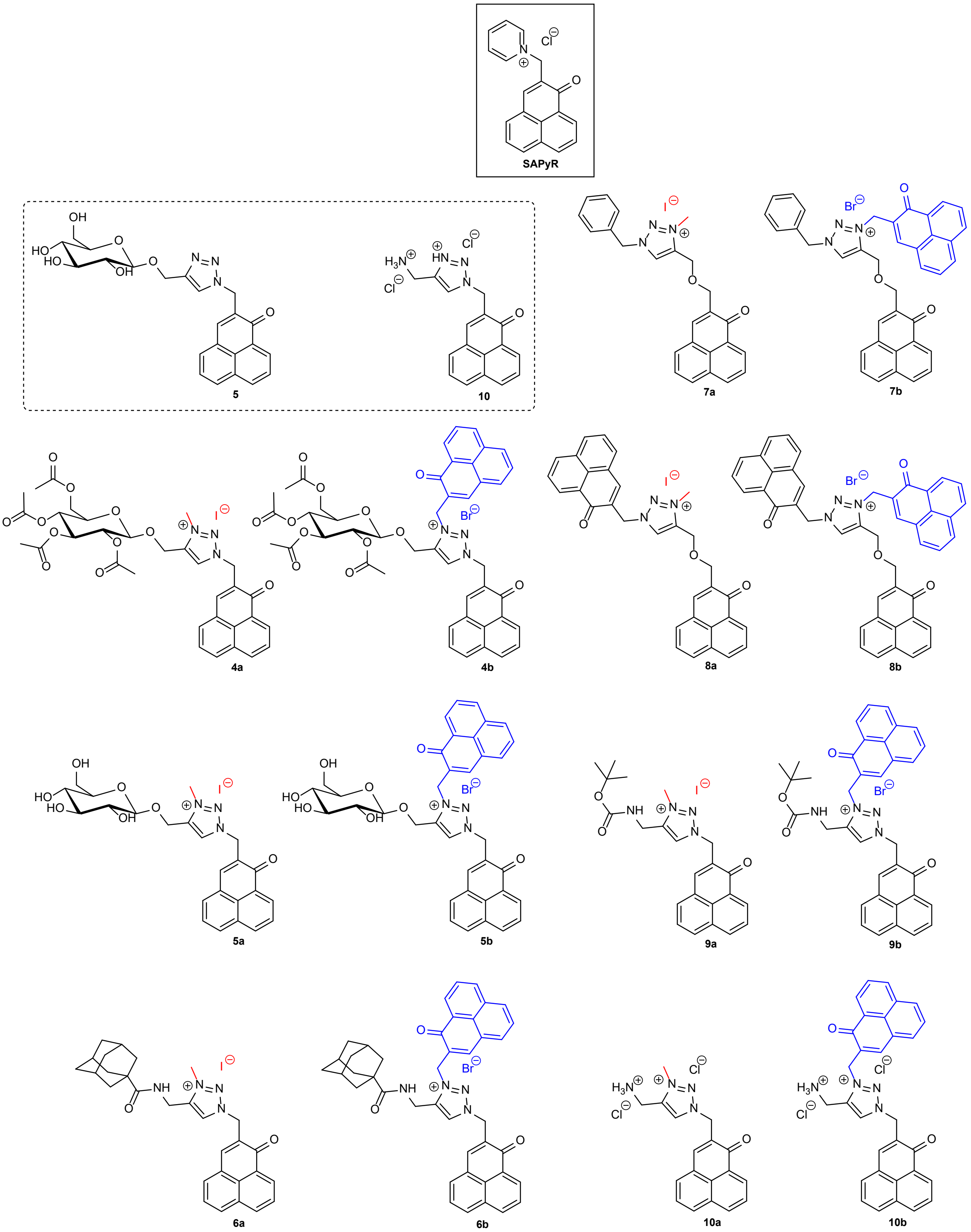

Supplement: Supplementary file 1 [file antibiotics-10-00626-s001.zip › antibiotics-1171526-supplementary.pdf]
